# Bi-allelic variants in *NDUFA5* cause a mitochondriopathy with complex I deficiency

**DOI:** 10.1016/j.ajhg.2026.03.003

**Published:** 2026-03-30

**Authors:** Natalie B. Tan, Matthias Gautschi, Michael Raum, Daniella H. Hock, Robert Kopajtich, Jia Wang, Xiao Qian, Tanavi Sharma, Timothy E. Green, Jean-Marc Nuoffer, Katrina M. Bell, Katarzyna Pospieszny, Tegan Stait, Chloe Pike, Michelle Cao, Susan M. White, David R. Thorburn, Theresa Brunet, Matias Wagner, Wolfgang Müller-Felber, Leopold Zeng, Thomas Klopstock, André Schaller, Jing Liu, David A. Stroud, Holger Prokisch

**Affiliations:** 1 Murdoch Children’s Research Institute, Parkville, VIC, Australia; 2 Victorian Clinical Genetics Services, Parkville, VIC, Australia; 3 Department of Paediatrics, The University of Melbourne, Parkville, VIC, Australia; 4 Department of Paediatrics, Division of Endocrinology, Diabetology and Metabolism, and Institute of Clinical Chemistry, Inselspital - University Hospital of Bern, University of Bern, Bern, Switzerland; 5 Institute of Human Genetics, School of Medicine and Health, Technical University of Munich, Munich, Germany; 6 Department of Biochemistry and Pharmacology, Bio21 Molecular Science and Biotechnology Institute, The University of Melbourne, Melbourne, VIC, Australia; 7 Institute of Neurogenomics, Helmholtz Zentrum München, Munich, Germany; 8 Cipher Gene Ltd, Chengdu, China; 9 University Institute of Diagnostic and Interventional Neuroradiology, Inselspital, University Hospital of Bern, University of Bern, Bern, Switzerland; 10 Division of Pediatric Neurology and Developmental Medicine and LMU Center for Children with Medical Complexity, Dr. von Hauner Children’s Hospital, LMU Hospital, Ludwig-Maximilians-Universität, Munich, Germany; 11 Friedrich Baur Institute at the Department of Neurology, LMU University Hospital, LMU Munich, 80336 Munich, Germany; 12 German Center for Neurodegenerative Diseases (DZNE), 81377 Munich, Germany; 13 Munich Cluster for Systems Neurology (SyNergy), 81377 Munich, Germany; 14 Department of Human Genetics, Inselspital - University Hospital of Bern, University Bern, Bern, Switzerland; 15 German Center for Child and Adolescent Health (DZKJ), Partner Site Munich, Munich, Germany

**Keywords:** NDUFA5, mitochondrial disease, mitochondriopathy, complex I deficiency, CI deficiency

## Abstract

*NDUFA5* encodes a structural subunit of mitochondrial complex I (NADH:ubiquinone oxidoreductase) located in the peripheral arm of the enzyme complex. Complex I is the largest enzyme of the mitochondrial respiratory chain and is essential for oxidative phosphorylation. There are many well-characterized conditions associated with nuclear-encoded mitochondrial complex I dysfunction, including Leigh syndrome, leukoencephalopathy, lethal infantile mitochondrial disease, hypertrophic cardiomyopathy, and exercise intolerance. The vast majority of these nuclear-encoded mitochondrial complex I deficiencies are autosomal-recessive conditions. To date, variants in *NDUFA5* have not been associated with mitochondriopathy in humans. We identified a cohort of four individuals from three unrelated families with bi-allelic variants in *NDUFA5*. All individuals present with variable multisystem disease in the setting of a mitochondrial complex I deficiency, biochemically proven via an array of respiratory chain enzymology, blue native PAGE, and mass-spectrometry-based proteomics in peripheral blood mononuclear cells, lymphoblastoid cell lines, fibroblasts, and skeletal muscle. Transcriptomics and RT-PCR demonstrated aberrant mRNA expression in all affected individuals. Finally, we generated zebrafish *ndufa5* F0 mutants that exhibited defects of morphological development, locomotor deficits, and abnormal brain activity. Our data demonstrate that bi-allelic variants in *NDUFA5* cause a mitochondrial complex I deficiency, characterized by a variable multisystem phenotype that encompasses severe congenital heart defects, hematological abnormalities, and neurological involvement consistent with Leigh syndrome.

## Teaser

We identify bi-allelic *NDUFA5* variants in four individuals from three families with mitochondrial complex I deficiency. Genomic, transcriptomic, proteomic, and biochemical studies across patient tissues, complemented by a zebrafish model, characterize a variable multisystem disease with cardiac, hematological, and Leigh syndrome-like neurological features, expanding the spectrum of complex I mitochondriopathies.

## Main text

The nuclear gene *NDUFA5* (MIM: 601677) encodes a structural subunit of mitochondrial complex I (NADH:ubiquinone oxidoreductase), specifically located in the peripheral arm (Q module) of the enzyme complex. Complex I is the largest enzyme of the mitochondrial respiratory chain and is essential for oxidative phosphorylation. Experimental ablation of *Ndufa5* in mice demonstrates that a requirement for embryonic survival and conditional knockout in the central nervous system leads to partial complex I deficiency, resulting in mild chronic encephalopathy and late-onset motor deficits but notably without increased oxidative damage or neuronal loss.^1^ Common single-nucleotide polymorphisms in *NDUFA5* have been associated with neurodevelopmental disorders such as autism in case-control and family-based studies.^2^ This suggests a potential role for *NDUFA5* in neurodevelopmental processes, likely through its impact on mitochondrial function.

Complex I is an L-shaped assembly composed of 45 subunits encoded by 44 genes in mammals, including 14 highly conserved core subunits responsible for catalysis, including seven encoded by mitochondrial DNA (mtDNA), and a set of supernumerary or accessory subunits that contribute to stability, regulation, and assembly.^3^ The enzyme contains a hydrophilic arm, which houses the NADH dehydrogenase active site and redox centers (flavin mononucleotide and multiple iron-sulfur clusters), and a membrane arm, which mediates proton translocation.^4^^,^^5^^,^^6^ The majority of complex I in humans is stably associated with complex III (coenzyme Q-cytochrome *c* reductase) and complex IV (cytochrome *c* oxidase) in respiratory chain supercomplexes (SCs), also known as the respirasome, of various stoichiometries.^7^^,^^8^^,^^9^ Functionally, complex I oxidizes NADH (produced by the tricarboxylic acid cycle and β-oxidation), reduces ubiquinone to ubiquinol, and pumps four protons per NADH from the mitochondrial matrix to the intermembrane space, thereby contributing to the electrochemical gradient used for ATP production.^6^ Dysfunction of complex I is implicated in a range of mitochondrial diseases, neurodegenerative disorders, and metabolic syndromes.^10^^,^^11^

Complex I deficiency is the most frequently encountered enzyme deficiency in mitochondrial disease, as it involves the largest respiratory chain complex and is composed of both nuclear DNA (nDNA) and mtDNA encoded subunits.^12^^,^^13^ Nuclear-encoded complex I dysfunction typically manifests as Leigh syndrome (MIM: 256000 and MIM: 500017), leukoencephalopathy, lethal infantile mitochondrial disease, hypertrophic cardiomyopathy, and exercise intolerance^12^; however, it is well recognized that genotype-phenotype correlation for complex I disorders is poor. Traditional means for diagnosis have employed measurement of enzyme function, which is reliant on the sampling of affected tissue that necessitates invasive biopsy of, for example, skeletal muscle, or the even more difficult to access tissues of the liver and heart. Until the advent of sequencing technologies, enzymatic testing could identify a complex I deficiency but without specificity as to the underlying heritable cause. Here, we utilized a blend of traditional respiratory chain enzymology with modern molecular approaches to functional genomics—inclusive of genomic sequencing, RNA sequencing (RNA-seq), and mass spectrometry proteomics, from several tissues—to build a compelling evidence base for a recessive mitochondriopathy caused by pathogenic variants in *NDUFA5*.

In a collaborative effort facilitated via GeneMatcher,^14^ we assembled a cohort of four individuals from three unrelated families with bi-allelic variants in *NDUFA5*. All procedures were followed in accordance with the ethical standards of the responsible committees on human experimentation at each center. Informed consent was obtained for each study participant. Detailed clinical descriptions and genetic histories are described in supplemental notes: case reports.

**Figure 1:**
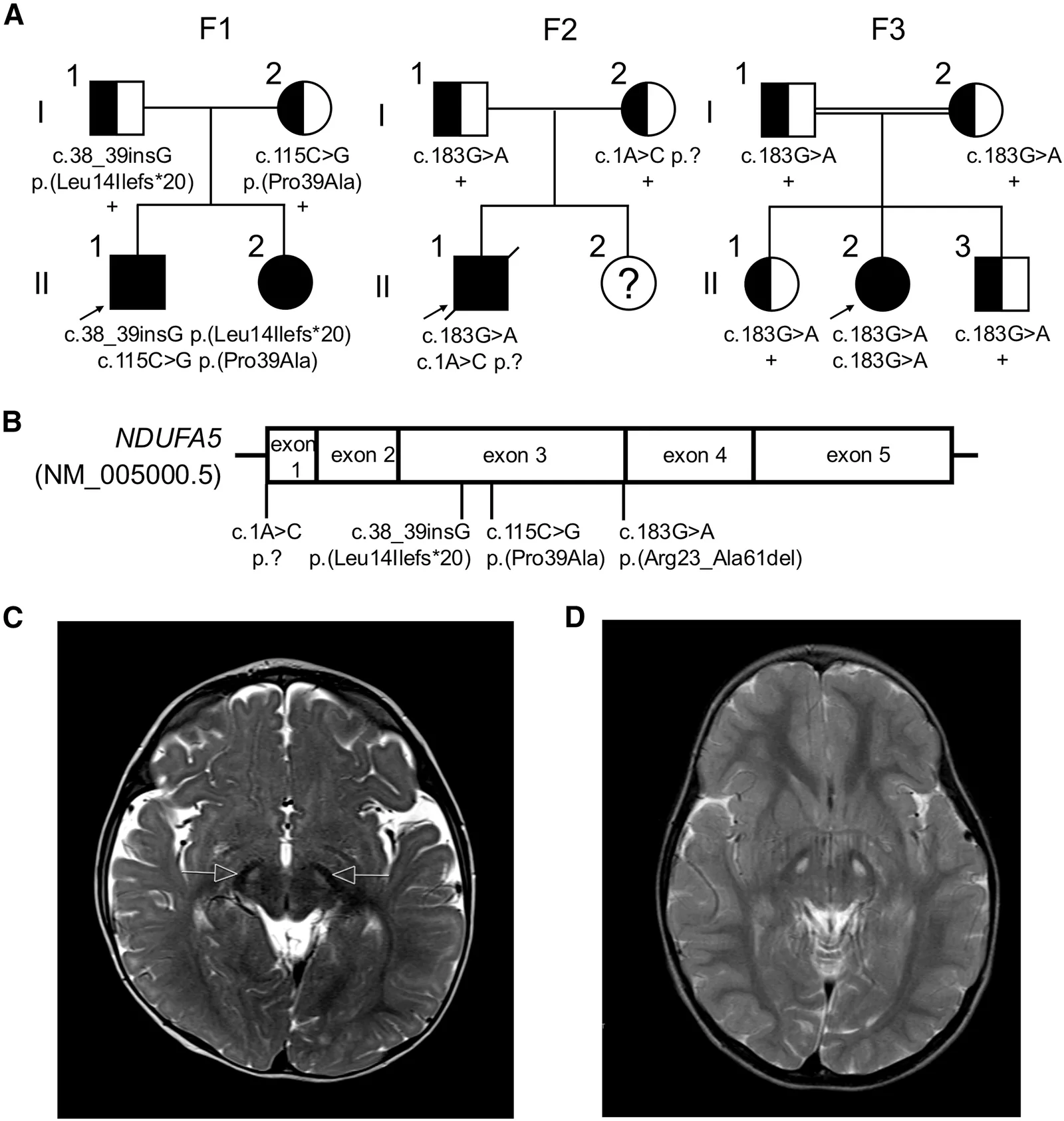
Bi-allelic variants in *NDUFA5* segregate with disease (A) Pedigrees for the three families described in this study. Consanguineous F3:I-1 and F3:I-2 are second cousins. (B) Schematic showing the *NDUFA5* gene and protein structure based on NCBI reference sequence GenBank: NM_005000.5. The location of variants described in this study are shown underneath the schematic. (C) Brain MRI at 11 months of age for individual F2:II-1. (D) Brain MRI at 3 years of age for individual F3:II-2.

Family 1 comprises two affected siblings (F1:II-1 and F1:II-2) born to non-consanguineous parents of Korean descent (Figure 1A). Both siblings presented prenatally with intrauterine growth restriction and congenital heart disease, specifically coarctation of the aorta and biventricular hypertrophy confirmed postnatally, respectively. Notably, they exhibited a distinctive hematological phenotype at birth, with pancytopenia (anemia, thrombocytopenia, and leukopenia) and congenital red cell macrocytosis that required multiple red cell transfusions. Both siblings continue to experience a persistent but fluctuating neutropenia and lymphopenia (Table 1). While early developmental concerns were raised in the context of recovery from a complicated neonatal course, F1:II-1 has demonstrated gradual catch-up to largely age-appropriate milestones by age 2 years. Quad whole-genome sequencing (WGS) identified compound heterozygous variants in *NDUFA5* (Figure 1B): a maternally inherited highly conserved missense variant c.115C>G (p.Pro39Ala) that is ultra-rare in gnomAD v.4.1.0 with no homozygotes or alternative missense substitutions for Pro39, and a paternally inherited frameshift variant c.38_39insG (p.Leu14Ilefs^∗^20) that is also ultra-rare with no reported homozygotes (Table 1).

**Table 1:** Clinical phenotype information

**Individual**	**Family 1**		**Family 2**		**Family 3**
	**II-1**	**II-2**	**II-1**		**II-2**
Sex	M	F	M		F
Age	2.6 years (CGA 2.5 years)	6 months (CGA 5 months)	13 years (deceased)		19 years
Zygosity	compound heterozygous		compound heterozygous		homozygous
*NDUFA5* variants (GenBank: NM_005000.5)	maternal c.115C>G (p.Pro39Ala)	paternal c.38_39insG (p.Leu14Ilefs^∗^20)	maternal c.1A>C (p.0?)	paternal c.183G>A (p.Arg23_Ala61del)	biparental c.183G>A (p.Arg23_Ala61del)
Variant type	missense	frameshift	start-loss	synonymous/splice	synonymous/splice
gnomAD v.4.1.0 (AF; AC; hmz)	0.000001242; 2; 0	0.000003100; 5; 0	0.0002559; 413; 0	0.00001043; 16; 0	0.00001043; 16; 0
AlphaMissense	0.413	N/A	N/A	N/A	N/A
CADD	25.3	33	20.8	17.9	17.9
REVEL	0.617	N/A	0.452	N/A	N/A
PolyPhen2	0.778	N/A	0.979	N/A	N/A
SpliceAI Δ score^a^	0.01	0.04	0.01	0.12	0.12
Gestation	36 + 6 weeks	36 + 6 weeks	42 weeks		39 weeks
Prenatal	IUGR (<1st centile); fetal ECHO (21 + 2): moderate to severe hypoplastic aortic arch; left to right heart size discrepancy (LV smaller than RV)	IUGR (≪1st centile); fetal ECHO (35 + 5): distally tortuous aortic arch without clear obstruction, mildly small left-sided structures with an apex forming LV, mild biventricular hypertrophy with preserved systolic function	N/A		N/A
Growth	small stature, preserved head circumference (OFC 36%, wt 1%, lt 6%)	small stature, preserved head circumference (OFC 17%, wt 5%, lt <1%)	microcephaly (progressive)		normal
Development	mild developmental delay (predominantly language), age-appropriate catch-up by 2 years	normal development (at CGA 4 months)	normal cognition (non-verbal IQ 137 at 12 years), initially normal motor development, progressively delayed after 10 months		normal
Metabolic	neonatal hyperlactatemia (resolved by ∼6 months)	neonatal hyperlactatemia (resolved)	lactate levels normal to max. 3.6 mmol/L		elevated lactate levels normal to max. 6.1 mmol/L
Neurology	N/A	N/A	generalized hypotonia, ataxia (2 years), developmental motor regression triggered by intercurrent infection, progressive myopathy (stiffness), respiratory and gastrointestinal dysfunction (gastrostomy)		cerebellar ataxia; neuropathy; recurrent migraine headache
Ocular	N/A	N/A	infantile horizontal nystagmus (10 months); bilateral external ophthalmoplegia (2 years); bilateral optic neuropathy		nystagmus; bilateral optic atrophy
Hematology	pancytopenia (anemia, thrombocytopenia, leukopenia) on initial FBC at birth; congenital red cell macrocytosis (requiring multiple red cell transfusions, first on day 12); persistent but fluctuating cytopenias (neutropenia, lymphopenia)	congenital macrocytic anemia (requiring red blood cell transfusion at 1 month); lymphopenia (mild CD4^+^ T cell and CD19^+^ B cell); persistent neutropenia	normal blood counts, normal coagulation tests		normal blood counts, normal coagulation tests
Cardiology	congenital heart disease: coarctation of the aorta (repair 8 days of life, intraoperative thymectomy), ASD; persistent pulmonary arterial hypertension due to elevated biventricular end diastolic pressures; sinus node dysfunction (“chaotic atrial rhythm” in the postoperative setting, now resolved)	congenital heart disease: tortuous aortic arch, bicuspid aortic valve, small fenestrated ASD, mild LPA stenosis; mild biventricular hypertrophy (abnormal appearance of myocardium)	recurrent supraventricular tachycardia in light of a pre-excitation syndrome; hypertrophic cardiomyopathy (LV)		N/A
Other	congenital hirsutism (resolved); sacral dimple (spinal ultrasound NAD); left vocal cord palsy; hypospadias with bilateral hydroceles (awaiting surgery)	congenital hirsutism (resolved); sacral dimple, not midline (spinal ultrasound NAD); tongue tie release (1 month)	transitory increase of liver enzymes with intercurrent viral infection, consistently normal function and imaging, normal renal function		bilateral cavus foot deformity with hallux valgus
Investigations	brain MRI (3 weeks): mildly prominent ventricles; urine metabolic screen and organic acids: consistent with lactic acidosis; plasma amino acids: elevated alanine, otherwise non-diagnostic profile; normal chromosome breakage studies; normal free and total carnitine	normal hip ultrasound (breech at 31 + 6); normal screening cranial ultrasound; normal screening abdominal ultrasound	brain MRI/MRS (11 months): focal T2 signal alteration in the subthalamic region of the transition from the capsula interna to the pedunculus cerebri, as well as in the dorsomedial pons on both sides; increased patchy lactate peaks in the frontal to (markedly pronounced) parietal white matter; muscle biopsy: profound isolated complex I deficiency		brain MRI (3 years): two oval-shaped hyperintensities detected in the substantia nigra, along with a subtle band-like periaqueductal signal increase; muscle biopsy: profound isolated complex I deficiency

CGA, corrected gestational age; IUGR, intrauterine growth restriction; ECHO, echocardiogram; LV, left ventricle; RV, right ventricle; OFC, occipitofrontal circumference; wt, weight; lt, length; ASD, atrial septal defect; NAD, no abnormality detected; AF, allele frequency; AC, allele count; hmz, homozygotes; N/A, not applicable.

a SpliceAI Δ score is the delta score of a variant, defined as the maximum of (DS_AG, DS_AL, DS_DG, DS_DL). Generated with unmasked scores and maximum distance of 5,000 bp.

Family 2 is a male (F2:II-1) born to non-consanguineous Swiss parents (Figure 1A). He presented at 10 months of age with new-onset horizontal nystagmus, mild grasp asymmetry, and instability while sitting, initially attributed to an enterovirus infection but progressing to generalized muscular hypotonia, bilateral ophthalmoplegia, and hypertrophic cardiomyopathy (Table 1). There was normal to mildly increased lactate (maximum 3.6 mmol/L). Brain magnetic resonance imaging (MRI) (Figure 1C) and MR spectroscopy (MRS) were performed one month later, which demonstrated symmetric focal T2 signal alterations corresponding to diffusion restriction on diffusion-weighted imaging in the subthalamic region of the transition from the capsula interna to the pedunculus cerebri as well as in the dorsomedial pons on both sides, together with increased patchy lactate peaks in the frontoparietal white matter, all findings suggestive of an underlying mitochondrial disorder. Respiratory chain enzyme studies on skeletal muscle biopsy and skin fibroblasts both showed an isolated complex I deficiency (Table 2). Gastrotomy feeding was commenced at 2.5 years of age due to increased feeding difficulties and failure to thrive. A ketogenic diet was introduced at 3.5 years of age and well tolerated, with perceived slowing of disease progression. Cognitive development was normal; however, there was continued slow deterioration of motor function over the following years, with increasing myopathic stiffness to the point of a loss of the majority of his motor skills. He died at 13 years of age. Trio WGS identified compound heterozygous variants in *NDUFA5* (Figure 1B): a maternally inherited start-loss variant c.1A>C (p.0?) and a paternally inherited synonymous variant c.183G>A (p.Ala61=) located at the splice donor site of exon 3 (Table 1). Despite this, *in silico* tools did not predict a high probability of a mis-splicing event (SpliceAI acceptor loss = 0.12, donor loss = 0.06). Both variants are ultra-rare in gnomAD v.4.1.0, with no reported homozygotes.

**Table 2:** Respiratory chain enzyme activities in isolated mitochondria from cultured skin fibroblasts and skeletal muscle homogenates of individuals F2:II-1 and F3:II-2

	**CS**^a^	**CI/CS**	**CII/CS**	**CIII/CS**	**CIV/CS**	**CV/CS**
F2:II-1						
Fibroblasts						
F2:II-1	204 (113%)	**0**.**10** (**34%**)	0.42 (127%)	0.71 (118%)	0.88 (117%)	0.13 (65%)
Range (controls)	134–228	0.19–0.46	0.17–0.52	0.35–0.87	0.42–1.11	0.12–0.38
Controls (*n* = 22)	181 ± 29	0.29 ± 0.06	0.33 ± 0.09	0.60 ± 0.15	0.75 ± 0.18	0.20 ± 0.08
Skeletal muscle						
F2:II-1	181 (172%)	**0**.**02** (**10**.**5%**)	0.22 (105%)	0.61 (78%)	0.95 (82%)	0.50 (128%)
Range (controls)	70–173	0.14–0.28	0.14–0.36	0.50–1.11	0.57–1.76	0.17–0.66
Controls (*n* = 26)	105 ± 25	0.19 ± 0.04	0.21 ± 0.05	0.78 ± 0.15	1.16 ± 0.28	0.39 ± 0.13
F3:II-2						
Skeletal muscle						
F3:II-2	199 (133%)	**0**.**05** (**36%**)	0.26 (144%)	2.54 (17%)	2.10 (231%)	0.50 (119%)
Range (controls)	150–338	0.14–0.35	0.18–0.41	1.45–3.76	0.91–2.24	0.42–1.26

Values in parentheses present the activities as a percentage of the lowest value of control range. OXPHOS complex measurements in complex I in F2:II-1- and F3:II-2-derived fibroblasts and skeletal muscle are consistent with an isolated complex I deficiency when indicated in bold.

a CS activity (mU/mg homogenate protein).

Family 3 includes a female proband (F3:II-2) born to second-cousin consanguineous parents of Turkish ancestry (Figure 1A). Prenatal and perinatal development were unremarkable. At 10 months of age, she experienced a febrile seizure with a normal EEG recorded. She presented at 2.5 years with nystagmus, bilateral optic atrophy, and gait instability (Table 1). A markedly elevated lactate level (6.1 mmol/L) raised suspicion for mitochondrial dysfunction. By the age of 3 years, a brain MRI was performed that revealed two oval-shaped hyperintensities in the substantia nigra along with a subtle band-like signal increase in the periaqueductal region (Figure 1D). These results, together with respiratory chain enzymology on skeletal muscle biopsy that revealed an isolated complex I deficiency (Table 2), confirmed a mitochondriopathy. F3:II-2 showed continued neurological decline and by 18 years was no longer able to walk independently, requiring both a wheelchair and a rollator. Notably, no neutropenia or cardiac phenotypes were observed for F3:II-2. Singleton exome sequencing identified the same synonymous *NDUFA5* c.183G>A (p.Ala61=) variant found in F2:II-1 (Figure 1B and Table 1).

**Figure 2:**
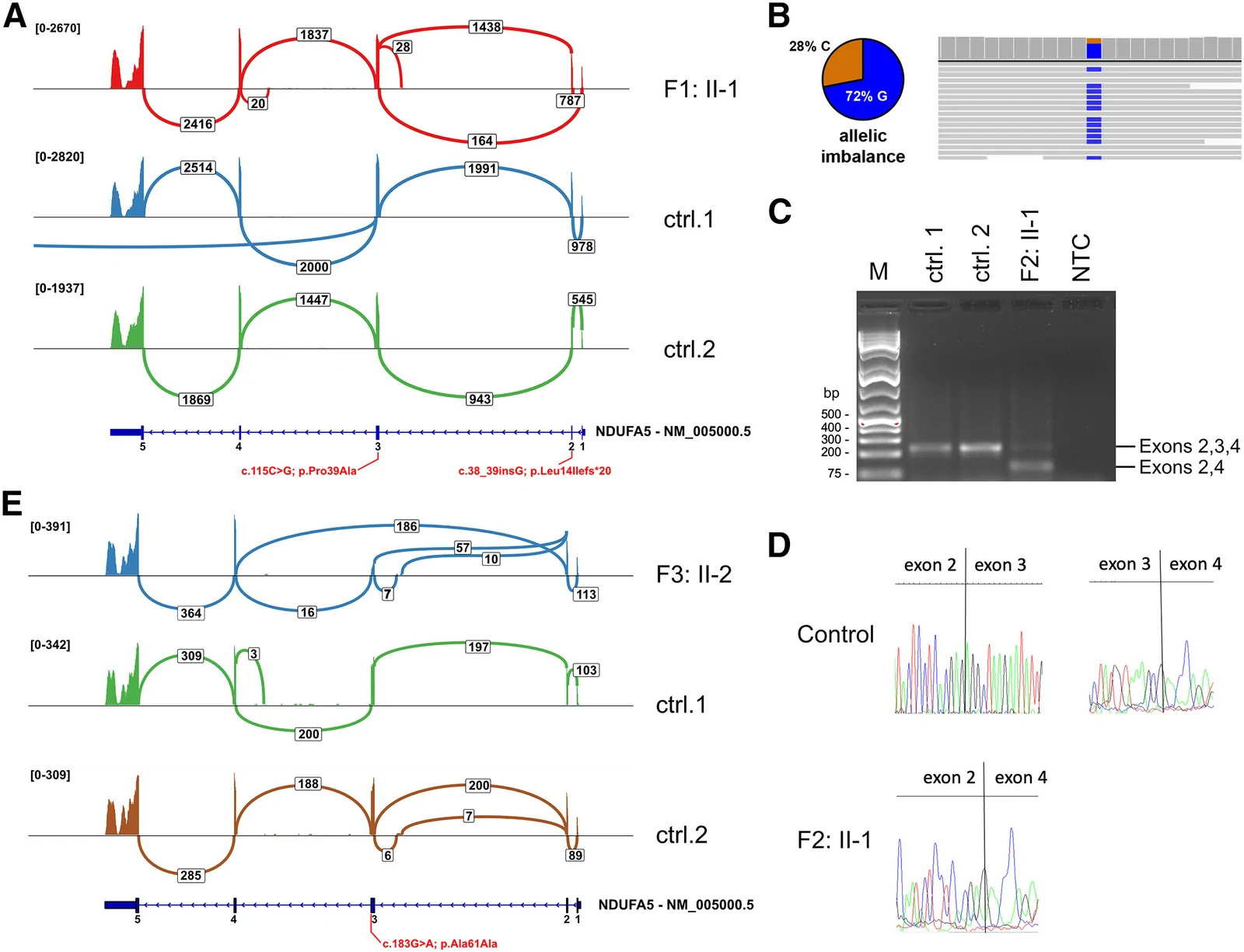
RNA studies (A) Sashimi plot visualization of RNA-seq in F1:II-1 (top) and two control individuals (middle and bottom). The *NDUFA5* frameshift variant, c.38_39insG, results in two distinct outcomes for this allele: the introduction of a premature termination codon, which predisposes to nonsense-mediated mRNA decay for transcripts retaining exon 2, and the skipping of exon 2, which leads to an in-frame deletion of 15 amino acids. (B) Allelic imbalance for F1:II-1, showing allele balance bias toward the missense variant c.115C>G (p.Pro39Ala) located in exon 3. (C) Transcript cDNA analysis of *NDUFA5* in F2:II-1 and two controls. Individual F2:II-1 has an additional *NDUFA5* transcript species compared to the control samples. NTC, no template control. (D) Sequence analysis of *NDUFA5* cDNA from F2:II-1 shows aberrant splicing due to the c.183G>A variant by skipping of exon 3 (shown are the exon-exon junctions). (E) Sashimi plot visualization of RNA-seq in F3:II-2 (top) and two control individuals (middle and bottom). The *NDUFA5* splice variant, c.183G>A, leads to skipping of exon 3 in the majority of reads.

To investigate the functional consequence of these *NDUFA5* variants, we first analyzed their transcriptomic impact. The frameshift variant c.38_39insG (p.Leu14Ilefs^∗^20) in family 1 siblings F1:II-1 and F1:II-2 occurs in exon 2 and is predicted to introduce a premature termination codon. RNA-seq of a lymphoblastoid cell line (LCL) derived from F1:II-1 suggests that the frameshift variant undergoes nonsense-mediated mRNA decay (NMD), with the frameshift variant detected in approximately 10% of reads indicating a loss of transcripts from the frameshift allele. However, this skewing is an under-representation, as the RNA-seq data also reveal the skipping of exon 2, within which the frameshift variant is located, occurring in approximately 17% of reads (Figure 2A). Exon 2 is a 15-amino-acid in-frame coding exon, the skipping of which would not be predicted to induce NMD. Of note, there is an annotated transcript of *NDUFA5* (GenBank: NM_001282419.3), which does not utilize exon 2, thus supporting the existence of natural isoforms that tolerate the exclusion of exon 2. While *in silico* splice predictions for this frameshift variant are not highly suggestive of a mis-splicing outcome (SpliceAI Δ = 0.04 accepter loss, Δ = 0.02 donor loss), the detection of exon 2 skipping in F1:II-1 may reflect enhancement of a non-canonical splicing phenomenon that is occurring at low frequency. SpliceAI confirms that this exon 2 skipping event occurs at low frequency in controls with a GTEx lymphocyte per-sample average read count of 3. Notably, the downstream missense variant in family 1 siblings, c.115C>G (p.Pro39Ala), shows allele balance bias with approximately 72% of transcripts retaining the missense variant (Figure 2B), which is also supportive of a loss of transcripts from the frameshift allele. Collectively, these data are concordant with two distinct consequences of the in *trans* frameshift allele: nonsense-mediated decay of transcripts retaining exon 2 and increased exon 2 skipping leading to the in-frame deletion of 15 amino acids.

Family 2 proband, F2:II-1, was compound heterozygous for a start-loss c.1A>C (p.0?) variant and a synonymous c.183G>A variant that impacts upon the splice donor junction of exon 3. The same synonymous variant is homozygous in family 3 proband F3:II-2 (Figure 1A). While the *in silico* prediction tool SpliceAI does not predict a significant mis-splicing outcome (predicted splice donor loss score: 0.02), transcript analysis of cDNA from F3:II-2-derived fibroblasts revealed an additional smaller product in F2:II-1 (Figure 2C), which was confirmed by Sanger sequencing (Figure 2D) to lack exon 3. This splice defect was confirmed and quantified by RNA-seq on fibroblasts from F3:II-2. RNA-seq analysis of F3:II-2, who is homozygous for the c.183G>A variant, revealed exon 3 skipping in the majority of reads with only 10%–20% correctly spliced transcripts (Figure 2E), implicating the c.183G>A variant as causative. Importantly, skipping of exon 3 does not lead to a frameshift event but rather an in-frame deletion of 39 amino acids, which corresponds to approximately one-third of the 116-amino-acid NDUFA5 and therefore should be interpreted as p.Arg23_Ala61del.

**Figure 3:**
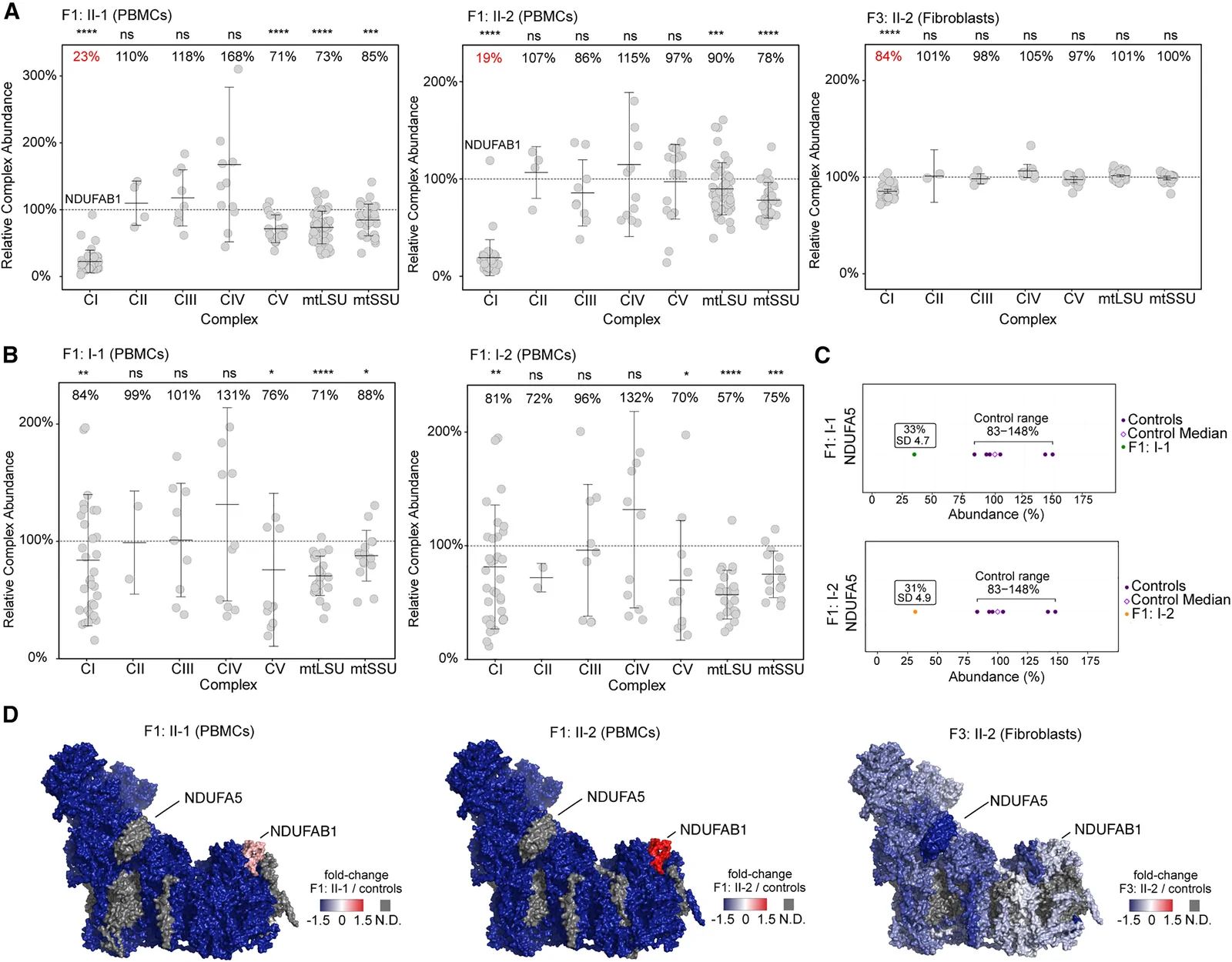
Proteomic studies (A) Relative complex abundance (RCA) of oxidative phosphorylation (OXPHOS) complexes and mitoribosome subunits of peripheral blood mononuclear cells (PBMCs) from family 1 proband (F1:II-1) and affected sibling (F1:II-2) and fibroblasts from family 3 proband (F3:II-2). Middle bar represents mean complex abundance. Upper and lower bars represent 95% confidence interval. Significance was calculated from a two-sided *t* test between the individual protein means. ^∗∗∗∗^*p* < 0.0001, ^∗∗∗^*p* < 0.001, ^∗∗^*p* < 0.01, ^∗^*p* < 0.05; ns, not significant (*p* > 0.05). CI–CV, OXPHOS complexes I–V; mtLSU, mitoribosome large subunit; mtSSU, mitoribosome small subunit. (B) RCA of PBMCs from family 1 carrier parents (F1:I-1 and F1:I-2). Middle bar represents mean complex abundance. Upper and lower bars represent 95% confidence interval. Significance was calculated from a two-sided *t* test between the individual protein means. ^∗∗∗∗^*p* < 0.0001, ^∗∗∗^*p* < 0.001, ^∗∗^*p* < 0.01, ^∗^*p* < 0.05; ns, not significant (*p* > 0.05). CI–CV, OXPHOS complexes I–V; mtLSU, mitoribosome large subunit; mtSSU, mitoribosome small subunit. (C) Abundance range of NDUFA5 protein in PBMCs from F1:I-1 and F1:I-2 (carriers) relative to the median (purple diamond) of six individual adult controls (*n* = 6, purple dots). SD, standard deviation. (D) Topographical heatmap of the fold-change values of complex I subunits in probands F1:II-1, F1:II-2, and F3:II-2 relative to controls. PDB: 5LDW. N.D., not detected (shown as gray in images).

To investigate the impact of the missense variant c.115C>G (p.Pro39Ala) variant identified in family 1, whole-cell proteomics was performed on quad peripheral blood mononuclear cells (PBMCs) from F1:II-1 and F1:II-2 carrying a maternally inherited p.Pro39Ala in *trans* with a paternally inherited c.38_39insG (p.Leu14Ilefs^∗^20) and their unaffected carrier parents (Figure 1A). Proteomics-based relative complex abundance (RCA) analysis showed a significant and isolated reduction in the abundance of complex I to 23% and 19% in affected siblings F1:II-1 and F1:II-2, respectively, relative to pediatric controls (Figure 3A). NDUFA5 was not detected in PBMCs from the affected siblings but was well detected (3–5 peptides) in age-matched controls. As a consequence, both family 1 siblings meet the threshold for a major defect by RCA (≤65% relative to controls or ≤75% with absent detection of the protein of interest in the proband with >2 peptides detected in controls) defined by Hock et al.^15^ Parental PBMC samples for family 1 showed a complex I RCA abundance of 84% in the father (F1:I-1) and 81% in the mother (F1:I-2) (Figure 3B), with NDUFA5 protein detected at 33% control median in the father (F1:I-1) and 31% in the mother (F1:I-2) (Figure 3C). Importantly, NDUFA5 levels were outside of the control range (83%–148% control median) in both parents, suggesting that the maternally inherited missense p.Pro39Ala variant has a similar impact on NDUFA5 abundance as the paternally inherited frameshift variant that undergoes NMD (Figure 2A). Topographical heatmap of the fold changes of complex I subunits in probands from family 1 also showed reduced abundance of all subunits across the complex (Figure 3D) except for NDUFAB1, which has a dual localization with members of the Leu-Tyr-Arg motif (LYRM) protein family in the mitochondrial matrix.^16^

Proteomics was also performed on fibroblasts from the proband of family 3 (F3:II-2) who is homozygous for the c.183G>A (p.Arg23_Ala61del) variant, which results in an in-frame deletion of 39 amino acids that escapes NMD. Complex I abundance in F3:II-2-derived fibroblasts was 84% control median (Figure 3A), impacting subunits from across the complex (Figure 3D). While this result does not meet the criteria for a major or minor RCA defect,^15^ it is important to note that we analyzed an unaffected tissue, fibroblast cells, where canonical splicing was observed in ∼25% of reads (Figure 2E). This is consistent with the complex I defect in individual F2:II-1 being more pronounced in muscle than in fibroblast cells (Table 2).

**Figure 4:**
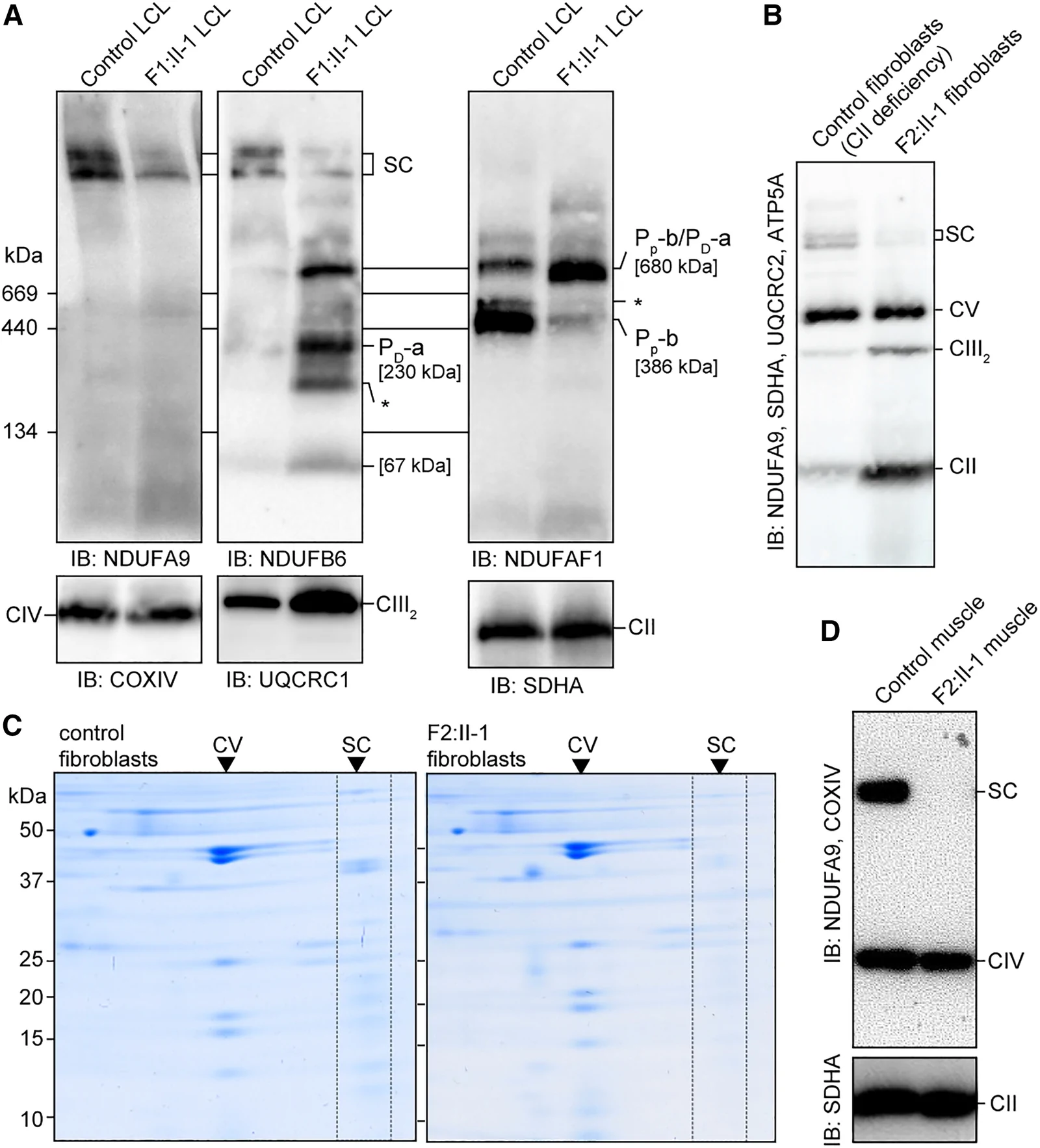
Complex I assembly studies (A) BN-PAGE and immunoblotting (IB) on mitochondria from control and F1:II-1 lymphoblastoid cell lines (LCLs) using the indicated antibodies. Subcomplex assignment is based on the published nomenclature, which includes reference to molecular weights as first reported.^17^ Asterisk (^∗^) indicates non-specific or assigned band. CII, complex II; CIII, complex III; CIV, complex IV. (B) As for (A) but utilizing a cocktail of antibodies for CI (NDUFA9), CII (SDHA), CIII (UQCRC2), and CV (ATP5A) on F2:II-1 and control (CII deficiency) fibroblast mitochondria. (C) Comparison of the OXPHOS proteome via 2D BN-PAGE of F2:II-1 and control fibroblast mitochondria. (D) As for (A) but utilizing the indicated antibodies to decorate CI containing SCs, CIV (COXIV), and CII.

To investigate the impact on complex I and respiratory chain SC assembly, LCLs, fibroblasts, and skeletal muscle homogenates from the probands of family 1 (F1:II-1) and family 2 (F2:II-1) were analyzed by blue native (BN)-PAGE. As can be seen in Figure 4A, BN-PAGE and immunoblotting with antibodies against NDUFA9 (localized in the peripheral complex I Q module^17^) and NDUFB6 (complex I membrane arm P_D_ module, itself composed of P_D_-a and P_D_-b, also known as the ND4 and ND5 module, respectively^3^) identified two complex-I-containing SC assemblies that were less abundant in F1:II-1 LCLs compared to control. A similar reduction in SCs was seen in F2:II-1 fibroblasts compared to a complex II disease control (Figure 4B) and additionally resolved using 2D BN-PAGE (BN-PAGE followed by SDS-PAGE) (Figure 4C). Complex III (the free complex III dimer, CIII_2_) was increased in abundance in both probands (Figures 4A and 4B), likely due to the reduced amount of SC assembled complex I compared to controls. Interestingly, complex I and the SC was completely absent in F2:II-1 skeletal muscle homogenate, while free complex IV and complex V were unchanged (Figure 4D). These findings concord with the biochemical measurements of reduced complex I activity (Table 2) with a more pronounced effect seen in F2:II-1’s skeletal muscle compared to fibroblasts. When considering that the start-loss variant in F2:II-1 is most likely to produce no protein, these findings likely represent the sole impact of the in-frame exon 3 skipping variant, c.183G>A, and are consistent with the broad reduction in complex I subunits seen in fibroblasts from F3:II-2 (Figure 3D) who is homozygous for the same c.183G>A variant. We next asked which step of complex I biogenesis is impacted in F1:II-1 mitochondria. Complex I assembles through a stepwise coalescence of mtDNA encoded subunits (ND1–7 and ND4L) with discrete modules of nuclear encoded core and accessory subunits.^3^^,^^17^^,^^18^ NDUFA5 assembles with other Q-module subunits—NDUFS2, NDUFS3, NDUFS7, and NDUFS8—as a subcomplex, which joins the mtDNA encoded ND1 and several accessory subunits—NDUFA3, NDUFA8, and NDUFA13—to form the Q/P_p_-a (Q/ND1^3^) intermediate.^17^ In parallel, an intermediate containing ND2, ND3, ND4L, and ND6 and accessory subunits NDUFC1 and NDUFC2 is formed through the action of the mitochondrial complex I intermediate assembly (MCIA) assembly factors, resulting in a stable 386 kDa P_p_-b (also known as the ND2 module when not associated with MCIA^3^) assembly that is readily resolved on BN-PAGE using antibodies against NDUFAF1 (Figure 4A, top right).^17^^,^^18^ Formation of this intermediate is strongly inhibited in F1:II-1 LCLs compared to control. Moreover, the 230 kDa P_D_-a intermediate—ND4, NDUFB1, NDUFB5, NDUFB6, NDUFB10, and NDUFB11, a 67 kDa pre-P_D_-a assembly lacking ND4—and the 680 kDa P_p_-b/P_D_-a intermediate^17^ strongly accumulate in F1:II-1 LCLs (Figure 4A). Taken together, these results suggest that, in the absence of NDUFA5, assembly of complex I is blocked at the penultimate step of biogenesis where Q/P_p_-a and P_p_-b/P_D_-a join to form the Q/P intermediate (complex I lacking the N module).^17^

**Figure 5:**
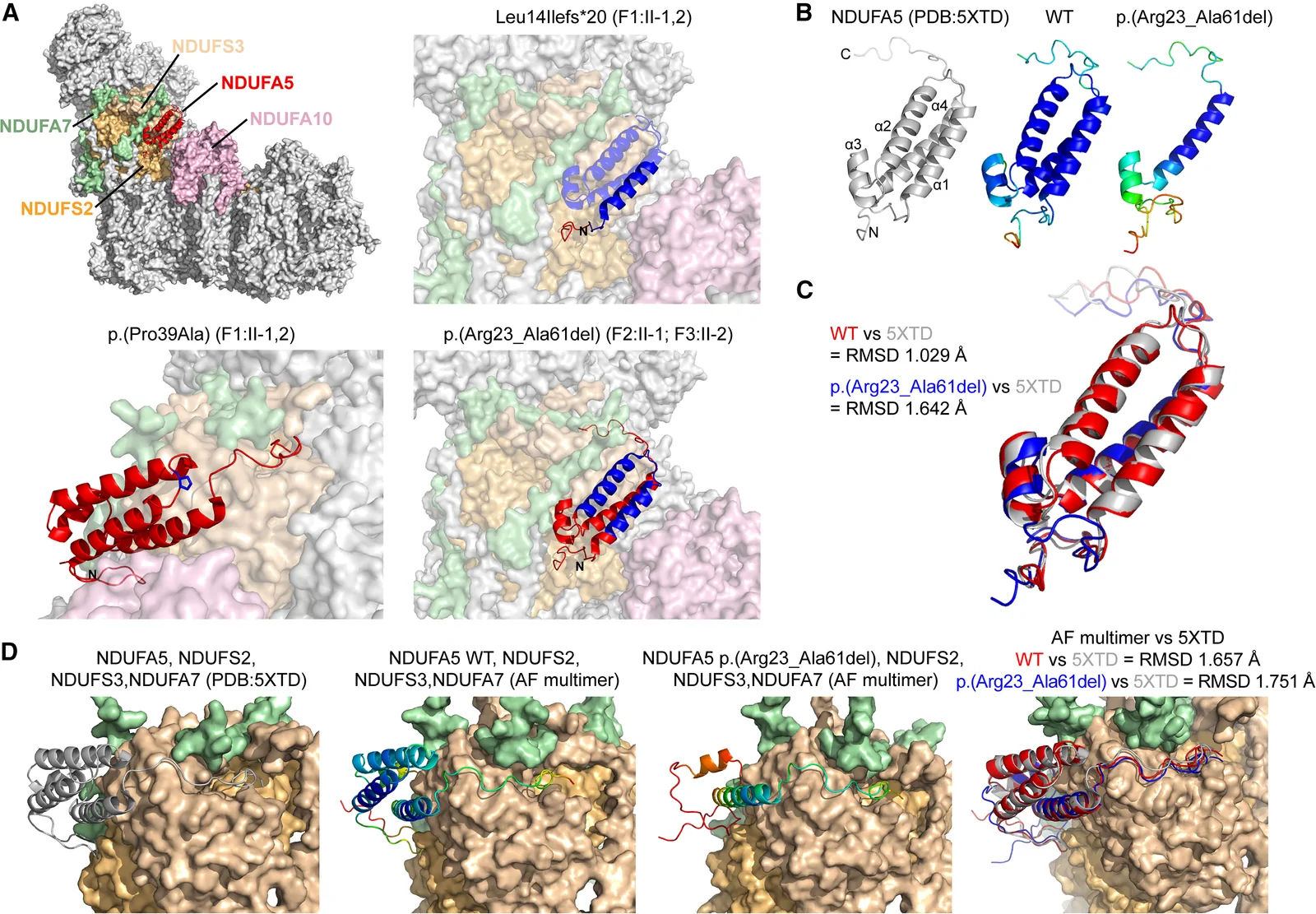
Molecular modeling (A) Cryo-EM structure of the human complex I^7^ (PDB: 5XTD) with NDUFA5 rendered in cartoon format. The blue regions of NDUFA5 in the subpanels indicate the deleted or substituted residues. (B) Left: cartoon representation of human NDUFA5^7^ with the alpha helices (α) numbered as indicated. Middle and right: AlphaFold2^20^ models of WT and Arg23_Ala61del NDUFA5 colored according to pLDDT (red, low confidence; blue, high confidence). (C) Alignment of structures in (B) using the *super* algorithm with the RMSD of WT (red) and Arg23_Ala61del (blue) NDUFA5 aligned to the experimentally observed protein (gray) as indicated. (D) Left: experimentally determined^7^ structure (extracted from PDB: 5XTD) for the subcomplex of NDUFA5 (gray), NDUFS2, NDUFS3, and NDUFA7 (subunits are colored as in A). Middle: AlphaFold-multimer^21^ models of subcomplex containing WT or Arg23_Ala61del NDUFA5 colored according to pLDDT. Right: RMSD of the WT (red) or Arg23_Ala61del (blue) containing subcomplexes aligned to the experimentally determined structure.

We also investigated the potential impact of each variant on NDUFA5 protein and its interactors. Figure 5A shows the location of NDUFA5 in the cryogenic electron microscopy (cryo-EM) structure of human complex I.^19^ NDUFA5 makes protein-protein interactions with Q-module subunits NDUFS2 and NDUFS3 as well as accessory subunits NDUFA7 and NDUFA10.^4^^,^^19^ These interactions occur through the N-terminal region of NDUFA5 with NDUFS2, α helix 1 with NDUFA10, α helices 2 and 4 with NDUFS3, and the C-terminal region with NDUFS2, NDUFS3, and NDUFA7. The nonsense variant p.Leu14Ilefs^∗^20 in the family 1 siblings results in loss of α helices 2–4 and all surfaces that interact with NDUFS3, the C-terminal region interacting with NDUFS2 and NDUFA7 at the surface of complex I, and the buried C terminus that interacts with NDUFS3 (Figure 5A, top right). Previous studies have shown a high co-dependence between NDUFA5 and NDUFS3, with the partner subunit also degraded in fibroblasts from individuals with *NDUFS2* variants^15^ and a HEK293T *NDUFA5* knockout model^3^ leading to a severe complex I assembly defect stalled with assembly of the P_D_-a and P_D_-b (ND4 and ND5) modules. Given that NDUFA5 was not detected in PBMCs from both F1:II-1 and F1:II-2 (Figure 3D), it is likely that subsequent destabilization of NDUFS3 is deleterious to assembly of the Q module. A similar conclusion can be made for the p.Pro39Ala variant found compound with the nonsense variant in family 1, as removal of the Pro39 residue likely results in destabilization of the α1 helix-turn-α2 helix motif (Figure 5A, bottom left).

F2:II-1 and F3:II-2 share the in-frame deletion variant, p.Arg23_Ala61del, with F2:II-1 being compound heterozygous with the start loss c.1A>C variant, while F3:II-2 is homozygous for the in-frame deletion variant (Figure 1A). The p.Arg23_Ala61del variant removes most of α helices 1 and 2 (Figure 5A, bottom right). Given there is no co-dependency between NDUFA10 and NDUFA5 in HEK293T knockout models,^3^ we suppose that loss of this interaction is not deleterious. However, p.Arg23_Ala61del results in loss of the α2-NDUFS2 interaction and potentially the interactions with NDUFS2, NDUFS3, and NDUFA10 through helices 3 and 4 and the proteins at the C terminus. Given that NDUFA5 is produced and present in F3:II-2 (Figure 3D), who is homozygous for p.Arg23_Ala61del and whose clinical presentation is less severe than the other three individuals in this cohort, we supposed that loss of the α1 helix-turn-α2 helix motif in NDUFA5 is tolerated. To test this, we modeled the wild-type (WT) and Arg23_Ala61del sequence of NDUFA5 using ColabFold (AlphaFold2)^20^^,^^22^ with AMBER relaxation enabled to resolve steric clashes. The model with the highest predicted local distance difference test (pLDDT) confidence score was selected for analysis (Figure 5B). The AlphaFold-derived model of WT NDUFA5 was aligned to the experimentally determined structure of the human protein^19^ using the *super* algorithm (structure-based dynamic programming alignment) yielding a root-mean-square deviation (RMSD) of 1.029 Å, demonstrating that AlphaFold can model the structure of NDUFA5 with good accuracy (Figure 5C). Alignment of Arg23_Ala61del NDUFA5 against the experimentally determined structure yielded an RMSD of 1.642 Å, with the core fold including α helices 3 and 4 preserved. To assess the structural stability of NDUFA5 in complex with its interactors, we modeled a complex of NDUFS2, NDUFS3, NDUFA7, and NDUFA5 using AlphaFold-multimer^21^^,^^22^ (Figure 5D). Alignment of the complex containing WT NDUFA5 against the experimentally observed complex^19^ had a core RMSD of 1.657 Å (Figure 5D, right), demonstrating that AlphaFold-multimer can model the Q-module structure with reasonably high accuracy. Crucially, the Arg23_Ala61del-containing model maintained this structural integrity, with a core RMSD of 1.751 Å, indicating that the deletion likely does not perturb the stability of the complex and suggesting that the interactions between the NDUFA5 α4 helix and C terminus with NDUFS2, NDUFS3, and NDUFA7 are retained.

**Figure 6:**
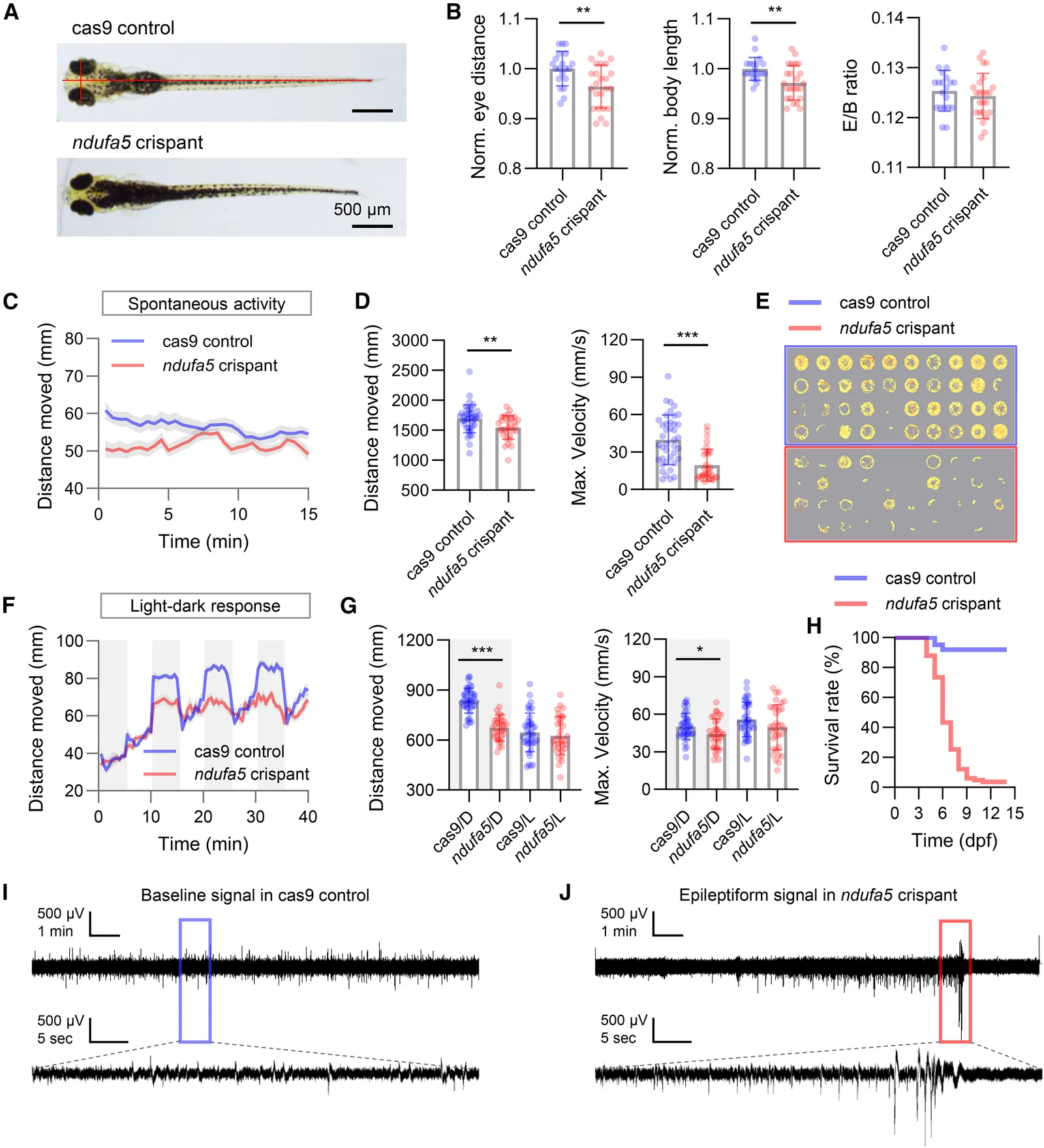
Disruption of zebrafish *ndufa5* results in abnormal embryonic development and neurodevelopmental deficits (A) Representative brightfield images of 5-dpf larval zebrafish (dorsal view). Top: cas9-injected control; bottom: *ndufa5* crispant. Red lines denote eye distance and body length. (B) Quantification of eye distance, body length, and eye distance/body length (E/B) ratio in cas9 controls (*n* = 20 fish) versus *ndufa5* crispants (*n* = 24 fish). Data are normalized to the mean of the cas9 control group. (C) Spontaneous swim activity measured by distance moved for cas9 controls (*n* = 40 fish) and *ndufa5* crispants (*n* = 31 fish). (D) As for (C) but quantification of total distance moved and maximum velocity. (E) As for (C) but locomotion traces over 15 min of recording. (F) Light-dark evoked response measured by distance moved for cas9 controls (*n* = 40 fish) and *ndufa5* crispants (*n* = 33 fish); gray blocks indicate dark periods. (G) As for (F) but quantification of total distance moved and maximum velocity across dark-light epochs. (H) Survival curves for cas9 controls (*n* = 62 fish) and *ndufa5* crispants (*n* = 83 fish). (I) Representative local field potential (LFP) recordings from cas9 controls, showing baseline activity (magnified view below blue box). (J) As for (I) but from *ndufa5* crispants, showing epileptiform events (magnified view below red box). Scale bars are indicated in the figure. Error bars represent SEM for distance moved plots and SD for quantification plots. Statistical significance is indicated as ^∗^*p* < 0.05, ^∗∗^*p* < 0.01, and ^∗∗∗^*p* < 0.001.

Finally, we investigated the function of *ndufa5*, the zebrafish ortholog of human *NDUFA5*, during early development. Using CRISPR-Cas9 genome editing, we generated *ndufa5* F0 mutants (crispants) and evaluated their phenotypic consequences through morphological, behavioral, and electrophysiological assessments at 5–6 days post fertilization (dpf). Compared to Cas9-injected controls, *ndufa5* crispants exhibited notable developmental abnormalities. Morphological analysis revealed a significant reduction in eye distance (Figures 6A and 6B; unpaired *t* test, *p* = 0.0045) and body length (*p* = 0.0037), while the ratio of eye distance to body length remained unchanged (*p* = 0.4431), indicating proportional growth retardation. Additionally, *ndufa5* crispants displayed increased dark pigmentation relative to Cas9 controls. Behavioral analyses of 5-dpf larvae demonstrated significant impairments in spontaneous locomotor activity, with *ndufa5* crispants showing reduced total distance moved and maximum velocity (Figures 6C–6E; unpaired *t* test, *p* = 0.0063 and *p* < 0.0001, respectively). Furthermore, during light-dark stimulus paradigms, *ndufa5* crispants exhibited markedly diminished locomotor responses during dark intervals, as reflected by significantly decreased distance moved (*p* < 0.0001) and maximum velocity (*p* = 0.0249), whereas no significant differences were observed under light conditions (*p* = 0.4650 for distance moved, *p* = 0.1016 for maximum velocity) (Figures 6F and 6G). Survival analysis revealed a significantly reduced survival rate in *ndufa5* crispants compared to Cas9 controls (Figure 6H; log-rank test, *p* < 0.0001), with none of the *ndufa5* crispants surviving beyond 15 dpf and more than half succumbing by 6 dpf. While electrophysiological recordings of Cas9-injected controls (30/30) exhibited only baseline neural activity (Figure 6I), spontaneous epileptiform activity, including ictal-like and interictal-like discharges, were observed in *ndufa5* crispants (5/30) (Figure 6J). Collectively, these findings demonstrate that *ndufa5* is critical for normal embryonic development and neurological function in zebrafish larvae.

Aside from a recent report of a single individual,^23^ variants in *NDUFA5* have not been associated with a human mitochondriopathy. A previous study utilizing TALEN and CRISPR-Cas9 gene-editing tools to disrupt *NDUFA5* in HEK293T cells demonstrated a convincing complex I deficiency via this knockout assay.^3^ As such, there was a robust *a priori* hypothesis that NDUFA5 dysfunction could give rise to a complex I deficiency phenotype in humans.

The most common clinical conditions associated with mitochondrial complex I dysfunction are Leigh syndrome, Leber hereditary optic neuropathy (LHON; MIM: 535000), mitochondrial encephalomyopathy with lactic acidosis and stroke-like episodes (MELAS; MIM: 540000), myoclonic epilepsy with ragged-red fibers (MERRF; MIM: 545000), fatal infantile lactic acidosis, hypertrophic cardiomyopathy, and early-onset neurodegenerative disorders. Individuals frequently present with neurological symptoms such as encephalopathy, seizures, ataxia, dystonia, and psychomotor regression as well as myopathy, exercise intolerance, and lactic acidosis. Multisystem involvement is common, affecting the nervous system, skeletal muscle, heart, liver, and kidneys. Vision loss due to optic nerve degeneration is characteristic of LHON. The clinical spectrum is highly variable, with presentations ranging from isolated organ involvement to severe multisystem disease with early childhood onset and high mortality.^1^^,^^12^^,^^24^^,^^25^^,^^26^^,^^27^^,^^28^ In addition to *NDUFA5*, other nuclear genes encoding non-catalytic subunits of complex I have been identified to be associated with mitochondrial disorders, including Leigh syndrome or isolated optic neuropathy.^10^^,^^11^^,^^28^^,^^29^

The multisystem phenotype seen for each individual in this cohort warranted genomic sequencing as the comprehensive genetic investigation of choice for each family. The most suspicious candidate variants led to the identification of bi-allelic variants in *NDUFA5* and the amalgamation of the three reported families into a single cohort through GeneMatcher.^12^^,^^26^^,^^30^^,^^31^^,^^32^ Each family was ascertained from a different country, with individuals coming to medical attention at different time points throughout the last two decades. As such, genetic and functional testing has been necessarily varied between individuals depending on technological accessibility in their country of origin. The testing centers for each of the three families independently confirmed a complex I deficiency in all affected individuals, incorporating the detection of DNA variants, aberrant RNA phenotypes, and reduced mitochondrial complex I proteins in three different tissues. In an additional model, we show that NDUFA5 deficiency in zebrafish impairs embryonic and neural development. In combination, we provide convincing evidence that *NDUFA5* deficiency results in a multisystemic mitochondrial disorder.

Infants and young children are most frequently affected by mitochondrial complex I dysfunction. The majority of cases present before the age of 3 years, with onset in infancy being typical for the most severe phenotypes, including Leigh syndrome and fatal infantile lactic acidosis.^12^^,^^26^^,^^30^^,^^31^^,^^32^ Adolescents and adults can also be affected, but this is less common and usually associated with specific syndromes such as LHON, which most often presents in young adults (median age of onset around 24 years), and occasionally with MELAS or MERRF, which may have variable age of onset but are still more common in childhood or adolescence.^1^^,^^33^ Congenital heart disease is rare in mitochondrial disorders but has been reported in other individuals with complex I deficiency and other mitochondrial disorders.^34^ As the congenital cardiac malformations seen for the affected siblings in family 1, namely coarctation of the aorta and atrial septal defects, are not well-recognized features of mitochondrial conditions, specific attention was paid to the analysis of virtual gene panels covering aortopathy conditions and congenital heart disease, neither search of which identified variants of clinical significance. It should be noted that the cardiac arrhythmia for F1:II-1 and cardiac hypertrophy for F1:II-2 are phenotypes appreciated in mitochondrial-related cardiac disease. Furthermore, these siblings from family 1 are the only individuals in this small cohort to present with congenital hematological abnormalities, a variable manifestation that is seen across many primary mitochondrial disorders.^35^^,^^36^ Cytogenetic and flow-cytometry testing of a bone marrow sample from F1:II-1 did not detect any underlying primary hematological malignancy or other molecular cause for the various cytopenias, which is in support of these hematological abnormalities being part of the unifying mitochondrial complex I deficiency. As such, these cardiac and hematological aspects of the siblings’ phenotype may represent a higher degree of multisystem variability over that of the other two individuals in this cohort. Certainly, the identification of a larger number of individuals with bi-allelic variants in *NDUFA5* resulting in complex I deficiency is required to better understand the phenotypic spectrum of *NDUFA5* dysfunction.

Interestingly, *NDUFA5*gene is neither missense constrained (*Z* score 0.85; observed/expected [o/e] 0.76, range 0.62–0.93), nor predicted loss-of-function (pLoF) constrained (probability of LoF intolerance [pLI] 0; o/e 0.78, range 0.46–1.41) as per gnomAD v.4.1.0. While all four variants reported for this cohort are present in the various gnomAD datasets, all have variant allele frequencies that could be considered acceptable for carrier rates relating to a recessive monogenic condition. Further to this, none are present in a homozygous state. Significantly, there are no compound heterozygous or homozygous rare variant pairings for *NDUFA5* across all variant classes as per gnomAD v.2.1.1 variant co-occurrence data. Our zebrafish studies show that *ndufa5* is critical for normal embryonic development and is consistent with a reported embryonic lethality in *Ndufa5* knockout mice.^1^ Taken together, it is a reasonable supposition that bi-allelic LoF variants in *NDUFA5* may also be incompatible with embryonic survival in humans. This is supported by the various bi-allelic variant pairings seen for our cohort, where no combination is predicted to result in complete ablation of NDUFA5 protein production but, rather, the prediction of generating aberrant protein products in each instance.

In conclusion, we provide convincing clinical and functional genomic evidence, together with a substantive animal model and molecular modeling, in support of a mitochondrial complex I deficiency syndrome due to bi-allelic variants in *NDUFA5*, thus adding to the molecular differential diagnoses for complex I mitochondriopathies. For those individuals in whom a complex I deficiency has been clinically suspected and/or biochemically confirmed, but a molecular diagnosis remains elusive, this report serves to encourage the reanalysis of existing data regarding the potential for an *NDUFA5*-related mitochondriopathy. The cohort reported herein also exemplifies the complex pathomechanisms that may be encountered in diagnostic genomics and the potential need for adjunctive transcriptomics and proteomics in service of variant pathogenicity to secure a molecular diagnosis.

## Data and code availability

The raw genomic and proteomic data that support the findings of this study have not been deposited in a public repository due to human research ethics committee and institutional review board restrictions but are available, along with all other materials, from the corresponding authors on request. This project did not involve development of new code.

## Acknowledgments

The authors acknowledge and thank the families who participated in this study. The biochemical analyses for family 2, including respiratory chain enzymology measurements and protein studies, were performed by our much-respected colleague Dr. Dagmar Hahn, who sadly passed away in 2016. This project was supported by grants from the 10.13039/501100000925National Health and Medical Research Council (NHMRC) to N.B.T. (GNT2005458 and GNT1079342) and D.A.S. (GNT2009732); the 10.13039/501100025520Medical Research Future Fund (MRFF) to D.A.S., D.R.T., and D.H.H. (MRF2016030) and D.A.S. and D.H.H. (NCRI000043); Rare Disease Now (RDNow) and Undiagnosed Diseases Program Victoria (UDP-Vic) to S.M.W.; BMFTR (German Federal Ministry of Research, Technology, and Space) through the German Center for Child and Adolescent Health (DZKJ), the German Network for Mitochondrial Disorders (mitoNET, 01GM1906A), and EJP RD project GENOMIT (01GM1920A and 01GM2404A) to H.P., T.K., and R.K.; and the European Union’s 10.13039/501100007601Horizon 2020 research and innovation program project Recon4IMD (101080997) to H.P. T.K. is a member of the European Reference Network (ERN) for Rare Neurological Diseases (ERN-RND) and the ERN for Neuromuscular Diseases (EURO-NMD). The research conducted at the 10.13039/100014555Murdoch Children’s Research Institute (MCRI) was supported by the Victorian Government’s Operational Infrastructure Support Program. The project involved the Rare Disease Flagship, which acknowledges financial support from the 10.13039/100014607Royal Children’s Hospital Foundation, the 10.13039/100014555MCRI, Harbig Foundation, Paula Fox, Andrew and Geraldine Buxton Foundation, and 10.13039/501100022893The Pierce Armstrong Foundation. We thank the Mito Foundation for equipment grants to D.A.S. and D.H.H. and the Bio21 Mass Spectrometry and Proteomics Facility (MMSPF) for the provision of instrumentation, training, and technical support.

## Author contributions

Conceptualization, N.B.T., T.B., A.S., J.L., D.A.S., and H.P.; clinical assessment, N.B.T., M.C., M.R., M.G., T.B., M.W., T.K., and W.M.-F.; investigation, D.H.H., T.E.G., T. Sharma, K.M.B., T. Stait, R.K., A.S., J.L., J.W., and X.Q.; funding acquisition, N.B.T., S.M.W., D.H.H., D.A.S., and H.P.; supervision, S.M.W., D.R.T., J.L., K.M.B., D.A.S., and H.P.; writing – original draft, N.B.T., M.R., and D.A.S.; writing – review & editing, all authors.

## Declaration of interests

Cipher Gene Chengdu Ltd. is a medical data development company that employs J.W., X.Q., and J.L. T.K. has received research support, speaker honoraria, consulting fees, and travel reimbursement from Santhera Pharmaceuticals, Chiesi GmbH, and GenSight Biologics.

## Web resources

GenBank, https://www.ncbi.nlm.nih.gov/genbank/
gnomAD, https://gnomad.broadinstitute.org/
OMIM, https://www.omim.org/

## Supplemental information

Document S1. Supplemental notes: Case reports

Document S2. Article plus supplemental information
