# Supplementary material for: Bi-allelic variants in NDUFA5 cause a mitochondriopathy with complex I deficiency
Source: Am J Hum Genet. 2026 Mar 30;113(5):1108–21. doi: 10.1016/j.ajhg.2026.03.003 (PMC13277682; doi:10.1016/j.ajhg.2026.03.003)
Supplement: Document S1. Supplemental notes: Case reports [file mmc1.pdf]

The American Journal of Human Genetics, Volume *113*

Supplemental information

Bi-allelic variants in *NDUFA5* cause a mitochondriopathy with complex I deficiency

Natalie B. Tan, Matthias Gautschi, Michael Raum, Daniella H. Hock, Robert Kopajtich, Jia Wang, Xiao Qian, Tanavi Sharma, Timothy E. Green, Jean-Marc Nuoffer, Katrina M. Bell, Katarzyna Pospieszny, Tegan Stait, Chloe Pike, Michelle Cao, Susan M. White, David R. Thorburn, Theresa Brunet, Matias Wagner, Wolfgang Müller-Felber, Leopold Zeng, Thomas Klopstock, André Schaller, Jing Liu, David A. Stroud, and Holger Prokisch

Supplemental notes: Case Reports

Family 1

Individual F1:II-1 is the first-born child to non-consanguineous parents of Korean descent. Both parents are generally healthy and well, with no known family history of underlying monogenic conditions. Combined first trimester screening returned a low-risk result for the common aneuploidies of trisomy 21, 13 and 18. Morphology ultrasound at 20-weeks' gestation detected a cardiac anomaly that was investigated via dedicated fetal echocardiogram at 21+2 weeks' gestation, demonstrating a hypoplastic aortic arch with left to right heart size discrepancy. Concerns for prenatal growth restriction were monitored via fortnightly growth scans from 32-weeks' gestation, with an estimated fetal weight on the second centile reported by 36-weeks. Amniocentesis for five probe FISH (13, 18, 21, X and Y) and SNP microarray both returned normal results. F1:II-1was born at 36+6 weeks' gestation via a planned Cesarean section owing to the cardiac anomalies and anticipated need for tertiary neonatal cardiac care. He was born in good condition, requiring no resuscitation, with a birth weight of 2120 grams. Postnatal echocardiogram confirmed a coarctation of the aorta with atrial septal defect for which surgical repair was conducted on day 8 of life. Significantly, F1:II-1's post-operative cardiac recovery was complicated by sinus node dysfunction that manifested with a chaotic atrial rhythm that has self-resolved. He was re-admitted to hospital at 4 months of age with increased respiratory effort, worsening feeds and poor weight gain due to persistent pulmonary arterial hypertension secondary to elevated biventricular end diastolic pressures.

Hyperlactatemia, hyperglycemia and hypocalcemia were noted at birth, with the latter two issues resolving quickly with routine management. The hyperlactatemia persisted for the first six months of life. Alongside the antenatal diagnoses of coarctation of the aorta and intrauterine growth restriction, F1:II-1 had persisting postnatal growth restriction with head circumference preservation, hypospadias for which surgery is planned, a sacral dimple with no associated hair tufting and normal spinal ultrasound, dysmorphism that included a large anterior fontanelle with metopic ridging, and congenital generalized hirsutism with merging of the temporal hairline and lateral margins of the eyebrows. The hirsutism resolved across the first months of life. F1:II-1 also has a striking hematological phenotype, with pancytopenia (anemia, thrombocytopenia, leukopenia) detected on the initial full blood count taken at birth. He had a congenital red cell macrocytosis that required multiple red cell transfusions, the first of which occurred on day 12 of life. He continues to experience a persistent but fluctuating neutropenia and lymphopenia. Owing to the clinical suspicion for an underlying bone marrow failure condition, testing of a bone marrow sample was conducted via G-banded karyotype, FISH and flow cytometry, all of which returned uninformative results. The examination of the bone marrow aspirate sample identified the presence of ring sideroblasts and

vacuolated erythroid precursors, which can be seen in mitochondrial disorders. Whilst early developmental concerns were raised in the context of recovery from a complicated neonatal course, F1:II-1 has demonstrated gradual catch-up to largely age-appropriate milestones by age 2 years.

Owing to the complex constellation of medical issues for F1:II-1 at birth, he was investigated for the possibility of an underlying metabolic condition, returning normal results for urine metabolic screening and urinary organic acids, plasma amino acids, free and total carnitine, and chromosome breakage studies. Brain MRI at three weeks of age revealed mildly prominent ventricles only. Clinical trio genome sequencing was requested for F1:II-1 at 15 days of age which was uninformative.

Individual F1:II-2 is the second child born to the same Korean parents. Similar to F1:II-1, she was diagnosed antenatally with congenital heart disease involving a tortuous aortic arch, mildly small left sided structures, and mild biventricular hypertrophy with preserved systolic function, with postnatal echocardiogram also confirming a bicuspid aortic valve, small fenestrated atrial septal defect, and mild left pulmonary artery stenosis; all of which have been conservatively managed. She was also born at 36+6 weeks' gestation via planned Cesarean section in the same context of intrauterine growth restriction that persisted to postnatal growth restriction. Once again, much like her brother, she had neonatal lactatemia and congenital hirsutism that spontaneously resolved, as well as a sacral dimple with normal spinal ultrasound. F1:II-2 has had normal development to age 6 months at the time of last assessment. The hematological phenotype for F1:II-2 mimics a very similar course to that of her brother, with a congenital macrocytic anemia requiring red cell transfusion at 1 month of life, together with persistent neutropenia and lymphopenia.

Given the striking phenotypic overlap between siblings, clinical singleton genome sequencing was also arranged for F1:II-2 shortly after birth and unsurprisingly returned an uninformative result when analyzed together with her parents' existing genome sequencing data. The family was subsequently enrolled into a rare disease research program where quad WGS analysis identified compound heterozygous variants of interest in *NDUFA5* that were shared by these siblings. The maternally inherited missense variant, p.(Pro39Ala), affects a highly conserved nucleotide with moderate to deleterious *in silico* predictions (see **Table 1**). The variant is ultra-rare in gnomAD v4.1.0 with no homozygotes or alternative missense substitutions for Pro39. The paternally inherited frameshift variant, p.(Leu14Ilefs*20), is predicted to introduce a premature termination codon at nucleotide positions 100-102 after the transcription start site. Once again, the variant is ultra-rare in gnomAD v4.1.0 with no reported homozygotes.

Family 2

F2:II-1 is the first child born to non-consanguineous parents of Swiss ancestry. There is a family history of cystic fibrosis in F2:II-1's younger sister. F2:II-1 was born at 42-weeks' gestation via Cesarean section with Apgar scores of 6, 7 and 9 at 1, 5 and 10 minutes respectively. After an initial period of normal development, he presented at 10 months with new-onset horizontal nystagmus, mild grasp asymmetry and instability whilst sitting. This initial presentation was attributed to parainfectious cerebellitis in the context of an intercurrent enterovirus infection. There was normal to mildly increased lactate (max. 3.6 mmol/L). Brain MRI (see **Figure 1C**) and MRS were performed one month later which demonstrated symmetric focal T2 signal alterations corresponding to diffusion restriction on DWI in the subthalamic region of the transition from the capsula interna to the pedunculus cerebri, as well as in the dorso-medial pons on both sides, together with increased patchy lactate peaks in the frontoparietal white matter, all findings suggestive of an underlying mitochondrial disorder. As such, respiratory chain enzyme studies were arranged on skeletal muscle biopsy and skin fibroblasts, which both showed an isolated Complex I deficiency (see **Table 2**). Reduced Complex I activity was more pronounced in skeletal muscle compared to F2:II-1 fibroblasts. In skeletal muscle the residual Complex I activity was 10.5% compared to controls (expressed as activity ratios CI/CS: F2:II-1 0.02 (range 0.014- 0.028) normal range 0.19 +/- 0.04). The residual Complex I activity in F2:II-1 fibroblasts was 34% (expressed as activity ratios CI/CS: F2:II-1 0.10 (range 0.19-0.46) normal range 0.29 +/- 0.06). These findings were supportive of a mitochondrial disorder with a profound Complex I deficiency, however genetic testing at this time did not identify a known underlying molecular cause. A mitochondrial cocktail of carnitine, riboflavin, tocopherol and coenzyme Q10 was instituted but with no notable effect, other than the correction of a low plasma coenzyme Q10 level.

By one year of age, F2:II-1 displayed generalized muscular hypotonia with mild psychomotor delay and progressive mild microcephaly. At 14 months, walking whilst holding onto walls was possible. At age 2 years he was speaking in 2-word phrases but was not ambulating due to ataxia, and whilst the nystagmus had ceased, there was now progressive bilateral external ophthalmoplegia noted, with ocular nystagmus in the context of bilateral optic neuropathy. F2:II-1 went on to experience intermittent periods of motor developmental regression in the context of viral-induced respiratory deterioration with abundant secretion production. He also developed recurrent supraventricular tachycardia in light of a pre-excitation syndrome, together with predominantly left-sided hypertrophic cardiomyopathy which remained stable on serial follow-up. Gastrotomy feeding was commenced at 2.5 years of age due to increased feeding difficulties and failure to thrive.

A ketogenic diet was introduced at 3.5 years of age and well tolerated, with perceived slowing of disease progression, however he then developed a severe sleeping disorder. Cognitive development was normal, if not above average, with non-verbal IQ assessed between 114-137. There was continued slow deterioration of motor function over the following years, with increasing myopathic stiffness to the point of a loss of the majority of his motor skills. F2:II-1 encountered severe anxiety in his later years, making the sleepless nights intolerable at which point a rocking bed was arranged at 11 years of age to simulate the movement of a night train. This intervention considerably improved sleep, motor and cognitive functions.[1] After use of the bed was withdrawn for several months, F2:II-1 experienced an acute deterioration and passed away shortly after his 13th birthday. The family was then included in a trio WGS project for unsolved cases, which identified compound heterozygous candidate variants in *NDUFA5*.

The maternally inherited missense variant, c.1A>C;p.0? affects a highly conserved nucleotide in the start codon and therefore predicts a start-loss variant. *In silico* prediction tools consider this variant as deleterious or of uncertain significance (see **Table 1**). The variant is ultra-rare in gnomAD v.4.1.0 with no reported homozygotes. The paternally inherited variant c.183G>A is synonymous p.(Ala61=) but concerns the last nucleotide of exon 3 and therefore the adjacent splice donor site. Despite this, *in-silico* prediction did not predict a high probability of a mis-splicing event (SpliceAI acceptor loss = 0.12, donor loss = 0.06) (see **Table 1**). The variant is ultra-rare in gnomAD v.4.1.0 with no reported homozygotes.

Family 3

F3:II-2 was born to second-cousin consanguineous parents of Turkish ancestry. The 17-year-old sister, both parents, and the 7-year-old brother are clinically unaffected. On the maternal side, there is a history of two miscarriages in the third-to-fifth months of pregnancy, as well as two neonatal deaths. The maternal grandfather has a history of migraine headaches.

Prenatal and perinatal development were unremarkable. At 10 months of age, F3:II-2 experienced a febrile seizure with a normal EEG recorded at this time. At 2.5 years, early neurological symptoms emerged including nystagmus, bilateral optic atrophy, and gait instability. A markedly elevated lactate level (6.1 mmol/l) raised suspicion for mitochondrial dysfunction. By the age of three years, a cranial MRI was performed that revealed two oval-shaped hyperintensities in the substantia nigra, along with a subtle band-like signal increase in the periaqueductal region (see **Figure 1D**). The differential diagnosis at this stage included Leigh syndrome. Biochemical examination of individual mitochondrial energy metabolism enzymes from muscle showed a reduction of Complex I activity

relative to the marker enzyme citrate synthase (see **Table 2**). Other enzyme complexes (Complex II–IV) were within normal limits. These findings confirmed a mitochondriopathy with Complex I deficiency.

Due to bilateral cavus foot deformity with hallux valgus, Achilles tendon lengthening was performed at the ages of 10, 12, and 14 years. Initial singleton exome sequencing at age 16 years was inconclusive. F3:II-2 showed continued neurological decline. By age 18 years, she was no longer able to walk independently and required both a wheelchair and a rollator. Bilateral optic atrophy and gaze-evoked nystagmus persisted, while pupillary responses and fundoscopy remained normal. Ophthalmological examination revealed significantly reduced visual acuity and atrophy of the ganglion cell layer on optical coherence tomography. Visual acuity was stable when compared with previous examinations, and the ganglion cell layer atrophy had also been documented during previous pediatric inpatient evaluation. Cognitive function and mood were preserved. Notably, no neutropenia or cardiac phenotype were observed for F3:II-2.

Reanalysis of the original singleton exome sequencing data ultimately identified a homozygous synonymous variant in the *NDUFA5* gene. This is the same c.183G>A variant identified in F2:II-1 which impacts the last nucleotide of exon 3 and the splice donor site. Following the diagnosis of Leigh syndrome, based on clinical phenotype, MRI findings, muscle biopsy, and genetic testing, a therapeutic trial with 900 mg/day idebenone was initiated at 19 years of age. During regular follow-up visits every three months, visual acuity and possible side effects were monitored. The therapy was well tolerated but so far has not led to measurable clinical improvement in visual acuity.

Supplemental Materials and Methods

All *NDUFA5* variants were identified via genomic sequencing. Phenotype data were collected via direct assessment by study co-authors and medical record review. All procedures were followed in accordance with the ethical standards of the responsible committee on human experimentation. Informed consent was obtained for each study participant.

DNA Sequencing

For Family 1 (F1), whole genome sequencing (WGS) was performed using massively parallel sequencing (Nextera^{TM} DNA Flex Library Prep kit, Illumina) with a mean target coverage of 30x and a minimum of 90% of bases sequenced to at least 10x for nDNA and a minimum of 800x mean coverage for mtDNA. Data were processed, including read alignment to the reference genome (GRCh38) and the revised Cambridge Reference Sequence (rCRS) mitochondrial genome

(NC_012920.1). Variant calling for nDNA was performed using Cpipe,[2] or the functionally equivalent analysis with the Illumina Dragen System. For mtDNA, variants were called using Mutect2. Automated sex determination, relatedness and contamination checks were performed. For nDNA, variant analysis and interpretation within the selected target region (RefSeq genes +/- 1kb) was performed using Agilent Alissa Interpret. Variants were annotated against all RefSeq gene transcripts and reported in accordance with HGVS nomenclature. Genomic coordinates were generated by Cpipe and do not necessarily comply with HGVS guidelines. Copy number variants (CNVs) were screened for using an internal CNV detection tool, CXGo.[3] For mtDNA, a custom in-house analysis pipeline was used to detect large deletions and annotate the VCF file with variant information. Genes with proven disease association are considered during routine analysis. Curation of nDNA variants was phenotype-driven with pre-curated or custom gene lists used for variant prioritization. Since no likely cause of disease was identified in the Mendeliome, other candidate variants were considered.

For Family 2 (F2), DNA from venous peripheral blood was extracted using a Maxwell RSC 48 instrument (Promega). DNA concentration was measured using QuantiFluor Dyes on a Quantus Fluorometer (Promega) as described by the manufacturer. DNA was fragmented on a Covaris with targeted size of 350 bp. Subsequently, the TruSeq DNA PCR-Free Kit (Illumina) was used to prepare DNA libraries for whole genome sequencing according to the manufacturer's protocol with 1 μg DNA input. DNA libraries were subsequently sequenced on a NovaSeq 6000 (Illumina) as 150 bp paired ends with an average coverage of approximately 40x. Alignment of sequencing reads was to the human reference genome GRCh37/hg19. Variant calling and annotation were performed using VarSome Clinical (Saphetor SA, Lausanne, Switzerland). Copy number variants (CNVs) were analyzed using ExomeDepth integrated in the VarSome Clinical suite.

For Family 3 (F3), exome sequencing was performed as part of the GENOMIT project (https://www.genomit.eu), as previously described.[4] Briefly, genomic DNA was extracted from whole blood and fragmented following standard protocols. Target enrichment was performed using the Twist Exome 2.0 kit and sequencing was conducted on an Illumina NovaSeq 6000 platform in 100 bp paired-end mode, achieving an average coverage greater than 75× with 98.1% of the target region covered >20×. Sequence reads were aligned to the human reference genome GRCh37/hg19 using the Burrows-Wheeler Aligner (BWA). Variant calling for single nucleotide variants (SNVs) was performed with the Genome Analysis Toolkit (GATK v4.2.3.0), while insertions and deletions (indels) up to 20 bp were identified using Pindel. Copy number variants (CNVs) and structural variants (SVs) were detected through an integrative approach combining ExomeDepth, Manta, LUMPY, BreakDancer, and WHAMG. Data analysis and interpretation were conducted using the

open-source next-generation sequencing (NGS) analysis and clinical decision-support platform EVAdb (LIMS Production version 2022.05; https://github.com/mri-ihg/EVAdb). Variants in *NDUFA5* were confirmed by RNA sequencing (RNAseq) data.

RNA studies

RNA sequencing data for F1:II-1 and controls were mapped to the hg38 reference genome with the gap aware aligner program, STAR V2.7.3a. The 2-pass method within STAR[5] was utilized, with gencode.v41 gene annotation to enhancer mapping and the detection of unique splicing events. Quality of data was assessed with FastQC. Specific splicing pattern of the *NDUFA5* gene was observed using IGV.[6]

For F2:II-1, total RNA from control and F2:II-1 fibroblasts was isolated using the QIAGEN RNeasy Kit according to the manufacturer's instructions. Random oligohexamer primed RNA was reverse transcribed using the SuperScript II First-Strand cDNA Synthesis System (Invitrogen) according to the manufacturer's recommendations. One-twenty-fifth of single stranded cDNA were used as a template to amplify *NDUFA5* cDNA using gene specific oligonucleotides located in the exon 1-2 junction (forward primer: NDUFA5_Ex1-2_F 5'-GGGTGTGCTGAAGAAGACCACTG-3' and in exon 4 (reverse primer NDUFA5_Ex_4_R 5'- TTCTAATTGACCGCCTTGAAG-3'), respectively. Products were resolved on 1.2% (w/v) Agarose gel electrophoresis. Additionally, all RT-PCR fragments were sequenced using BigDye chemistry on an ABI3730XL genetic analyzer.

Strand-specific RNAseq of F3:II-2 and control fibroblast samples was performed using the TruSeq Stranded mRNA Sample Prep LS protocol (Illumina). [7,8] Data processing and analysis for F3:II-2 were carried out using the DROP pipeline, as described by Yepez et al.[9] For outlier detection, the sample was analyzed alongside a cohort of similarly processed in-house samples (total n = 758), following the approach detailed by Kopajtich et al.[10] and Yepez et al.[11]

Proteomics

Quad analysis of proband F1:II-1, affected sibling F1:II-2 and parental (F1:I-1, F1:I-2) PBMCs was performed as previously described.[12] Briefly, PBMCs were isolated from approximately 3 mL EDTA blood from adults and six unrelated age matched adult controls and 1-2 mL from proband, F1:II-2 and five age matched controls using SepMate columns (StemCell Technologies) according to manufacturer's instructions. PBMC pellets were resuspended in a lysis buffer (5 % SDS [w/v], 50 mM triethylammonium bicarbonate) and protein concentration estimated using the Pierce BCA Protein Assay Kit (Thermo Scientific). A total of 20 µg of protein was digested (in triplicate for

proband, sibling and parents, and single replicate for controls) using S-Trap micro columns (ProtiFi) according to the manufacturer's instructions at a 1:20 trypsin to protein ratio as previously described. Approximately 300 ng of peptides were analyzed using an Orbitrap Astral Mass Spectrometer (MS; Thermo Scientific) equipped with a Vanquish Neo UHPLC (Thermo Scientific) using a heated trap and elute setup. The columns were Acclaim PepMap nano-trap column (Dionex C18, 100 Å, 75 µm× 2 cm) and 5.5 cm high throughput µPAC Neo analytical column (Thermo Scientific). The eluents were water with 0.1 % v/v formic acid (solvent A) and 80 % acetonitrile (ACN) with 0.1 % v/v formic acid (solvent B). The peptides were separated on the analytical column using the following gradient at a flow rate of 750 nl/min: (i) 0-0.3 min 3-6% B, ii) 0.3-23 min 6-23.5% B, (iii) 23-26.7 min 23.5-40% B, (iv) 26.7-28.7 min 40-50% B, (v) 28.7-28.8 min 50-99% B and (vi) 28.8-30 min 99 %. The column was then equilibrated for 7 min before the next sample injection. The instrument was configured for data independent acquisition (DIA) where full MS resolutions were set to 120,000 and scanning from 380-980 m/z in profile mode. Full MS automatic gain control (AGC) target was 500 % with a maximum IT of 5 ms. DIA was carried out in the Astral analyzer with an isolation window of 2 m/z, normalized HCD collision energy of 27, normalized AGC target of 500 % and maximum injection time of 3 ms. The cycle time was kept to 0.6 s. Raw data were searched with Spectronaut® software (v.19.0.240606.62635, Huggins, Biognosys) using default settings with the following modifications for increased stringency and peptide quantification: (1) identification setting: exclude single hit proteins was selected, (2) identification setting: protein q-value cut-off was set to 0.01 and precursor and protein posterior error probability (PEP) to 0.01, (3) and quantification setting: Major and Minor Top N groups were unselected to allow for all precursors and peptides that meet the cut-off settings to be identified and quantified.

For F3:II-2, fibroblasts were analyzed using tandem mass tag (TMT)-multiplexing (Thermo Scientific) with mass spectrometry performed at the BayBioMS core facility of the Technical University of Munich, as described by Kopajtich et al.[10] with two minor modifications. Peptide fractionation was conducted using high-pH reverse-phase chromatography (Xbridge BEH 130 C18 3.5µm, 2.1x150m) instead of trimodal mixed-mode chromatography, and TMT labelling was performed using TMT 11-plex reagents (Thermo Scientific) rather than the TMT 10-plex. Data normalization and analysis were carried out using PROTRIDER[13] across a dataset comprising 249 fibroblast samples.

Relative Complex Abundance (RCA) plots were generated using the RCA tool available on the http://www.rdms.app website.[12] Topographical heatmaps were generated using the log2 fold-change between affected individuals, carrier parents, and controls to define the color of the relevant chain in

the cryo electron-microscopy derived structure of Human Complex I[14] (PBD ID: 5LDW) as previously described.[15] Range plots were generated using an R script described in Hock *et al.*[12] to calculate the protein abundance of NDUFA5 in carrier parents (F1:I-1, F1:I-2) relative to the median control abundance alongside the standard deviation (SD).

Respiratory chain enzymology

F2:II-1 skeletal muscle tissue was obtained from the quadriceps by surgical biopsy, and a fibroblast culture was established from skin obtained at the muscle biopsy site. Measurements of skeletal muscle homogenates and fibroblasts were performed as described previously.[16] Individual respiratory chain complex activities and the mitochondrial matrix enzyme citrate synthase (CS) were measured spectrophotometrically in a UV-1601 (Shimadzu) in 1 mL sample cuvettes maintained at 30 °C.[17] Enzyme activities in skeletal muscle from individual F3:II-2 were performed as previously described.[18,19] All values are expressed relative to the mitochondrial marker enzyme CS (mU/mU CS).

Respiratory chain complex assembly

Analysis of mitochondrial proteins was performed on skeletal muscle homogenates and isolated mitochondria from F2:II-1 and control fibroblasts separated by 4.5-13% Blue Native (BN)-polyacrylamide gel electrophoresis (PAGE) as described.[16]. Solubilization of mitochondrial complexes was performed using 5 mg digitonin per mg protein. Subsequent immunoblotting analysis was performed with antibodies against Complex I (NDUFA9; Abcam ab14713), Complex II (SDHA; Abcam ab14715), Complex III (UQCRC2, Abcam ab14745), Complex IV (COX4; Abcam ab33985) and Complex V (ATP5A, Abcam ab14730). For 2D BN-PAGE analysis, the BN-PAGE gel strip was separated on 10 % Tricine/sodium dodecyl sulphate (SDS)-PAGE followed by Coomassie staining. SDS-PAGE was performed as previously described.[20] BN-PAGE analysis of mitochondrial proteins from lymphoblastoid cell lines (LCL) was performed as previously described.[12] Subsequent immunoblotting utilized antibodies against Complex I (NDUFA9, in-house;[21] NDUFB6, in-house[22]), MCIA complex (NDUFAF1, in-house[22]), Complex III (UQCRC1, ThermoFisher 16D10AD9AH5), Complex IV (COX4, ab110261) and the above mentioned Complex II antibody. Protein concentrations were determined by bicinchoninic acid (BCA) assay and equal amounts loaded.

Molecular modeling

To predict the structures of WT and Arg23_Ala61del NDUFA5 the relevant sequences (Uniprot Q16718-1) were uploaded to ColabFold[23] for AlphaFold2 modeling[24] with AMBER relaxation enabled (num_relax=1). All other settings were default. The top-ranked model (pLDDT confidence

score) for each construct was aligned to NDUFA5 extracted from the experimentally determined Cryo-EM structure of human Complex I (PDB:5XTD)[25] using the *super* algorithm within the PyMOL Molecular Graphics System, Version 3.1.6.1, Schrödinger, LLC. Modelling of the quaternary structure of WT and Arg23_Ala61del NDUFA5 in complex with NDUFS2 (Uniprot O75306-1 lacking residues 1-44 containing the mitochondrial targeting sequence [MTS] and region interacting with the membrane arm of Complex I), NDUFS3 (Uniprot O75489-1 lacking MTS residues 1-36 and all residues C-terminal of Glu204 that interact with subunits in the N-module) and NDUFA7 was performed using Alphafold-multimer[26] via Colabfold. To align the modelled quaternary structures with the experimentally determined structure the core complex of NDUFS2, NDUFS3 and NDUFA7 were temporarily merged into a single object in each structure and the *align* algorithm used to determined pairwise RMSDs for each model against the experimental structure.

Zebrafish modeling

Adult zebrafish were housed in a recirculating water system at 28 °C under a 14 h light / 10 h dark photoperiod and fed twice daily. Embryos were obtained via standard breeding protocols.[27] Larvae were raised in embryo medium consisting of 0.03 % Instant Ocean and 0.0002 % methylene blue in reverse osmosis-distilled water. All experimental procedures complied with the Guide for the Care and Use of Animals[28] and adhered to the guidelines of Cipher Gene, Ltd, under the institutional laboratory animal use license (SYXK (Zhe) 2024-0021) granted by Department of Science and Technology of Zhejiang Province, China.

The zebrafish genome contains an ortholog of the human *NDUFA5* gene, designated *ndufa5* (ENSDARG00000039346). Orthology was assessed using the DIOPT Ortholog Finder,[29] which indicated 60 % protein sequence identity between human *NDUFA5* and zebrafish *ndufa5*. Single guide RNA (sgRNA) target sites were predicted using the CHOPCHOP online tool[30] and synthesized by GenScript. Two sgRNAs were designed (PAM sequence in lowercase): GAATCCTCTGCAGACATGACagg, TTTCTGTAAGCGGCGTCCTGggg. For targeted mutagenesis, fertilized embryos at the 1-2 cell stage were injected with ~1 nL of CRISPR complexes containing two sgRNAs (~90 ng/µL each) and Cas9 protein (250 ng/µL). At 24 hours post injection, a subset of injected embryos was pooled for Sanger sequencing to verify the mutagenesis efficacy using the TIDE (Tracking of Indels by DEcomposition) online tool.[31] Post hoc genotyping was performed after phenotypic assessment. Individual larvae were collected for sequencing and TIDE analysis to confirm the mutagenesis, and those with a TIDE efficiency below 5% were excluded from phenotypic data analysis.

For morphological inspection, at 5 days post fertilization (dpf) zebrafish larvae were positioned individually, dorsal side up, in a custom mini-well plate for bright-field imaging. Images were captured using a Touptek CCD camera mounted on a Nikon SMZ800N stereomicroscope at 2× magnification. Morphometric parameters, including eye distance and body length, were quantified using Fiji (ImageJ). Locomotion was studied at 5 dpf. Individual zebrafish larvae were transferred into separate wells of a flat-bottom 96-well microplate containing 200 μL of embryo medium. Locomotor activity was recorded using the DanioVision system with EthoVision XT software (Noldus). Prior to recording, larvae were acclimated for 30 min inside the device at 28 °C under dark conditions. Spontaneous swimming activity was measured for 15 min, followed by alternating dark/light periods (5 min each) to assess stimulus-evoked responses. For each larva, total distance moved and maximum velocity were quantified. As the response had not fully stabilized during the initial cycle, data from the first dark/light cycle were excluded from the analysis. To assess survival, zebrafish larvae were maintained in 100 mm Petri dishes containing 50 mL of embryo medium, with no more than 80 larvae per dish. The dishes were kept in a 28 °C incubator, and 50 % of the medium was replaced daily with fresh medium. Larvae were fed with paramecia every day and monitored twice daily, and dead individuals were removed at each inspection. Mortalities occurring at 0–1 dpf were excluded from the analysis to minimize the influence of injection-related damage.

For electrophysiology studies zebrafish larvae were paralyzed in 100 μM pancuronium (Sigma) and immobilized in 2 % low-melting point agarose within a recording chamber. The chamber was filled with embryo medium and positioned on the electrophysiology platform. Local field potential (LFP) recordings were obtained from optic tectum using a glass microelectrode filled with 2 M NaCl. Signals were amplified using an extracellular amplifier with high impedance head stage (1700, A-M Systems), low-pass filtered at 5 kHz, high-pass filtered at 1 Hz, and digitized at 10 kHz via a digital acquisition board (Measurement Computing). Data acquisition and analysis were performed using the open-source software DClamp. Ictal-like events were defined, as previously described,[32] as multi-spike discharges exceeding 5x baseline noise and lasting more than 500 ms. Interictal-like events were defined as smaller discharges with amplitudes exceeding 3x baseline noise and durations greater than 100 ms. The occurrence of electrographic epileptiform activity, including both ictal- and interictal-like events, was quantified for analysis.

Statistical analyses were performed using Prism 8 (GraphPad Software). Unpaired t tests were used for comparisons between two groups, and log-rank tests were applied for survival analysis. Statistical significance was defined as $^{*}p < 0.05$; $^{**}p < 0.01$; $^{***}p < 0.001$.

Supplemental References

1. Breuss, A., Strasser, M., Nuoffer, J.M., Klein, A., Perret-Hoigne, E., Felder, C., Stauffer, R., Wolf, P., Riener, R., and Gautschi, M. (2024). Nocturnal vestibular stimulation using a rocking bed improves a severe sleep disorder in a patient with mitochondrial disease. J Sleep Res *33*, e14153. 10.1111/jsr.14153.
2. Sadedin, S.P., Dashnow, H., James, P.A., Bahlo, M., Bauer, D.C., Lonie, A., Lunke, S., Macciocca, I., Ross, J.P., Siemering, K.R., et al. (2015). Cpipe: a shared variant detection pipeline designed for diagnostic settings. Genome Med *7*, 68. 10.1186/s13073-015-0191-x.
3. Sadedin, S.P., Ellis, J.A., Masters, S.L., and Oshlack, A. (2018). Ximmer: a system for improving accuracy and consistency of CNV calling from exome data. Gigascience *7*. 10.1093/gigascience/giy112.
4. Zech, M., Kopajtich, R., Steinbrucker, K., Bris, C., Gueguen, N., Feichtinger, R.G., Achleitner, M.T., Duzkale, N., Perivier, M., Koch, J., et al. (2022). Variants in Mitochondrial ATP Synthase Cause Variable Neurologic Phenotypes. Ann Neurol *91*, 225–237. 10.1002/ana.26293.
5. Dobin, A., Davis, C.A., Schlesinger, F., Drenkow, J., Zaleski, C., Jha, S., Batut, P., Chaisson, M., and Gingeras, T.R. (2013). STAR: ultrafast universal RNA-seq aligner. Bioinformatics *29*, 15–21. 10.1093/bioinformatics/bts635.
6. Robinson, J.T., Thorvaldsdottir, H., Winckler, W., Guttman, M., Lander, E.S., Getz, G., and Mesirov, J.P. (2011). Integrative genomics viewer. Nat Biotechnol *29*, 24–26. 10.1038/nbt.1754.
7. Brechtmann, F., Mertes, C., Matuseviciute, A., Yepez, V.A., Avsec, Z., Herzog, M., Bader, D.M., Prokisch, H., and Gagneur, J. (2018). OUTRIDER: A Statistical Method for Detecting Aberrantly Expressed Genes in RNA Sequencing Data. Am J Hum Genet *103*, 907–917. 10.1016/j.ajhg.2018.10.025.
8. Mertes, C., Scheller, I.F., Yepez, V.A., Celik, M.H., Liang, Y., Kremer, L.S., Gusic, M., Prokisch, H., and Gagneur, J. (2021). Detection of aberrant splicing events in RNA-seq data using FRASER. Nat Commun *12*, 529. 10.1038/s41467-020-20573-7.
9. Yepez, V.A., Mertes, C., Muller, M.F., Klaproth-Andrade, D., Wachutka, L., Fresard, L., Gusic, M., Scheller, I.F., Goldberg, P.F., Prokisch, H., and Gagneur, J. (2021). Detection of aberrant gene expression events in RNA sequencing data. Nat Protoc *16*, 1276–1296. 10.1038/s41596-020-00462-5.
10. Kopajtich, R., Smirnov, D., Stenton, S., Loipfinger, S., Meng, C., Scheller, I., Freisinger, P., Baski, R., Berutti, R., Behr, J., et al. Integration of proteomics with genomics and transcriptomics increases the diagnostic rate of Mendelian disorders.

11. Yepez, V.A., Gusic, M., Kopajtich, R., Mertes, C., Smith, N.H., Alston, C.L., Ban, R., Beblo, S., Berutti, R., Blessing, H., et al. (2022). Clinical implementation of RNA sequencing for Mendelian disease diagnostics. Genome Med *14*, 38. 10.1186/s13073-022-01019-9.
12. Hock, D.H., Caruana, N.J., Semcesen, L.N., Lake, N.J., Formosa, L.E., Amarasekera, S.S.C., Stait, T., Tregoning, S., Frajman, L.E., Bournazos, A.M., et al. (2025). Untargeted proteomics enables ultra-rapid variant prioritisation in mitochondrial and other rare diseases. Genome Med *17*, 58. 10.1186/s13073-025-01467-z.
13. Klaproth-Andrade, D., Scheller, I., Tsitsiridis, G., Loipfinger, S., Mertes, C., Smirnov, D., Prokisch, H., Yépez, V., and Gagneur, J. PROTRIDER: Protein abundance outlier detection from mass spectrometry-based proteomics data with a conditional autoencoder.
14. Zhu, J., Vinothkumar, K.R., and Hirst, J. (2016). Structure of mammalian respiratory complex I. Nature *536*, 354–358. 10.1038/nature19095.
15. Stroud, D.A., Surgenor, E.E., Formosa, L.E., Reljic, B., Frazier, A.E., Dibley, M.G., Osellame, L.D., Stait, T., Beilharz, T.H., Thorburn, D.R., et al. (2016). Accessory subunits are integral for assembly and function of human mitochondrial complex I. Nature *538*, 123–126. 10.1038/nature19754.
16. Jackson, C.B., Nuoffer, J.M., Hahn, D., Prokisch, H., Haberberger, B., Gautschi, M., Haberli, A., Gallati, S., and Schaller, A. (2014). Mutations in SDHD lead to autosomal recessive encephalomyopathy and isolated mitochondrial complex II deficiency. J Med Genet *51*, 170–175. 10.1136/jmedgenet-2013-101932.
17. Shepherd, D., and Garland, P.B. (1969). The kinetic properties of citrate synthase from rat liver mitochondria. Biochem J *114*, 597–610. 10.1042/bj1140597.
18. Feichtinger, R.G., Weis, S., Mayr, J.A., Zimmermann, F., Geilberger, R., Sperl, W., and Kofler, B. (2014). Alterations of oxidative phosphorylation complexes in astrocytomas. Glia *62*, 514–525. 10.1002/glia.22621.
19. Feichtinger, R.G., Zimmermann, F., Mayr, J.A., Neureiter, D., Hauser-Kronberger, C., Schilling, F.H., Jones, N., Sperl, W., and Kofler, B. (2010). Low aerobic mitochondrial energy metabolism in poorly- or undifferentiated neuroblastoma. BMC Cancer *10*, 149. 10.1186/1471-2407-10-149.
20. Laemmli, U.K. (1970). Cleavage of structural proteins during the assembly of the head of bacteriophage T4. Nature *227*, 680–685. 10.1038/227680a0.
21. Lazarou, M., McKenzie, M., Ohtake, A., Thorburn, D.R., and Ryan, M.T. (2007). Analysis of the assembly profiles for mitochondrial- and nuclear-DNA-encoded subunits into complex I. Mol Cell Biol *27*, 4228–4237. 10.1128/MCB.00074-07.

22. Dunning, C.J., McKenzie, M., Sugiana, C., Lazarou, M., Silke, J., Connelly, A., Fletcher, J.M., Kirby, D.M., Thorburn, D.R., and Ryan, M.T. (2007). Human CIA30 is involved in the early assembly of mitochondrial complex I and mutations in its gene cause disease. EMBO J *26*, 3227–3237. 10.1038/sj.emboj.7601748.
23. Mirdita, M., Schutze, K., Moriwaki, Y., Heo, L., Ovchinnikov, S., and Steinegger, M. (2022). ColabFold: making protein folding accessible to all. Nat Methods *19*, 679–682. 10.1038/s41592-022-01488-1.
24. Jumper, J., Evans, R., Pritzel, A., Green, T., Figurnov, M., Ronneberger, O., Tunyasuvunakool, K., Bates, R., Zidek, A., Potapenko, A., et al. (2021). Highly accurate protein structure prediction with AlphaFold. Nature *596*, 583–589. 10.1038/s41586-021-03819-2.
25. Guo, R., Zong, S., Wu, M., Gu, J., and Yang, M. (2017). Architecture of Human Mitochondrial Respiratory Megacomplex I(2)III(2)IV(2). Cell *170*, 1247–1257 e1212. 10.1016/j.cell.2017.07.050.
26. Evans, R., O'Neill, M., Pritzel, A., Antropova, N., Senior, A., Green, T., Žídek, A., Bates, R., Blackwell, S., Yim, J., et al. (2021). Protein complex prediction with AlphaFold-Multimer. bioRxiv. 10.1101/2021.10.04.463034.
27. Westerfield, M. (2000). The zebrafish book. A guide for the laboratory use of zebrafish (Danio rerio). 4th ed. (Univ. of Oregon Press, Eugene).
28. Animals, N.R.C.U.C.f.t.U.o.t.G.f.t.C.a.U.o.L. (2011). Guide for the Care and Use of Laboratory Animals, 8th edition (National Academy of Sciences.). 10.17226/12910.
29. Hu, Y., Flockhart, I., Vinayagam, A., Bergwitz, C., Berger, B., Perrimon, N., and Mohr, S.E. (2011). An integrative approach to ortholog prediction for disease-focused and other functional studies. BMC Bioinformatics *12*, 357. 10.1186/1471-2105-12-357.
30. Labun, K., Montague, T.G., Krause, M., Torres Cleuren, Y.N., Tjeldnes, H., and Valen, E. (2019). CHOPCHOP v3: expanding the CRISPR web toolbox beyond genome editing. Nucleic Acids Res *47*, W171–W174. 10.1093/nar/gkz365.
31. Brinkman, E.K., Chen, T., Amendola, M., and van Steensel, B. (2014). Easy quantitative assessment of genome editing by sequence trace decomposition. Nucleic Acids Res *42*, e168. 10.1093/nar/gku936.
32. Griffin, A., Carpenter, C., Liu, J., Paterno, R., Grone, B., Hamling, K., Moog, M., Dinday, M.T., Figueroa, F., Anvar, M., et al. (2021). Phenotypic analysis of catastrophic childhood epilepsy genes. Commun Biol *4*, 680. 10.1038/s42003-021-02221-y.
