# Supplementary material for: Bi-allelic variants in NDUFA5 cause a mitochondriopathy with complex I deficiency
Source: Am J Hum Genet. 2026 Mar 30;113(5):1108–21. doi: 10.1016/j.ajhg.2026.03.003 (PMC13277682; doi:10.1016/j.ajhg.2026.03.003)
Supplement: Document S2. Article plus supplemental information [file mmc2.pdf]

Bi-allelic variants in *NDUFA5* cause a mitochondriopathy with complex I deficiency

Authors

Natalie B. Tan, Matthias Gautschi, Michael Raum, ..., Jing Liu, David A. Stroud, Holger Prokisch

Correspondence

david.stroud@unimelb.edu.au (D.A.S.),
holger.prokisch@helmholtz-munich.de (H.P.)

We identify bi-allelic *NDUFA5* variants in four individuals from three families with mitochondrial complex I deficiency. Genomic, transcriptomic, proteomic, and biochemical studies across patient tissues, complemented by a zebrafish model, characterize a variable multisystem disease with cardiac, hematological, and Leigh syndrome-like neurological features, expanding the spectrum of complex I mitochondriopathies.

Tan et al., 2026, The American Journal of Human Genetics *113*, 1108–1121
May 7, 2026 © 2026 The Author(s). Published by Elsevier Inc. on behalf of American Society of Human Genetics.
https://doi.org/10.1016/j.ajhg.2026.03.003

REPORT

Bi-allelic variants in *NDUFA5* cause a mitochondriopathy with complex I deficiency

Natalie B. Tan,[1,2,3,16] Matthias Gautschi,[4,16] Michael Raum,[5,16] Daniella H. Hock,[1,2,6] Robert Kopajtich,[5,7] Jia Wang,[8] Xiao Qian,[8] Tanavi Sharma,[6] Timothy E. Green,[1] Jean-Marc Nuoffer,[4] Katrina M. Bell,[1,2] Katarzyna Pospieszny,[9] Tegan Stait,[1,2] Chloe Pike,[2] Michelle Cao,[2] Susan M. White,[1,2,3] David R. Thorburn,[1,2,3] Theresa Brunet,[5] Matias Wagner,[5,7,10] Wolfgang Müller-Felber,[10] Leopold Zeng,[11] Thomas Klopstock,[11,12,13] André Schaller,[14,17] Jing Liu,[8,17] David A. Stroud,[1,2,6,17,*] and Holger Prokisch[5,7,15,17,*]

Summary

NDUFA5 encodes a structural subunit of mitochondrial complex I (NADH:ubiquinone oxidoreductase) located in the peripheral arm of the enzyme complex. Complex I is the largest enzyme of the mitochondrial respiratory chain and is essential for oxidative phosphorylation. There are many well-characterized conditions associated with nuclear-encoded mitochondrial complex I dysfunction, including Leigh syndrome, leukoencephalopathy, lethal infantile mitochondrial disease, hypertrophic cardiomyopathy, and exercise intolerance. The vast majority of these nuclear-encoded mitochondrial complex I deficiencies are autosomal-recessive conditions. To date, variants in *NDUFA5* have not been associated with mitochondriopathy in humans. We identified a cohort of four individuals from three unrelated families with bi-allelic variants in *NDUFA5*. All individuals present with variable multisystem disease in the setting of a mitochondrial complex I deficiency, biochemically proven via an array of respiratory chain enzymology, blue native PAGE, and mass-spectrometry-based proteomics in peripheral blood mononuclear cells, lymphoblastoid cell lines, fibroblasts, and skeletal muscle. Transcriptomics and RT-PCR demonstrated aberrant mRNA expression in all affected individuals. Finally, we generated zebrafish *ndufa5* F0 mutants that exhibited defects of morphological development, locomotor deficits, and abnormal brain activity. Our data demonstrate that bi-allelic variants in *NDUFA5* cause a mitochondrial complex I deficiency, characterized by a variable multisystem phenotype that encompasses severe congenital heart defects, hematological abnormalities, and neurological involvement consistent with Leigh syndrome.

The nuclear gene *NDUFA5* (MIM: 601677) encodes a structural subunit of mitochondrial complex I (NADH:ubiquinone oxidoreductase), specifically located in the peripheral arm (Q module) of the enzyme complex. Complex I is the largest enzyme of the mitochondrial respiratory chain and is essential for oxidative phosphorylation. Experimental ablation of *Ndufa5* in mice demonstrates that a requirement for embryonic survival and conditional knockout in the central nervous system leads to partial complex I deficiency, resulting in mild chronic encephalopathy and late-onset motor deficits but notably without increased oxidative damage or neuronal loss.[1] Common single-nucleotide polymorphisms in *NDUFA5* have been associated with neurodevelopmental disorders such as autism in case-control and family-based studies.[2] This suggests a potential role for *NDUFA5* in neurodevelopmental processes, likely through its impact on mitochondrial function.

Complex I is an L-shaped assembly composed of 45 subunits encoded by 44 genes in mammals, including 14 highly conserved core subunits responsible for catalysis, including seven encoded by mitochondrial DNA (mtDNA), and a set of supernumerary or accessory subunits that contribute to stability, regulation, and assembly.[3] The enzyme contains a hydrophilic arm, which houses the NADH dehydrogenase active site and redox centers (flavin mononucleotide and multiple iron-sulfur clusters), and a membrane arm, which mediates proton translocation.[4–6] The majority of complex I in humans is stably associated with complex III (coenzyme Q-cytochrome *c* reductase)

[1]Murdoch Children's Research Institute, Parkville, VIC, Australia; [2]Victorian Clinical Genetics Services, Parkville, VIC, Australia; [3]Department of Paediatrics, The University of Melbourne, Parkville, VIC, Australia; [4]Department of Paediatrics, Division of Endocrinology, Diabetology and Metabolism, and Institute of Clinical Chemistry, Inselspital - University Hospital of Bern, University of Bern, Bern, Switzerland; [5]Institute of Human Genetics, School of Medicine and Health, Technical University of Munich, Munich, Germany; [6]Department of Biochemistry and Pharmacology, Bio21 Molecular Science and Biotechnology Institute, The University of Melbourne, Melbourne, VIC, Australia; [7]Institute of Neurogenomics, Helmholtz Zentrum München, Munich, Germany; [8]Cipher Gene Ltd, Chengdu, China; [9]University Institute of Diagnostic and Interventional Neuroradiology, Inselspital, University Hospital of Bern, University of Bern, Bern, Switzerland; [10]Division of Pediatric Neurology and Developmental Medicine and LMU Center for Children with Medical Complexity, Dr. von Hauner Children's Hospital, LMU Hospital, Ludwig-Maximilians-Universität, Munich, Germany; [11]Friedrich Baur Institute at the Department of Neurology, LMU University Hospital, LMU Munich, 80336 Munich, Germany; [12]German Center for Neurodegenerative Diseases (DZNE), 81377 Munich, Germany; [13]Munich Cluster for Systems Neurology (SyNergy), 81377 Munich, Germany; [14]Department of Human Genetics, Inselspital - University Hospital of Bern, University Bern, Bern, Switzerland; [15]German Center for Child and Adolescent Health (DZKJ), Partner Site Munich, Munich, Germany
[16]These authors contributed equally
[17]These authors contributed equally
*Correspondence: david.stroud@unimelb.edu.au (D.A.S.), holger.prokisch@helmholtz-munich.de (H.P.)
https://doi.org/10.1016/j.ajhg.2026.03.003.
© 2026 The Author(s). Published by Elsevier Inc. on behalf of American Society of Human Genetics.
This is an open access article under the CC BY license (http://creativecommons.org/licenses/by/4.0/).

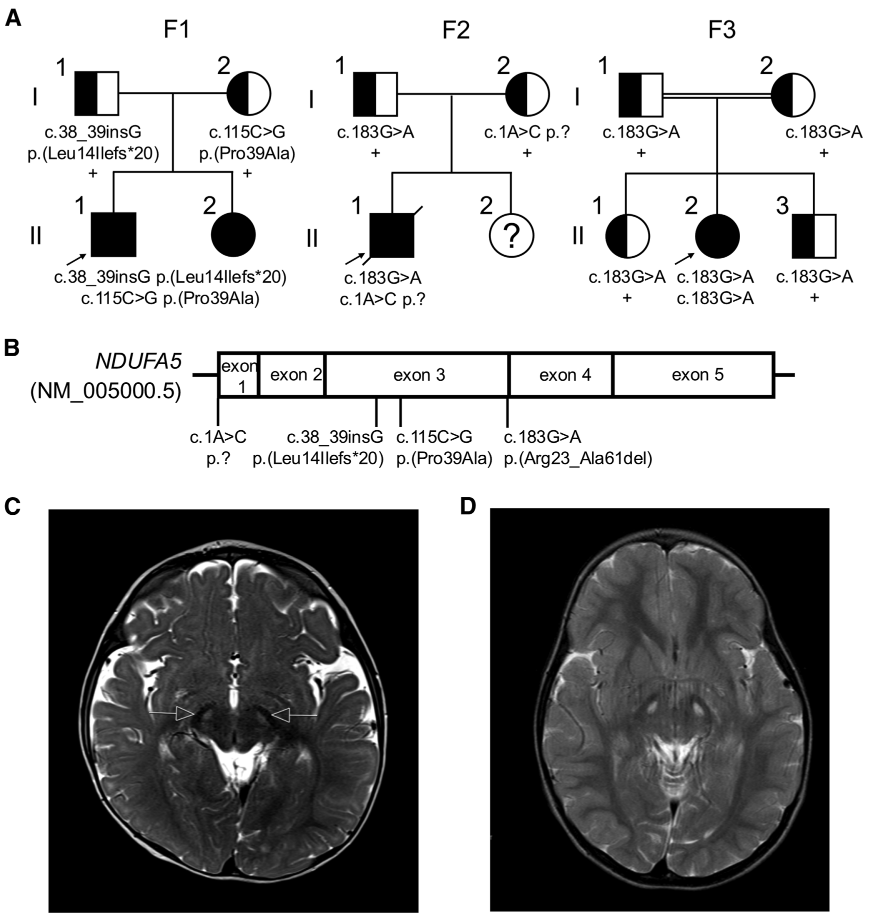

Figure 1. Bi-allelic variants in *NDUFA5* segregate with disease

(A) Pedigrees for the three families described in this study. Consanguineous F3:I-1 and F3:I-2 are second cousins.

(B) Schematic showing the *NDUFA5* gene and protein structure based on NCBI reference sequence GenBank: NM_005000.5. The location of variants described in this study are shown underneath the schematic.

(C) Brain MRI at 11 months of age for individual F2:II-1.

(D) Brain MRI at 3 years of age for individual F3:II-2.

and complex IV (cytochrome *c* oxidase) in respiratory chain supercomplexes (SCs), also known as the respirasome, of various stoichiometries.[7–9] Functionally, complex I oxidizes NADH (produced by the tricarboxylic acid cycle and β-oxidation), reduces ubiquinone to ubiquinol, and pumps four protons per NADH from the mitochondrial matrix to the intermembrane space, thereby contributing to the electrochemical gradient used for ATP production.[6] Dysfunction of complex I is implicated in a range of mitochondrial diseases, neurodegenerative disorders, and metabolic syndromes.[10,11]

Complex I deficiency is the most frequently encountered enzyme deficiency in mitochondrial disease, as it involves the largest respiratory chain complex and is composed of both nuclear DNA (nDNA) and mtDNA encoded subunits.[12,13] Nuclear-encoded complex I dysfunction typically manifests as Leigh syndrome (MIM: 256000 and MIM: 500017), leukoencephalopathy, lethal infantile mitochondrial disease, hypertrophic cardiomyopathy, and exercise intolerance[12]; however, it is well recognized that genotype-phenotype correlation for complex I disorders is poor. Traditional means for diagnosis have employed measurement of enzyme function, which is reliant on the sampling of affected tissue that necessitates invasive biopsy of, for example, skeletal muscle, or the even more difficult to access tissues of the liver and heart. Until the advent of sequencing technologies, enzymatic testing could identify a complex I deficiency but without specificity as to the underlying heritable cause. Here, we utilized a blend of traditional respiratory chain enzymology with modern molecular approaches to functional genomics—inclusive of genomic sequencing, RNA sequencing (RNA-seq), and mass spectrometry proteomics, from several tissues—to build a compelling evidence base for a recessive mitochondriopathy caused by pathogenic variants in *NDUFA5*.

In a collaborative effort facilitated via GeneMatcher,[14] we assembled a cohort of four individuals from three unrelated families with bi-allelic variants in *NDUFA5*. All procedures were followed in accordance with the ethical standards of the responsible committees on human experimentation at each center. Informed consent was obtained for each study participant. Detailed clinical descriptions and genetic histories are described in supplemental notes: case reports.

Family 1 comprises two affected siblings (F1:II-1 and F1:II-2) born to non-consanguineous parents of Korean descent (Figure 1A). Both siblings presented prenatally with intrauterine growth restriction and congenital heart disease, specifically coarctation of the aorta and biventricular hypertrophy confirmed postnatally, respectively. Notably, they exhibited a distinctive hematological phenotype at birth, with pancytopenia (anemia, thrombocytopenia, and leukopenia) and congenital red cell macrocytosis that required multiple red cell transfusions. Both siblings continue to experience a persistent but fluctuating neutropenia and lymphopenia (Table 1). While early developmental concerns were raised in the context of recovery from a complicated neonatal course, F1:II-1 has demonstrated gradual catch-up to largely age-appropriate milestones by age 2 years. Quad whole-genome sequencing (WGS) identified compound heterozygous variants in *NDUFA5* (Figure 1B): a maternally inherited

Table 1. Clinical phenotype information

	Family 1		Family 2		Family 3
**Individual**	**II-1**	**II-2**	**II-1**		**II-2**
Sex	M	F	M		F
Age	2.6 years (CGA 2.5 years)	6 months (CGA 5 months)	13 years (deceased)		19 years
Zygosity	compound heterozygous		compound heterozygous		homozygous
NDUFA5 variants (GenBank: NM_005000.5)	maternal c.115C>G (p.Pro39Ala)	paternal c.38_39insG (p.Leu14Ilefs*20)	maternal c.1A>C (p.0?)	paternal c.183G>A (p.Arg23_Ala61del)	biparental c.183G>A (p.Arg23_Ala61del)
Variant type	missense	frameshift	start-loss	synonymous/splice	synonymous/splice
gnomAD v.4.1.0 (AF; AC; hmz)	0.000001242; 2; 0	0.000003100; 5; 0	0.0002559; 413; 0	0.00001043; 16; 0	0.00001043; 16; 0
AlphaMissense	0.413	N/A	N/A	N/A	N/A
CADD	25.3	33	20.8	17.9	17.9
REVEL	0.617	N/A	0.452	N/A	N/A
PolyPhen2	0.778	N/A	0.979	N/A	N/A
SpliceAI Δ score[a]	0.01	0.04	0.01	0.12	0.12
Gestation	36 + 6 weeks	36 + 6 weeks	42 weeks		39 weeks
Prenatal	IUGR (<1st centile); fetal ECHO (21 + 2): moderate to severe hypoplastic aortic arch; left to right heart size discrepancy (LV smaller than RV)	IUGR (≪1st centile); fetal ECHO (35 + 5): distally tortuous aortic arch without clear obstruction, mildly small left-sided structures with an apex forming LV, mild biventricular hypertrophy with preserved systolic function	N/A		N/A
Growth	small stature, preserved head circumference (OFC 36%, wt 1%, lt 6%)	small stature, preserved head circumference (OFC 17%, wt 5%, lt <1%)	microcephaly (progressive)		normal
Development	mild developmental delay (predominantly language), age-appropriate catch-up by 2 years	normal development (at CGA 4 months)	normal cognition (non-verbal IQ 137 at 12 years), initially normal motor development, progressively delayed after 10 months		normal
Metabolic	neonatal hyperlactatemia (resolved by ∼6 months)	neonatal hyperlactatemia (resolved)	lactate levels normal to max. 3.6 mmol/L		elevated lactate levels normal to max. 6.1 mmol/L
Neurology	N/A	N/A	generalized hypotonia, ataxia (2 years), developmental motor regression triggered by intercurrent infection, progressive myopathy (stiffness), respiratory and gastrointestinal dysfunction (gastrostomy)		cerebellar ataxia; neuropathy; recurrent migraine headache
Ocular	N/A	N/A	infantile horizontal nystagmus (10 months); bilateral external ophthalmoplegia (2 years); bilateral optic neuropathy		nystagmus; bilateral optic atrophy

(*Continued on next page*)

Table 1. *Continued*

Individual	Family 1 II-1	Family 1 II-2	Family 2 II-1	Family 3 II-2
Hematology	pancytopenia (anemia, thrombocytopenia, leukopenia) on initial FBC at birth; congenital red cell macrocytosis (requiring multiple red cell transfusions, first on day 12); persistent but fluctuating cytopenias (neutropenia, lymphopenia)	congenital macrocytic anemia (requiring red blood cell transfusion at 1 month); lymphopenia (mild $CD4^+$ T cell and $CD19^+$ B cell); persistent neutropenia	normal blood counts, normal coagulation tests	normal blood counts, normal coagulation tests
Cardiology	congenital heart disease: coarctation of the aorta (repair 8 days of life, intraoperative thymectomy), ASD; persistent pulmonary arterial hypertension due to elevated biventricular end diastolic pressures; sinus node dysfunction ("chaotic atrial rhythm" in the postoperative setting, now resolved)	congenital heart disease: tortuous aortic arch, bicuspid aortic valve, small fenestrated ASD, mild LPA stenosis; mild biventricular hypertrophy (abnormal appearance of myocardium)	recurrent supraventricular tachycardia in light of a pre-excitation syndrome; hypertrophic cardiomyopathy (LV)	N/A
Other	congenital hirsutism (resolved); sacral dimple (spinal ultrasound NAD); left vocal cord palsy; hypospadias with bilateral hydroceles (awaiting surgery)	congenital hirsutism (resolved); sacral dimple, not midline (spinal ultrasound NAD); tongue tie release (1 month)	transitory increase of liver enzymes with intercurrent viral infection, consistently normal function and imaging, normal renal function	bilateral cavus foot deformity with hallux valgus
Investigations	brain MRI (3 weeks): mildly prominent ventricles; urine metabolic screen and organic acids: consistent with lactic acidosis; plasma amino acids: elevated alanine, otherwise non-diagnostic profile; normal chromosome breakage studies; normal free and total carnitine	normal hip ultrasound (breech at 31 + 6); normal screening cranial ultrasound; normal screening abdominal ultrasound	brain MRI/MRS (11 months): focal T2 signal alteration in the subthalamic region of the transition from the capsula interna to the pedunculus cerebri, as well as in the dorsomedial pons on both sides; increased patchy lactate peaks in the frontal to (markedly pronounced) parietal white matter; muscle biopsy: profound isolated complex I deficiency	brain MRI (3 years): two oval-shaped hyperintensities detected in the substantia nigra, along with a subtle band-like periaqueductal signal increase; muscle biopsy: profound isolated complex I deficiency

CGA, corrected gestational age; IUGR, intrauterine growth restriction; ECHO, echocardiogram; LV, left ventricle; RV, right ventricle; OFC, occipitofrontal circumference; wt, weight; lt, length; ASD, atrial septal defect; NAD, no abnormality detected; AF, allele frequency; AC, allele count; hmz, homozygotes; N/A, not applicable.

[a]SpliceAI Δ score is the delta score of a variant, defined as the maximum of (DS_AG, DS_AL, DS_DG, DS_DL). Generated with unmasked scores and maximum distance of 5,000 bp.

highly conserved missense variant c.115C>G (p.Pro39Ala) that is ultra-rare in gnomAD v.4.1.0 with no homozygotes or alternative missense substitutions for Pro39, and a paternally inherited frameshift variant c.38_39insG (p.Leu14Ilefs*20) that is also ultra-rare with no reported homozygotes (Table 1).

Family 2 is a male (F2:II-1) born to non-consanguineous Swiss parents (Figure 1A). He presented at 10 months of age with new-onset horizontal nystagmus, mild grasp asymmetry, and instability while sitting, initially attributed to an enterovirus infection but progressing to generalized muscular hypotonia, bilateral ophthalmoplegia, and hypertrophic cardiomyopathy (Table 1). There was normal to mildly increased lactate (maximum 3.6 mmol/L). Brain magnetic resonance imaging (MRI) (Figure 1C) and MR spectroscopy (MRS) were performed one month later, which demonstrated symmetric focal T2 signal alterations corresponding to diffusion restriction on diffusion-weighted imaging in the subthalamic region of the transition from the capsula interna to the pedunculus cerebri as well as in the dorsomedial pons on both sides, together with increased patchy lactate peaks in the frontoparietal white matter, all findings suggestive of an underlying mitochondrial disorder. Respiratory chain enzyme studies on skeletal muscle biopsy and skin fibroblasts both showed an isolated complex I deficiency (Table 2). Gastrotomy feeding was commenced at 2.5 years of age due to increased feeding difficulties and failure to thrive. A ketogenic diet was introduced at 3.5 years of age and well tolerated, with perceived slowing of disease progression. Cognitive development was normal; however, there was continued slow deterioration of motor function over the following years, with increasing myopathic stiffness to the point of a loss of the majority

Table 2. **Respiratory chain enzyme activities in isolated mitochondria from cultured skin fibroblasts and skeletal muscle homogenates of individuals F2:II-1 and F3:II-2**

	CS[a]	CI/CS	CII/CS	CIII/CS	CIV/CS	CV/CS
F2:II-1						
Fibroblasts						
F2:II-1	204 (113%)	**0.10 (34%)**	0.42 (127%)	0.71 (118%)	0.88 (117%)	0.13 (65%)
Range (controls)	134–228	0.19–0.46	0.17–0.52	0.35–0.87	0.42–1.11	0.12–0.38
Controls (*n* = 22)	181 ± 29	0.29 ± 0.06	0.33 ± 0.09	0.60 ± 0.15	0.75 ± 0.18	0.20 ± 0.08
Skeletal muscle						
F2:II-1	181 (172%)	**0.02 (10.5%)**	0.22 (105%)	0.61 (78%)	0.95 (82%)	0.50 (128%)
Range (controls)	70–173	0.14–0.28	0.14–0.36	0.50–1.11	0.57–1.76	0.17–0.66
Controls (*n* = 26)	105 ± 25	0.19 ± 0.04	0.21 ± 0.05	0.78 ± 0.15	1.16 ± 0.28	0.39 ± 0.13
F3:II-2						
Skeletal muscle						
F3:II-2	199 (133%)	**0.05 (36%)**	0.26 (144%)	2.54 (17%)	2.10 (231%)	0.50 (119%)
Range (controls)	150–338	0.14–0.35	0.18–0.41	1.45–3.76	0.91–2.24	0.42–1.26

Values in parentheses present the activities as a percentage of the lowest value of control range. OXPHOS complex measurements in complex I in F2:II-1- and F3:II-2-derived fibroblasts and skeletal muscle are consistent with an isolated complex I deficiency when indicated in bold.
[a]CS activity (mU/mg homogenate protein).

of his motor skills. He died at 13 years of age. Trio WGS identified compound heterozygous variants in *NDUFA5* (Figure 1B): a maternally inherited start-loss variant c.1A>C (p.0?) and a paternally inherited synonymous variant c.183G>A (p.Ala61=) located at the splice donor site of exon 3 (Table 1). Despite this, *in silico* tools did not predict a high probability of a mis-splicing event (SpliceAI acceptor loss = 0.12, donor loss = 0.06). Both variants are ultra-rare in gnomAD v.4.1.0, with no reported homozygotes.

Family 3 includes a female proband (F3:II-2) born to second-cousin consanguineous parents of Turkish ancestry (Figure 1A). Prenatal and perinatal development were unremarkable. At 10 months of age, she experienced a febrile seizure with a normal EEG recorded. She presented at 2.5 years with nystagmus, bilateral optic atrophy, and gait instability (Table 1). A markedly elevated lactate level (6.1 mmol/L) raised suspicion for mitochondrial dysfunction. By the age of 3 years, a brain MRI was performed that revealed two oval-shaped hyperintensities in the substantia nigra along with a subtle band-like signal increase in the periaqueductal region (Figure 1D). These results, together with respiratory chain enzymology on skeletal muscle biopsy that revealed an isolated complex I deficiency (Table 2), confirmed a mitochondriopathy. F3:II-2 showed continued neurological decline and by 18 years was no longer able to walk independently, requiring both a wheelchair and a rollator. Notably, no neutropenia or cardiac phenotypes were observed for F3:II-2. Singleton exome sequencing identified the same synonymous *NDUFA5* c.183G>A (p.Ala61=) variant found in F2:II-1 (Figure 1B and Table 1).

To investigate the functional consequence of these *NDUFA5* variants, we first analyzed their transcriptomic impact. The frameshift variant c.38_39insG (p.Leu14Ilefs*20) in family 1 siblings F1:II-1 and F1:II-2 occurs in exon 2 and is predicted to introduce a premature termination codon. RNA-seq of a lymphoblastoid cell line (LCL) derived from F1:II-1 suggests that the frameshift variant undergoes nonsense-mediated mRNA decay (NMD), with the frameshift variant detected in approximately 10% of reads indicating a loss of transcripts from the frameshift allele. However, this skewing is an under-representation, as the RNA-seq data also reveal the skipping of exon 2, within which the frameshift variant is located, occurring in approximately 17% of reads (Figure 2A). Exon 2 is a 15-amino-acid in-frame coding exon, the skipping of which would not be predicted to induce NMD. Of note, there is an annotated transcript of *NDUFA5* (GenBank: NM_001282419.3), which does not utilize exon 2, thus supporting the existence of natural isoforms that tolerate the exclusion of exon 2. While *in silico* splice predictions for this frameshift variant are not highly suggestive of a mis-splicing outcome (SpliceAI $\Delta = 0.04$ accepter loss, $\Delta = 0.02$ donor loss), the detection of exon 2 skipping in F1:II-1 may reflect enhancement of a non-canonical splicing phenomenon that is occurring at low frequency. SpliceAI confirms that this exon 2 skipping event occurs at low frequency in controls with a GTEx lymphocyte per-sample average read count of 3. Notably, the downstream missense variant in family 1 siblings, c.115C>G (p.Pro39Ala), shows allele balance bias with approximately 72% of transcripts retaining the missense variant (Figure 2B), which is also supportive of a loss of transcripts from the frameshift allele. Collectively, these data are concordant with two distinct consequences of the in *trans* frameshift allele: nonsense-mediated decay of transcripts

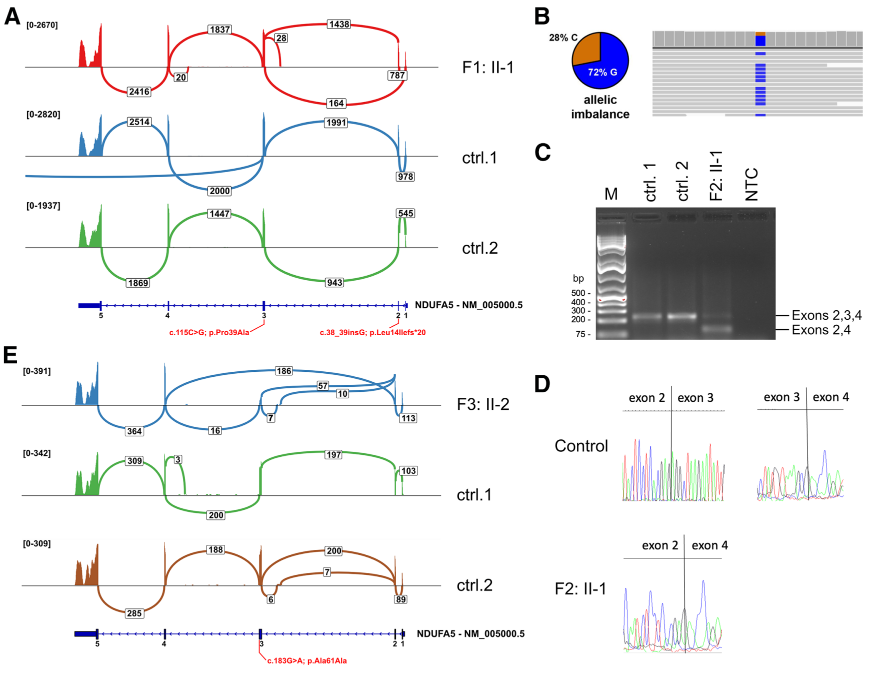

Figure 2. RNA studies

(A) Sashimi plot visualization of RNA-seq in F1:II-1 (top) and two control individuals (middle and bottom). The *NDUFA5* frameshift variant, c.38_39insG, results in two distinct outcomes for this allele: the introduction of a premature termination codon, which predisposes to nonsense-mediated mRNA decay for transcripts retaining exon 2, and the skipping of exon 2, which leads to an in-frame deletion of 15 amino acids.

(B) Allelic imbalance for F1:II-1, showing allele balance bias toward the missense variant c.115C>G (p.Pro39Ala) located in exon 3.

(C) Transcript cDNA analysis of *NDUFA5* in F2:II-1 and two controls. Individual F2:II-1 has an additional *NDUFA5* transcript species compared to the control samples. NTC, no template control.

(D) Sequence analysis of *NDUFA5* cDNA from F2:II-1 shows aberrant splicing due to the c.183G>A variant by skipping of exon 3 (shown are the exon-exon junctions).

(E) Sashimi plot visualization of RNA-seq in F3:II-2 (top) and two control individuals (middle and bottom). The *NDUFA5* splice variant, c.183G>A, leads to skipping of exon 3 in the majority of reads.

retaining exon 2 and increased exon 2 skipping leading to the in-frame deletion of 15 amino acids.

Family 2 proband, F2:II-1, was compound heterozygous for a start-loss c.1A>C (p.0?) variant and a synonymous c.183G>A variant that impacts upon the splice donor junction of exon 3. The same synonymous variant is homozygous in family 3 proband F3:II-2 (Figure 1A). While the *in silico* prediction tool SpliceAI does not predict a significant mis-splicing outcome (predicted splice donor loss score: 0.02), transcript analysis of cDNA from F3:II-2-derived fibroblasts revealed an additional smaller product in F2:II-1 (Figure 2C), which was confirmed by Sanger sequencing (Figure 2D) to lack exon 3. This splice defect was confirmed and quantified by RNA-seq on fibroblasts from F3:II-2. RNA-seq analysis of F3:II-2, who is homozygous for the c.183G>A variant, revealed exon 3 skipping in the majority of reads with only 10%–20% correctly spliced transcripts (Figure 2E), implicating the c.183G>A variant as causative. Importantly, skipping of exon 3 does not lead to a frameshift event but rather an in-frame deletion of 39 amino acids, which corresponds to approximately one-third of the 116-amino-acid NDUFA5 and therefore should be interpreted as p.Arg23_Ala61del.

To investigate the impact of the missense variant c.115C>G (p.Pro39Ala) variant identified in family 1, whole-cell proteomics was performed on quad peripheral blood mononuclear cells (PBMCs) from F1:II-1 and F1:II-2 carrying a maternally inherited p.Pro39Ala in *trans* with a paternally inherited c.38_39insG (p.Leu14Ilefs*20) and their unaffected carrier parents (Figure 1A). Proteomics-based relative complex abundance (RCA) analysis showed a significant and isolated reduction in the abundance of complex I to 23% and 19% in affected siblings F1:II-1 and F1:II-2, respectively, relative to pediatric controls (Figure 3A). NDUFA5 was not detected in PBMCs from the affected siblings but was well detected (3–5 peptides) in age-matched controls. As a consequence, both family 1 siblings meet the threshold for a major defect by RCA (≤65% relative to controls or ≤75% with absent detection of the protein of interest in the proband with >2 peptides detected in controls) defined by Hock et al.[15] Parental PBMC samples for family 1 showed a complex I RCA abundance of 84% in the father (F1:I-1) and 81% in the mother (F1:I-2) (Figure 3B), with NDUFA5 protein detected at 33% control median in the father (F1:I-1) and 31% in the mother (F1:I-2) (Figure 3C). Importantly, NDUFA5 levels were outside of the control range (83%–148% control median) in both parents, suggesting that the maternally inherited missense p.Pro39Ala variant has a similar impact on NDUFA5 abundance as the paternally inherited frameshift variant that undergoes NMD (Figure 2A). Topographical heatmap of the fold changes of complex I subunits in probands from family 1 also showed reduced abundance of all subunits across the complex (Figure 3D) except for NDUFAB1, which has a dual localization with members of the Leu-Tyr-Arg motif (LYRM) protein family in the mitochondrial matrix.[16]

Proteomics was also performed on fibroblasts from the proband of family 3 (F3:II-2) who is homozygous for the c.183G>A (p.Arg23_Ala61del) variant, which results in an in-frame deletion of 39 amino acids that escapes NMD. Complex I abundance in F3:II-2-derived fibroblasts was 84% control median (Figure 3A), impacting subunits from across the complex (Figure 3D). While this result

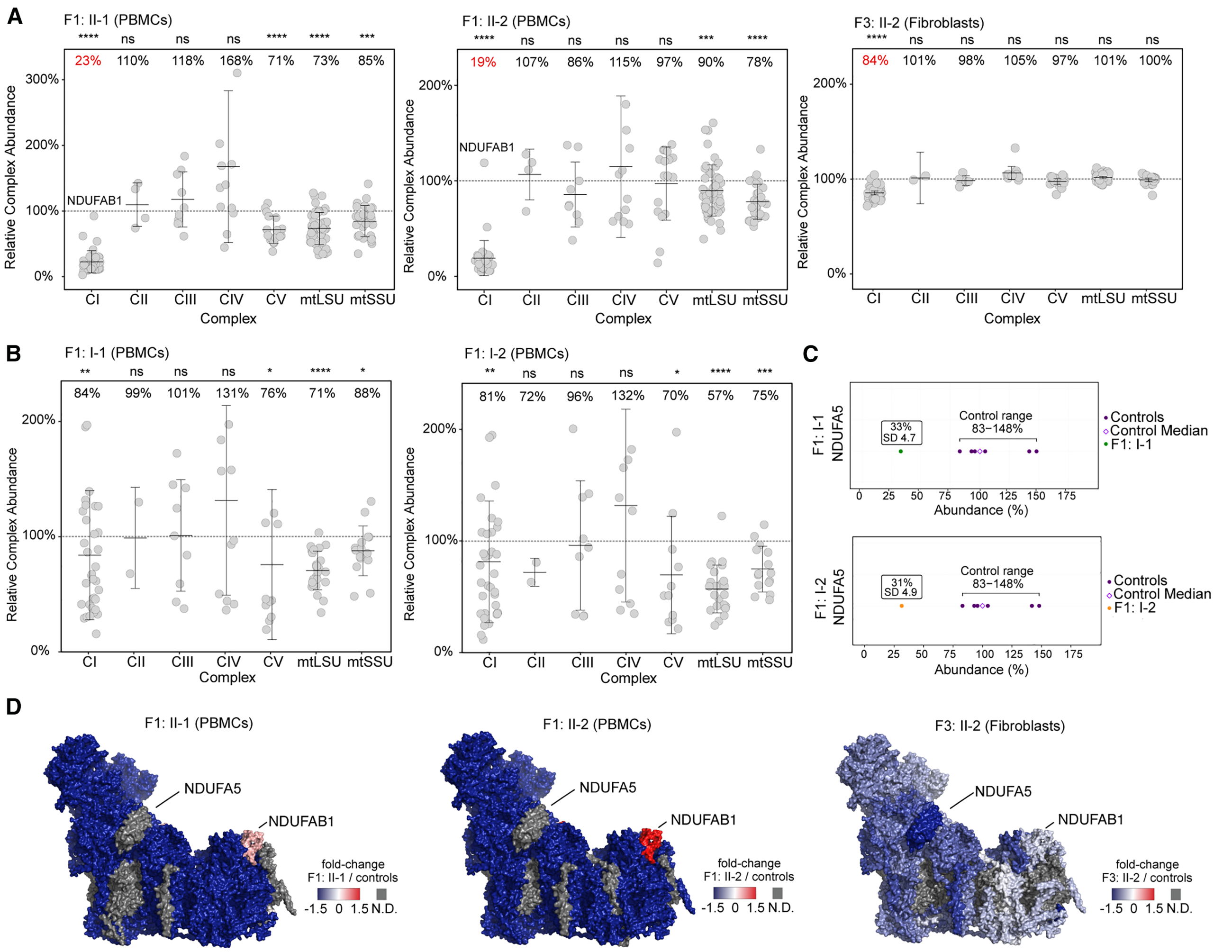

Figure 3. Proteomic studies

(A) Relative complex abundance (RCA) of oxidative phosphorylation (OXPHOS) complexes and mitoribosome subunits of peripheral blood mononuclear cells (PBMCs) from family 1 proband (F1:II-1) and affected sibling (F1:II-2) and fibroblasts from family 3 proband (F3:II-2). Middle bar represents mean complex abundance. Upper and lower bars represent 95% confidence interval. Significance was calculated from a two-sided t test between the individual protein means. ****$p < 0.0001$, ***$p < 0.001$, **$p < 0.01$, *$p < 0.05$; ns, not significant ($p > 0.05$). CI–CV, OXPHOS complexes I–V; mtLSU, mitoribosome large subunit; mtSSU, mitoribosome small subunit.

(B) RCA of PBMCs from family 1 carrier parents (F1:I-1 and F1:I-2). Middle bar represents mean complex abundance. Upper and lower bars represent 95% confidence interval. Significance was calculated from a two-sided t test between the individual protein means. ****$p < 0.0001$, ***$p < 0.001$, **$p < 0.01$, *$p < 0.05$; ns, not significant ($p > 0.05$). CI–CV, OXPHOS complexes I–V; mtLSU, mitoribosome large subunit; mtSSU, mitoribosome small subunit.

(C) Abundance range of NDUFA5 protein in PBMCs from F1:I-1 and F1:I-2 (carriers) relative to the median (purple diamond) of six individual adult controls ($n = 6$, purple dots). SD, standard deviation.

(D) Topographical heatmap of the fold-change values of complex I subunits in probands F1:II-1, F1:II-2, and F3:II-2 relative to controls. PDB: 5LDW. N.D., not detected (shown as gray in images).

does not meet the criteria for a major or minor RCA defect,[15] it is important to note that we analyzed an unaffected tissue, fibroblast cells, where canonical splicing was observed in ~25% of reads (Figure 2E). This is consistent with the complex I defect in individual F2:II-1 being more pronounced in muscle than in fibroblast cells (Table 2).

To investigate the impact on complex I and respiratory chain SC assembly, LCLs, fibroblasts, and skeletal muscle homogenates from the probands of family 1 (F1:II-1) and family 2 (F2:II-1) were analyzed by blue native (BN)-PAGE. As can be seen in Figure 4A, BN-PAGE and immunoblotting with antibodies against NDUFA9 (localized in the peripheral complex I Q module[17]) and NDUFB6 (complex I membrane arm P_D module, itself composed of P_D-a and P_D-b, also known as the ND4 and ND5 module, respectively[3]) identified two complex-I-containing SC assemblies that were less abundant in F1:II-1 LCLs compared to control. A similar reduction in SCs was seen in F2:II-1 fibroblasts compared to a complex II disease control (Figure 4B) and additionally resolved using 2D BN-PAGE (BN-PAGE followed by SDS-PAGE) (Figure 4C). Complex III (the free complex III dimer, $CIII_2$) was increased in abundance in both probands (Figures 4A and 4B), likely

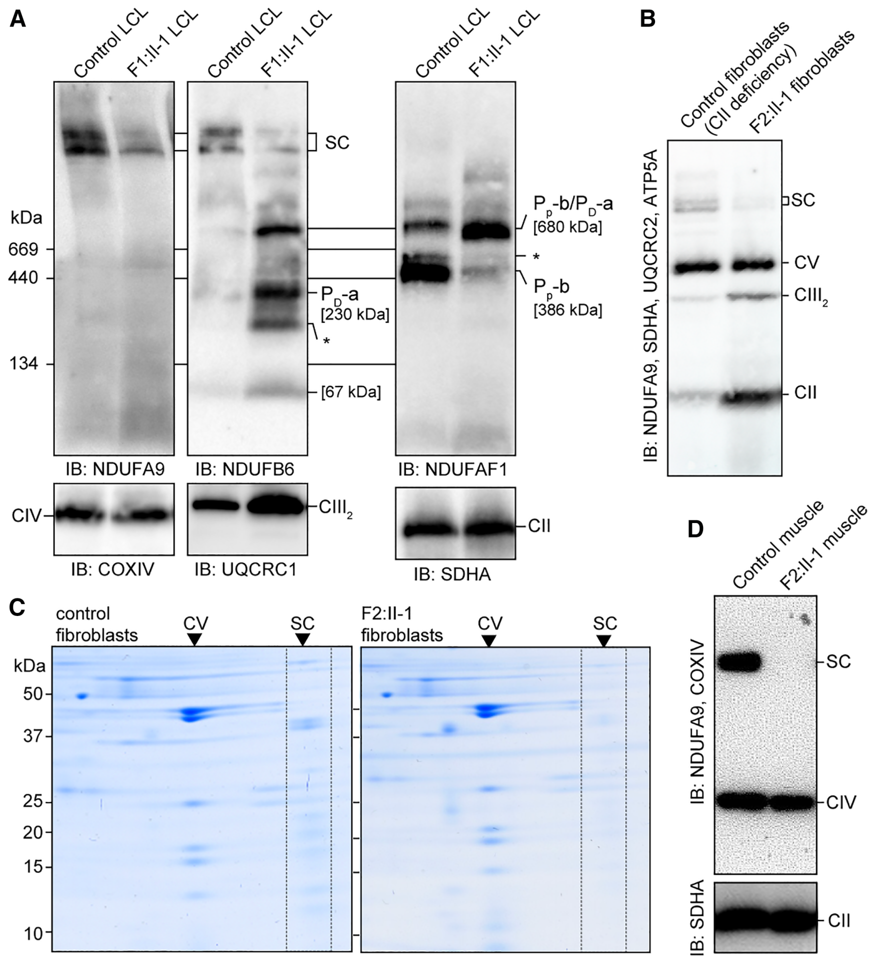

Figure 4. Complex I assembly studies

(A) BN-PAGE and immunoblotting (IB) on mitochondria from control and F1:II-1 lymphoblastoid cell lines (LCLs) using the indicated antibodies. Subcomplex assignment is based on the published nomenclature, which includes reference to molecular weights as first reported.[17] Asterisk (*) indicates non-specific or assigned band. CII, complex II; CIII, complex III; CIV, complex IV.

(B) As for (A) but utilizing a cocktail of antibodies for CI (NDUFA9), CII (SDHA), CIII (UQCRC2), and CV (ATP5A) on F2:II-1 and control (CII deficiency) fibroblast mitochondria.

(C) Comparison of the OXPHOS proteome via 2D BN-PAGE of F2:II-1 and control fibroblast mitochondria.

(D) As for (A) but utilizing the indicated antibodies to decorate CI containing SCs, CIV (COXIV), and CII.

due to the reduced amount of SC assembled complex I compared to controls. Interestingly, complex I and the SC was completely absent in F2:II-1 skeletal muscle homogenate, while free complex IV and complex V were unchanged (Figure 4D). These findings concord with the biochemical measurements of reduced complex I activity (Table 2) with a more pronounced effect seen in F2:II-1's skeletal muscle compared to fibroblasts. When considering that the start-loss variant in F2:II-1 is most likely to produce no protein, these findings likely represent the sole impact of the in-frame exon 3 skipping variant, c.183G>A, and are consistent with the broad reduction in complex I subunits seen in fibroblasts from F3:II-2 (Figure 3D) who is homozygous for the same c.183G>A variant. We next asked which step of complex I biogenesis is impacted in F1:II-1 mitochondria. Complex I assembles through a stepwise coalescence of mtDNA encoded subunits (ND1–7 and ND4L) with discrete modules of nuclear encoded core and accessory subunits.[3,17,18] NDUFA5 assembles with other Q-module subunits—NDUFS2, NDUFS3, NDUFS7, and NDUFS8—as a subcomplex, which joins the mtDNA encoded ND1 and several accessory subunits—NDUFA3, NDUFA8, and NDUFA13—to form the Q/P_P-a (Q/ND1[3]) intermediate.[17] In parallel, an intermediate containing ND2, ND3, ND4L, and ND6 and accessory subunits NDUFC1 and NDUFC2 is formed through the action of the mitochondrial complex I intermediate assembly (MCIA) assembly factors, resulting in a stable 386 kDa P_P-b (also known as the ND2 module when not associated with MCIA[3]) assembly that is readily resolved on BN-PAGE using antibodies against NDUFAF1 (Figure 4A, top right).[17,18] Formation of this intermediate is strongly inhibited in F1:II-1 LCLs compared to control. Moreover, the 230 kDa P_D-a intermediate—ND4, NDUFB1, NDUFB5, NDUFB6, NDUFB10, and NDUFB11, a 67 kDa pre-P_D-a assembly lacking ND4—and the 680 kDa P_P-b/P_D-a intermediate[17] strongly accumulate in F1:II-1 LCLs (Figure 4A). Taken together, these results suggest that, in the absence of NDUFA5, assembly of complex I is blocked at the penultimate step of biogenesis where Q/P_P-a and P_P-b/P_D-a join to form the Q/P intermediate (complex I lacking the N module).[17]

We also investigated the potential impact of each variant on NDUFA5 protein and its interactors. Figure 5A shows the location of NDUFA5 in the cryogenic electron microscopy (cryo-EM) structure of human complex I.[19] NDUFA5 makes protein-protein interactions with Q-module subunits NDUFS2 and NDUFS3 as well as accessory subunits NDUFA7 and NDUFA10.[4,19] These interactions occur through the N-terminal region of NDUFA5 with NDUFS2, α helix 1 with NDUFA10, α helices 2 and 4 with NDUFS3, and the C-terminal region with NDUFS2, NDUFS3, and NDUFA7. The nonsense variant p.Leu14Ilefs*20 in the family 1 siblings results in loss of α helices 2–4 and all surfaces that interact with NDUFS3, the C-terminal region interacting with NDUFS2 and NDUFA7 at the surface of complex I, and the buried C

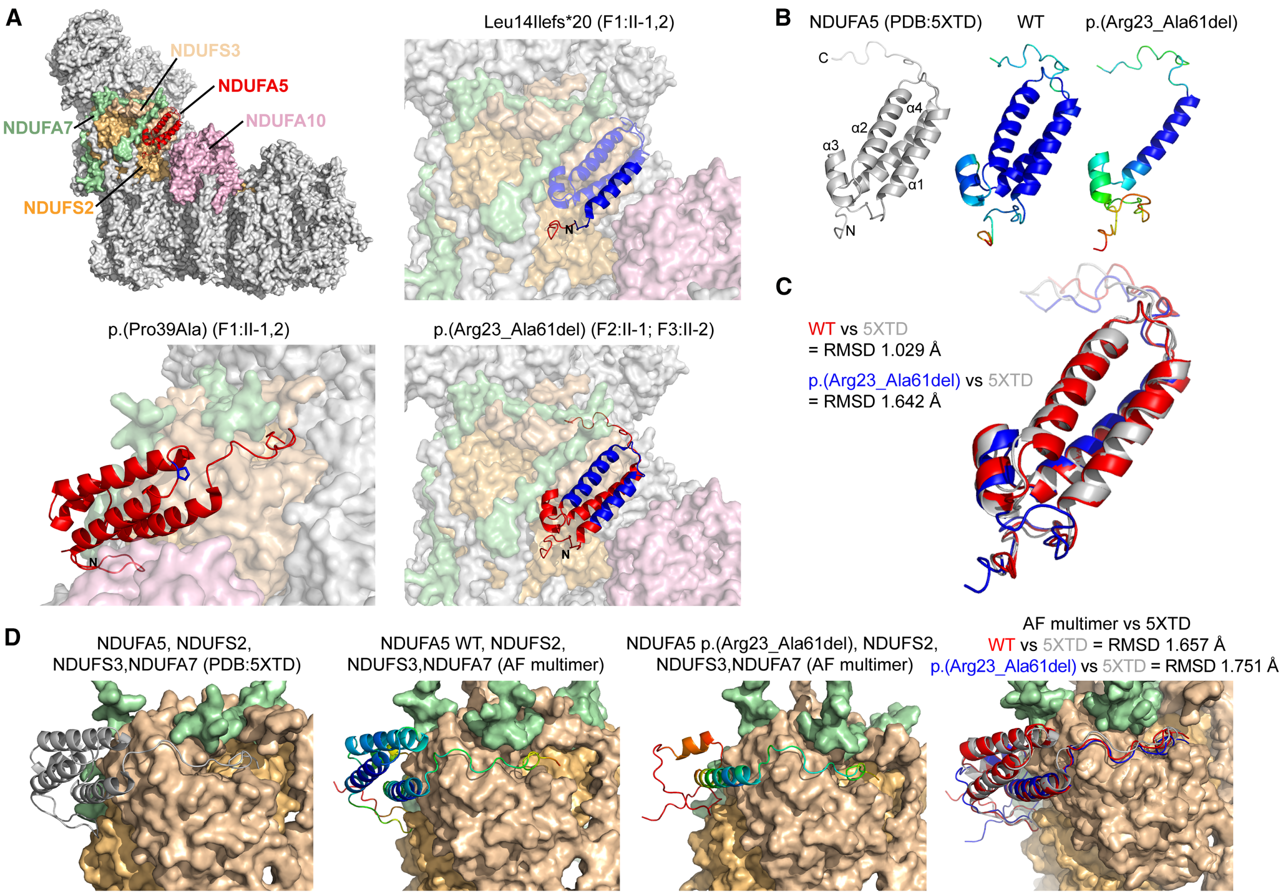

Figure 5. Molecular modeling
(A) Cryo-EM structure of the human complex I[7] (PDB: 5XTD) with NDUFA5 rendered in cartoon format. The blue regions of NDUFA5 in the subpanels indicate the deleted or substituted residues.
(B) Left: cartoon representation of human NDUFA5[7] with the alpha helices (α) numbered as indicated. Middle and right: AlphaFold2[20] models of WT and Arg23_Ala61del NDUFA5 colored according to pLDDT (red, low confidence; blue, high confidence).
(C) Alignment of structures in (B) using the *super* algorithm with the RMSD of WT (red) and Arg23_Ala61del (blue) NDUFA5 aligned to the experimentally observed protein (gray) as indicated.
(D) Left: experimentally determined[7] structure (extracted from PDB: 5XTD) for the subcomplex of NDUFA5 (gray), NDUFS2, NDUFS3, and NDUFA7 (subunits are colored as in A). Middle: AlphaFold-multimer[21] models of subcomplex containing WT or Arg23_Ala61del NDUFA5 colored according to pLDDT. Right: RMSD of the WT (red) or Arg23_Ala61del (blue) containing subcomplexes aligned to the experimentally determined structure.

terminus that interacts with NDUFS3 (Figure 5A, top right). Previous studies have shown a high co-dependence between NDUFA5 and NDUFS3, with the partner subunit also degraded in fibroblasts from individuals with *NDUFS2* variants[15] and a HEK293T *NDUFA5* knockout model[3] leading to a severe complex I assembly defect stalled with assembly of the P_D-a and P_D-b (ND4 and ND5) modules. Given that NDUFA5 was not detected in PBMCs from both F1:II-1 and F1:II-2 (Figure 3D), it is likely that subsequent destabilization of NDUFS3 is deleterious to assembly of the Q module. A similar conclusion can be made for the p.Pro39Ala variant found compound with the nonsense variant in family 1, as removal of the Pro39 residue likely results in destabilization of the α1 helix-turn-α2 helix motif (Figure 5A, bottom left).

F2:II-1 and F3:II-2 share the in-frame deletion variant, p.Arg23_Ala61del, with F2:II-1 being compound heterozygous with the start loss c.1A>C variant, while F3:II-2 is homozygous for the in-frame deletion variant (Figure 1A). The p.Arg23_Ala61del variant removes most of α helices 1 and 2 (Figure 5A, bottom right). Given there is no co-dependency between NDUFA10 and NDUFA5 in HEK293T knockout models,[3] we suppose that loss of this interaction is not deleterious. However, p.Arg23_Ala61del results in loss of the α2-NDUFS2 interaction and potentially the interactions with NDUFS2, NDUFS3, and NDUFA10 through helices 3 and 4 and the proteins at the C terminus. Given that NDUFA5 is produced and present in F3:II-2 (Figure 3D), who is homozygous for p.Arg23_Ala61del and whose clinical presentation is less severe than the other three individuals in this cohort, we supposed that loss of the α1 helix-turn-α2 helix motif in NDUFA5 is tolerated. To test this, we modeled the wild-type (WT) and Arg23_Ala61del sequence of NDUFA5 using ColabFold (AlphaFold2)[20,22] with AMBER relaxation enabled to resolve steric clashes. The model with the highest predicted local distance

difference test (pLDDT) confidence score was selected for analysis (Figure 5B). The AlphaFold-derived model of WT NDUFA5 was aligned to the experimentally determined structure of the human protein[19] using the *super* algorithm (structure-based dynamic programming alignment) yielding a root-mean-square deviation (RMSD) of 1.029 Å, demonstrating that AlphaFold can model the structure of NDUFA5 with good accuracy (Figure 5C). Alignment of Arg23_Ala61del NDUFA5 against the experimentally determined structure yielded an RMSD of 1.642 Å, with the core fold including α helices 3 and 4 preserved. To assess the structural stability of NDUFA5 in complex with its interactors, we modeled a complex of NDUFS2, NDUFS3, NDUFA7, and NDUFA5 using AlphaFold-multimer[21,22] (Figure 5D). Alignment of the complex containing WT NDUFA5 against the experimentally observed complex[19] had a core RMSD of 1.657 Å (Figure 5D, right), demonstrating that AlphaFold-multimer can model the Q-module structure with reasonably high accuracy. Crucially, the Arg23_Ala61del-containing model maintained this structural integrity, with a core RMSD of 1.751 Å, indicating that the deletion likely does not perturb the stability of the complex and suggesting that the interactions between the NDUFA5 α4 helix and C terminus with NDUFS2, NDUFS3, and NDUFA7 are retained.

Finally, we investigated the function of *ndufa5*, the zebrafish ortholog of human *NDUFA5*, during early development. Using CRISPR-Cas9 genome editing, we generated *ndufa5* F0 mutants (crispants) and evaluated their phenotypic consequences through morphological, behavioral, and electrophysiological assessments at 5–6 days post fertilization (dpf). Compared to Cas9-injected controls, *ndufa5* crispants exhibited notable developmental abnormalities. Morphological analysis revealed a significant reduction in eye distance (Figures 6A and 6B; unpaired *t* test, $p = 0.0045$) and body length ($p = 0.0037$), while the ratio of eye distance to body length remained unchanged ($p = 0.4431$), indicating proportional growth retardation. Additionally, *ndufa5* crispants displayed increased dark pigmentation relative to Cas9 controls. Behavioral analyses of 5-dpf larvae demonstrated significant impairments in spontaneous locomotor activity, with *ndufa5* crispants showing reduced total distance moved and maximum velocity (Figures 6C–6E; unpaired *t* test, $p = 0.0063$ and $p < 0.0001$, respectively). Furthermore, during light-dark stimulus paradigms, *ndufa5* crispants exhibited markedly diminished locomotor responses during dark intervals, as reflected by significantly decreased distance moved ($p < 0.0001$) and maximum velocity ($p = 0.0249$), whereas no significant differences were observed under light conditions ($p = 0.4650$ for distance moved, $p = 0.1016$ for maximum velocity) (Figures 6F and 6G). Survival analysis revealed a significantly reduced survival rate in *ndufa5* crispants compared to Cas9 controls (Figure 6H; log-rank test, $p < 0.0001$), with none of the *ndufa5* crispants surviving beyond 15 dpf and more than half succumbing by 6 dpf. While electrophysiological recordings of Cas9-injected controls (30/30) exhibited only baseline neural activity (Figure 6I), spontaneous epileptiform activity, including ictal-like and interictal-like discharges, were observed in *ndufa5* crispants (5/30) (Figure 6J). Collectively, these findings demonstrate that *ndufa5* is critical for normal embryonic development and neurological function in zebrafish larvae.

Aside from a recent report of a single individual,[23] variants in *NDUFA5* have not been associated with a human mitochondriopathy. A previous study utilizing TALEN and CRISPR-Cas9 gene-editing tools to disrupt *NDUFA5* in HEK293T cells demonstrated a convincing complex I deficiency via this knockout assay.[3] As such, there was a robust *a priori* hypothesis that NDUFA5 dysfunction could give rise to a complex I deficiency phenotype in humans.

The most common clinical conditions associated with mitochondrial complex I dysfunction are Leigh syndrome, Leber hereditary optic neuropathy (LHON; MIM: 535000), mitochondrial encephalomyopathy with lactic acidosis and stroke-like episodes (MELAS; MIM: 540000), myoclonic epilepsy with ragged-red fibers (MERRF; MIM: 545000), fatal infantile lactic acidosis, hypertrophic cardiomyopathy, and early-onset neurodegenerative disorders. Individuals frequently present with neurological symptoms such as encephalopathy, seizures, ataxia, dystonia, and psychomotor regression as well as myopathy, exercise intolerance, and lactic acidosis. Multisystem involvement is common, affecting the nervous system, skeletal muscle, heart, liver, and kidneys. Vision loss due to optic nerve degeneration is characteristic of LHON. The clinical spectrum is highly variable, with presentations ranging from isolated organ involvement to severe multisystem disease with early childhood onset and high mortality.[1,12,24–28] In addition to *NDUFA5*, other nuclear genes encoding non-catalytic subunits of complex I have been identified to be associated with mitochondrial disorders, including Leigh syndrome or isolated optic neuropathy.[10,11,28,29]

The multisystem phenotype seen for each individual in this cohort warranted genomic sequencing as the comprehensive genetic investigation of choice for each family. The most suspicious candidate variants led to the identification of bi-allelic variants in *NDUFA5* and the amalgamation of the three reported families into a single cohort through GeneMatcher.[12,26,30–32] Each family was ascertained from a different country, with individuals coming to medical attention at different time points throughout the last two decades. As such, genetic and functional testing has been necessarily varied between individuals depending on technological accessibility in their country of origin. The testing centers for each of the three families independently confirmed a complex I deficiency in all affected individuals, incorporating the detection of DNA variants, aberrant RNA phenotypes, and reduced mitochondrial complex I proteins in three different tissues. In an additional model, we show that NDUFA5 deficiency in zebrafish impairs embryonic and neural development.

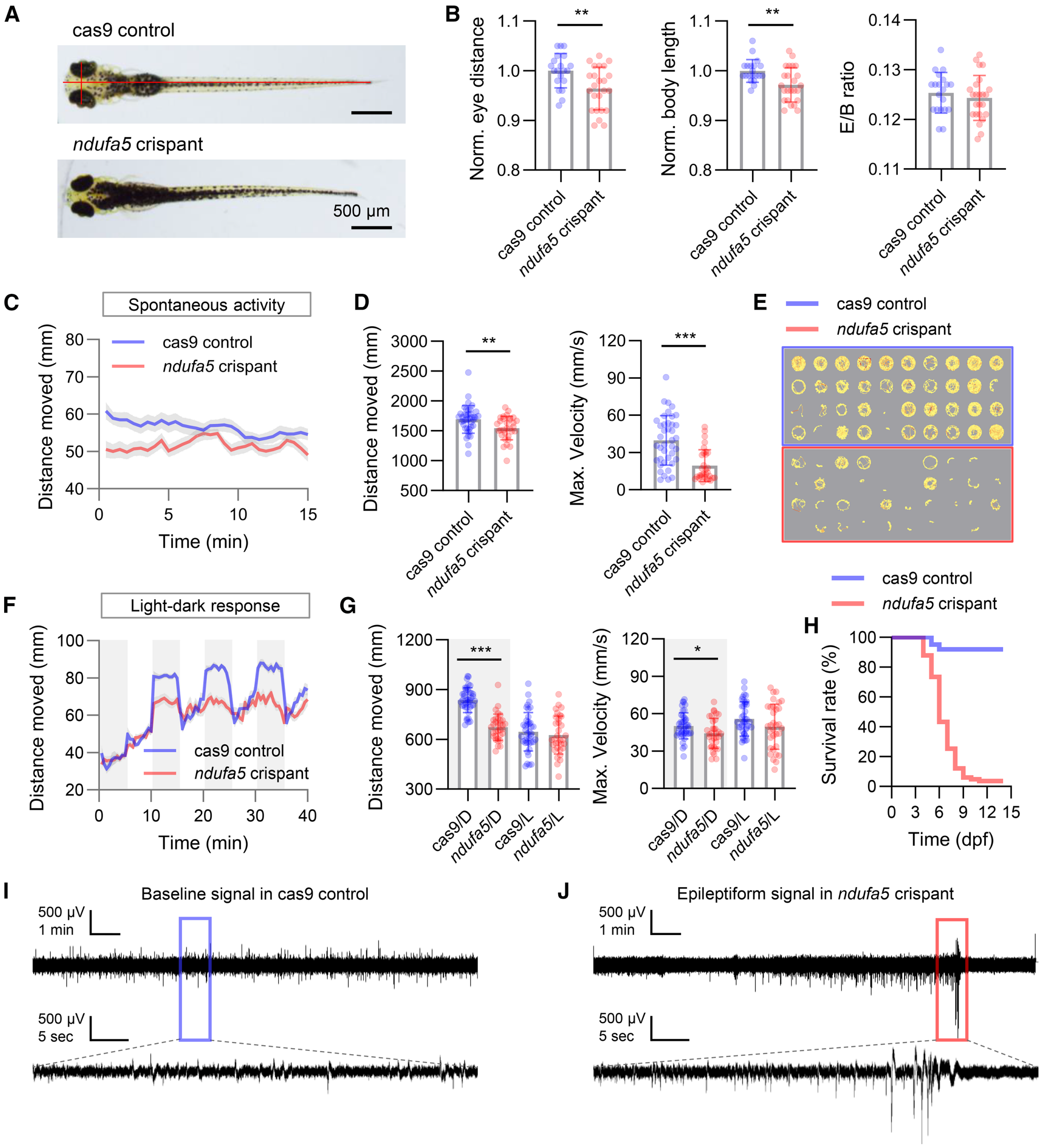

Figure 6. Disruption of zebrafish *ndufa5* results in abnormal embryonic development and neurodevelopmental deficits
(A) Representative brightfield images of 5-dpf larval zebrafish (dorsal view). Top: cas9-injected control; bottom: *ndufa5* crispant. Red lines denote eye distance and body length.
(B) Quantification of eye distance, body length, and eye distance/body length (E/B) ratio in cas9 controls ($n = 20$ fish) versus *ndufa5* crispants ($n = 24$ fish). Data are normalized to the mean of the cas9 control group.
(C) Spontaneous swim activity measured by distance moved for cas9 controls ($n = 40$ fish) and *ndufa5* crispants ($n = 31$ fish).
(D) As for (C) but quantification of total distance moved and maximum velocity.
(E) As for (C) but locomotion traces over 15 min of recording.
(F) Light-dark evoked response measured by distance moved for cas9 controls ($n = 40$ fish) and *ndufa5* crispants ($n = 33$ fish); gray blocks indicate dark periods.
(G) As for (F) but quantification of total distance moved and maximum velocity across dark-light epochs.
(H) Survival curves for cas9 controls ($n = 62$ fish) and *ndufa5* crispants ($n = 83$ fish).
(I) Representative local field potential (LFP) recordings from cas9 controls, showing baseline activity (magnified view below blue box).
(J) As for (I) but from *ndufa5* crispants, showing epileptiform events (magnified view below red box).
Scale bars are indicated in the figure. Error bars represent SEM for distance moved plots and SD for quantification plots. Statistical significance is indicated as $*p < 0.05$, $**p < 0.01$, and $***p < 0.001$.

In combination, we provide convincing evidence that *NDUFA5* deficiency results in a multisystemic mitochondrial disorder.

Infants and young children are most frequently affected by mitochondrial complex I dysfunction. The majority of cases present before the age of 3 years, with onset in

infancy being typical for the most severe phenotypes, including Leigh syndrome and fatal infantile lactic acidosis.[12,26,30–32] Adolescents and adults can also be affected, but this is less common and usually associated with specific syndromes such as LHON, which most often presents in young adults (median age of onset around 24 years), and occasionally with MELAS or MERRF, which may have variable age of onset but are still more common in childhood or adolescence.[1,33] Congenital heart disease is rare in mitochondrial disorders but has been reported in other individuals with complex I deficiency and other mitochondrial disorders.[34] As the congenital cardiac malformations seen for the affected siblings in family 1, namely coarctation of the aorta and atrial septal defects, are not well-recognized features of mitochondrial conditions, specific attention was paid to the analysis of virtual gene panels covering aortopathy conditions and congenital heart disease, neither search of which identified variants of clinical significance. It should be noted that the cardiac arrhythmia for F1:II-1 and cardiac hypertrophy for F1:II-2 are phenotypes appreciated in mitochondrial-related cardiac disease. Furthermore, these siblings from family 1 are the only individuals in this small cohort to present with congenital hematological abnormalities, a variable manifestation that is seen across many primary mitochondrial disorders.[35,36] Cytogenetic and flow-cytometry testing of a bone marrow sample from F1:II-1 did not detect any underlying primary hematological malignancy or other molecular cause for the various cytopenias, which is in support of these hematological abnormalities being part of the unifying mitochondrial complex I deficiency. As such, these cardiac and hematological aspects of the siblings' phenotype may represent a higher degree of multisystem variability over that of the other two individuals in this cohort. Certainly, the identification of a larger number of individuals with bi-allelic variants in *NDUFA5* resulting in complex I deficiency is required to better understand the phenotypic spectrum of *NDUFA5* dysfunction.

Interestingly, *NDUFA5*gene is neither missense constrained (Z score 0.85; observed/expected [o/e] 0.76, range 0.62–0.93), nor predicted loss-of-function (pLoF) constrained (probability of LoF intolerance [pLI] 0; o/e 0.78, range 0.46–1.41) as per gnomAD v.4.1.0. While all four variants reported for this cohort are present in the various gnomAD datasets, all have variant allele frequencies that could be considered acceptable for carrier rates relating to a recessive monogenic condition. Further to this, none are present in a homozygous state. Significantly, there are no compound heterozygous or homozygous rare variant pairings for *NDUFA5* across all variant classes as per gnomAD v.2.1.1 variant co-occurrence data. Our zebrafish studies show that *ndufa5* is critical for normal embryonic development and is consistent with a reported embryonic lethality in *Ndufa5* knockout mice.[1] Taken together, it is a reasonable supposition that bi-allelic LoF variants in *NDUFA5* may also be incompatible with embryonic survival in humans. This is supported by the various bi-allelic variant pairings seen for our cohort, where no combination is predicted to result in complete ablation of NDUFA5 protein production but, rather, the prediction of generating aberrant protein products in each instance.

In conclusion, we provide convincing clinical and functional genomic evidence, together with a substantive animal model and molecular modeling, in support of a mitochondrial complex I deficiency syndrome due to bi-allelic variants in *NDUFA5*, thus adding to the molecular differential diagnoses for complex I mitochondriopathies. For those individuals in whom a complex I deficiency has been clinically suspected and/or biochemically confirmed, but a molecular diagnosis remains elusive, this report serves to encourage the reanalysis of existing data regarding the potential for an *NDUFA5*-related mitochondriopathy. The cohort reported herein also exemplifies the complex pathomechanisms that may be encountered in diagnostic genomics and the potential need for adjunctive transcriptomics and proteomics in service of variant pathogenicity to secure a molecular diagnosis.

Data and code availability

The raw genomic and proteomic data that support the findings of this study have not been deposited in a public repository due to human research ethics committee and institutional review board restrictions but are available, along with all other materials, from the corresponding authors on request. This project did not involve development of new code.

Acknowledgments

The authors acknowledge and thank the families who participated in this study. The biochemical analyses for family 2, including respiratory chain enzymology measurements and protein studies, were performed by our much-respected colleague Dr. Dagmar Hahn, who sadly passed away in 2016. This project was supported by grants from the National Health and Medical Research Council (NHMRC) to N.B.T. (GNT2005458 and GNT1079342) and D.A.S. (GNT2009732); the Medical Research Future Fund (MRFF) to D.A.S., D.R.T., and D.H.H. (MRF2016030) and D.A.S. and D.H.H. (NCRI000043); Rare Disease Now (RDNow) and Undiagnosed Diseases Program Victoria (UDP-Vic) to S.M.W.; BMFTR (German Federal Ministry of Research, Technology, and Space) through the German Center for Child and Adolescent Health (DZKJ), the German Network for Mitochondrial Disorders (mitoNET, 01GM1906A), and EJP RD project GENOMIT (01GM1920A and 01GM2404A) to H.P., T.K., and R.K.; and the European Union's Horizon 2020 research and innovation program project Recon4IMD (101080997) to H.P. T.K. is a member of the European Reference Network (ERN) for Rare Neurological Diseases (ERN-RND) and the ERN for Neuromuscular Diseases (EURO-NMD). The research conducted at the Murdoch Children's Research Institute (MCRI) was supported by the Victorian Government's Operational Infrastructure Support Program. The project involved the Rare Disease Flagship, which acknowledges financial support from the Royal Children's Hospital Foundation, the MCRI, Harbig Foundation, Paula Fox, Andrew and Geraldine

Buxton Foundation, and The Pierce Armstrong Foundation. We thank the Mito Foundation for equipment grants to D.A.S. and D.H.H. and the Bio21 Mass Spectrometry and Proteomics Facility (MMSPF) for the provision of instrumentation, training, and technical support.

Author contributions

Conceptualization, N.B.T., T.B., A.S., J.L., D.A.S., and H.P.; clinical assessment, N.B.T., M.C., M.R., M.G., T.B., M.W., T.K., and W.M.-F.; investigation, D.H.H., T.E.G., T. Sharma, K.M.B., T. Stait, R.K., A.S., J.L., J.W., and X.Q.; funding acquisition, N.B.T., S.M.W., D.H.H., D.A.S., and H.P.; supervision, S.M.W., D.R.T., J.L., K.M.B., D.A.S., and H.P.; writing – original draft, N.B.T., M.R., and D.A.S.; writing – review & editing, all authors.

Declaration of interests

Cipher Gene Chengdu Ltd. is a medical data development company that employs J.W., X.Q., and J.L. T.K. has received research support, speaker honoraria, consulting fees, and travel reimbursement from Santhera Pharmaceuticals, Chiesi GmbH, and GenSight Biologics.

Supplemental information

Supplemental information can be found online at https://doi.org/10.1016/j.ajhg.2026.03.003.

Web resources

GenBank, https://www.ncbi.nlm.nih.gov/genbank/
gnomAD, https://gnomad.broadinstitute.org/
OMIM, https://www.omim.org/

Received: October 9, 2025
Accepted: March 3, 2026
Published: March 30, 2026

References

1. Peralta, S., Torraco, A., Wenz, T., Garcia, S., Diaz, F., and Moraes, C.T. (2014). Partial complex I deficiency due to the CNS conditional ablation of Ndufa5 results in a mild chronic encephalopathy but no increase in oxidative damage. Hum. Mol. Genet. *23*, 1399–1412. https://doi.org/10.1093/hmg/ddt526.
2. Marui, T., Funatogawa, I., Koishi, S., Yamamoto, K., Matsumoto, H., Hashimoto, O., Jinde, S., Nishida, H., Sugiyama, T., Kasai, K., et al. (2011). The NADH-ubiquinone oxidoreductase 1 alpha subcomplex 5 (NDUFA5) gene variants are associated with autism. Acta Psychiatr. Scand. *123*, 118–124. https://doi.org/10.1111/j.1600-0447.2010.01600.x.
3. Stroud, D.A., Surgenor, E.E., Formosa, L.E., Reljic, B., Frazier, A.E., Dibley, M.G., Osellame, L.D., Stait, T., Beilharz, T.H., Thorburn, D.R., et al. (2016). Accessory subunits are integral for assembly and function of human mitochondrial complex I. Nature *538*, 123–126. https://doi.org/10.1038/nature19754.
4. Zhu, J., Vinothkumar, K.R., and Hirst, J. (2016). Structure of mammalian respiratory complex I. Nature *536*, 354–358. https://doi.org/10.1038/nature19095.
5. Fiedorczuk, K., Letts, J.A., Degliesposti, G., Kaszuba, K., Skehel, M., and Sazanov, L.A. (2016). Atomic structure of the entire mammalian mitochondrial complex I. Nature *538*, 406–410. https://doi.org/10.1038/nature19794.
6. Hirst, J. (2013). Mitochondrial complex I. Annu. Rev. Biochem. *82*, 551–575. https://doi.org/10.1146/annurev-biochem-070511-103700.
7. Gu, J., Wu, M., Guo, R., Yan, K., Lei, J., Gao, N., and Yang, M. (2016). The architecture of the mammalian respirasome. Nature *537*, 639–643. https://doi.org/10.1038/nature19359.
8. Letts, J.A., Fiedorczuk, K., and Sazanov, L.A. (2016). The architecture of respiratory supercomplexes. Nature *537*, 644–648. https://doi.org/10.1038/nature19774.
9. Zheng, W., Chai, P., Zhu, J., and Zhang, K. (2024). High-resolution in situ structures of mammalian respiratory supercomplexes. Nature *631*, 232–239. https://doi.org/10.1038/s41586-024-07488-9.
10. Fiedorczuk, K., and Sazanov, L.A. (2018). Mammalian Mitochondrial Complex I Structure and Disease-Causing Mutations. Trends Cell Biol. *28*, 835–867. https://doi.org/10.1016/j.tcb.2018.06.006.
11. Hock, D.H., Robinson, D.R.L., and Stroud, D.A. (2020). Blackout in the powerhouse: clinical phenotypes associated with defects in the assembly of OXPHOS complexes and the mitoribosome. Biochem. J. *477*, 4085–4132. https://doi.org/10.1042/BCJ20190767.
12. Fassone, E., and Rahman, S. (2012). Complex I deficiency: clinical features, biochemistry and molecular genetics. J. Med. Genet. *49*, 578–590. https://doi.org/10.1136/jmedgenet-2012-101159.
13. Swalwell, H., Kirby, D.M., Blakely, E.L., Mitchell, A., Salemi, R., Sugiana, C., Compton, A.G., Tucker, E.J., Ke, B.X., Lamont, P.J., et al. (2011). Respiratory chain complex I deficiency caused by mitochondrial DNA mutations. Eur. J. Hum. Genet. *19*, 769–775. https://doi.org/10.1038/ejhg.2011.18.
14. Sobreira, N., Schiettecatte, F., Valle, D., and Hamosh, A. (2015). GeneMatcher: a matching tool for connecting investigators with an interest in the same gene. Hum. Mutat. *36*, 928–930. https://doi.org/10.1002/humu.22844.
15. Hock, D.H., Caruana, N.J., Semcesen, L.N., Lake, N.J., Formosa, L.E., Amarasekera, S.S.C., Stait, T., Tregoning, S., Frajman, L.E., Bournazos, A.M., et al. (2025). Untargeted proteomics enables ultra-rapid variant prioritisation in mitochondrial and other rare diseases. Genome Med. *17*, 58. https://doi.org/10.1186/s13073-025-01467-z.
16. Dibley, M.G., Formosa, L.E., Lyu, B., Reljic, B., McGann, D., Muellner-Wong, L., Kraus, F., Sharpe, A.J., Stroud, D.A., and Ryan, M.T. (2020). The Mitochondrial Acyl-carrier Protein Interaction Network Highlights Important Roles for LYRM Family Members in Complex I and Mitoribosome Assembly. Mol. Cell. Proteomics *19*, 65–77. https://doi.org/10.1074/mcp.RA119.001784.
17. Guerrero-Castillo, S., Baertling, F., Kownatzki, D., Wessels, H.J., Arnold, S., Brandt, U., and Nijtmans, L. (2017). The Assembly Pathway of Mitochondrial Respiratory Chain Complex I. Cell Metab. *25*, 128–139. https://doi.org/10.1016/j.cmet.2016.09.002.
18. Formosa, L.E., Muellner-Wong, L., Reljic, B., Sharpe, A.J., Jackson, T.D., Beilharz, T.H., Stojanovski, D., Lazarou, M., Stroud, D.A., and Ryan, M.T. (2020). Dissecting the Roles of Mitochondrial Complex I Intermediate Assembly Complex

Factors in the Biogenesis of Complex I. Cell Rep. *31*, 107541. https://doi.org/10.1016/j.celrep.2020.107541.
19. Guo, R., Zong, S., Wu, M., Gu, J., and Yang, M. (2017). Architecture of Human Mitochondrial Respiratory Megacomplex I(2)III(2)IV(2). Cell *170*, 1247–1257.e12. https://doi.org/10.1016/j.cell.2017.07.050.
20. Jumper, J., Evans, R., Pritzel, A., Green, T., Figurnov, M., Ronneberger, O., Tunyasuvunakool, K., Bates, R., Žídek, A., Potapenko, A., et al. (2021). Highly accurate protein structure prediction with AlphaFold. Nature *596*, 583–589. https://doi.org/10.1038/s41586-021-03819-2.
21. Evans, R., O'Neill, M., Pritzel, A., Antropova, N., Senior, A., Green, T., Žídek, A., Bates, R., Blackwell, S., Yim, J., et al. (2021). Protein complex prediction with AlphaFold-Multimer. bioRxiv. https://doi.org/10.1101/2021.10.04.463034.
22. Mirdita, M., Schütze, K., Moriwaki, Y., Heo, L., Ovchinnikov, S., and Steinegger, M. (2022). ColabFold: making protein folding accessible to all. Nat. Methods *19*, 679–682. https://doi.org/10.1038/s41592-022-01488-1.
23. Wang, Y., Yang, X., Yan, X., Zhu, Y., Xia, X., Li, T., Liao, Y., Lei, B., Yang, J., and Li, D. (2025). Identification of respiratory chain complex I deficiency due to NDUFA5 variants as a novel cause of infantile fatal disease. Genes Dis. *13*, 101920. https://doi.org/10.1016/j.gendis.2025.101920.
24. Dar, G.M., Ahmad, E., Ali, A., Mahajan, B., Ashraf, G.M., and Saluja, S.S. (2023). Genetic aberration analysis of mitochondrial respiratory complex I implications in the development of neurological disorders and their clinical significance. Ageing Res. Rev. *87*, 101906. https://doi.org/10.1016/j.arr.2023.101906.
25. Distelmaier, F., Koopman, W.J.H., van den Heuvel, L.P., Rodenburg, R.J., Mayatepek, E., Willems, P.H.G.M., and Smeitink, J.A.M. (2009). Mitochondrial complex I deficiency: from organelle dysfunction to clinical disease. Brain *132*, 833–842. https://doi.org/10.1093/brain/awp058.
26. Ma, Y.Y., Li, X.Y., Li, Z.Q., Song, J.Q., Hou, J., Li, J.H., Sun, L., Jiang, J., and Yang, Y.L. (2018). Clinical, biochemical, and genetic analysis of the mitochondrial respiratory chain complex I deficiency. Medicine (Baltim.) *97*, e11606. https://doi.org/10.1097/MD.0000000000011606.
27. Barbosa-Gouveia, S., González-Vioque, E., Borges, F., Gutiérrez-Solana, L., Wintjes, L., Kappen, A., van den Heuvel, L., Leis, R., Rodenburg, R., and Couce, M.L. (2019). Identification and Characterization of New Variants in FOXRED1 Gene Expands the Clinical Spectrum Associated with Mitochondrial Complex I Deficiency. J. Clin. Med. *8*, 1262. https://doi.org/10.3390/jcm8081262.
28. Scheffler, I.E. (2015). Mitochondrial disease associated with complex I (NADH-CoQ oxidoreductase) deficiency. J. Inherit. Metab. Dis. *38*, 405–415. https://doi.org/10.1007/s10545-014-9768-6.
29. Magrinelli, F., Cali, E., Braga, V.L., Yis, U., Tomoum, H., Shamseldin, H., Raiman, J., Kernstock, C., Rezende Filho, F.M., Barsottini, O.G.P., et al. (2022). Biallelic Loss-of-Function NDUFA12 Variants Cause a Wide Phenotypic Spectrum from Leigh/Leigh-Like Syndrome to Isolated Optic Atrophy. Mov. Disord. Clin. Pract. *9*, 218–228. https://doi.org/10.1002/mdc3.13398.
30. Piekutowska-Abramczuk, D., Assouline, Z., Mataković, L., Feichtinger, R.G., Koňaříková, E., Jurkiewicz, E., Stawiński, P., Gusic, M., Koller, A., Pollak, A., et al. (2018). NDUFB8 Mutations Cause Mitochondrial Complex I Deficiency in Individuals with Leigh-like Encephalomyopathy. Am. J. Hum. Genet. *102*, 460–467. https://doi.org/10.1016/j.ajhg.2018.01.008.
31. Danhelovska, T., Kolarova, H., Zeman, J., Hansikova, H., Vaneckova, M., Lambert, L., Kucerova-Vidrova, V., Berankova, K., Honzik, T., and Tesarova, M. (2020). Multisystem mitochondrial diseases due to mutations in mtDNA-encoded subunits of complex I. BMC Pediatr. *20*, 41. https://doi.org/10.1186/s12887-020-1912-x.
32. Tuppen, H.A.L., Hogan, V.E., He, L., Blakely, E.L., Worgan, L., Al-Dosary, M., Saretzki, G., Alston, C.L., Morris, A.A., Clarke, M., et al. (2010). The p.M292T NDUFS2 mutation causes complex I-deficient Leigh syndrome in multiple families. Brain *133*, 2952–2963. https://doi.org/10.1093/brain/awq232.
33. Vacchiano, V., Caporali, L., La Morgia, C., Carbonelli, M., Amore, G., Bartolomei, I., Cascavilla, M.L., Barboni, P., Lamperti, C., Catania, A., et al. (2021). The m.3890G>A/MT-ND1 mtDNA rare pathogenic variant: Expanding clinical and MRI phenotypes. Mitochondrion *60*, 142–149. https://doi.org/10.1016/j.mito.2021.08.007.
34. Mastantuono, E., Wolf, C.M., and Prokisch, H. (2019). Genetic Basis of Mitochondrial Cardiomyopathy. In Genetic Causes of Cardiac Disease, J.M. Erdmann, ed. (Alessandra: Springer), pp. 93–139. https://doi.org/10.1007/978-3-030-27371-2.
35. Finsterer, J. (2007). Hematological manifestations of primary mitochondrial disorders. Acta Haematol. *118*, 88–98. https://doi.org/10.1159/000105676.
36. Finsterer, J., and Frank, M. (2015). Haematological abnormalities in mitochondrial disorders. Singapore Med. J. *56*, 412–419. https://doi.org/10.11622/smedj.2015112.

The American Journal of Human Genetics, Volume *113*

Supplemental information

Bi-allelic variants in *NDUFA5* cause a mitochondriopathy with complex I deficiency

Natalie B. Tan, Matthias Gautschi, Michael Raum, Daniella H. Hock, Robert Kopajtich, Jia Wang, Xiao Qian, Tanavi Sharma, Timothy E. Green, Jean-Marc Nuoffer, Katrina M. Bell, Katarzyna Pospieszny, Tegan Stait, Chloe Pike, Michelle Cao, Susan M. White, David R. Thorburn, Theresa Brunet, Matias Wagner, Wolfgang Müller-Felber, Leopold Zeng, Thomas Klopstock, André Schaller, Jing Liu, David A. Stroud, and Holger Prokisch

Supplemental notes: Case Reports

Family 1

Individual F1:II-1 is the first-born child to non-consanguineous parents of Korean descent. Both parents are generally healthy and well, with no known family history of underlying monogenic conditions. Combined first trimester screening returned a low-risk result for the common aneuploidies of trisomy 21, 13 and 18. Morphology ultrasound at 20-weeks' gestation detected a cardiac anomaly that was investigated via dedicated fetal echocardiogram at 21+2 weeks' gestation, demonstrating a hypoplastic aortic arch with left to right heart size discrepancy. Concerns for prenatal growth restriction were monitored via fortnightly growth scans from 32-weeks' gestation, with an estimated fetal weight on the second centile reported by 36-weeks. Amniocentesis for five probe FISH (13, 18, 21, X and Y) and SNP microarray both returned normal results. F1:II-1was born at 36+6 weeks' gestation via a planned Cesarean section owing to the cardiac anomalies and anticipated need for tertiary neonatal cardiac care. He was born in good condition, requiring no resuscitation, with a birth weight of 2120 grams. Postnatal echocardiogram confirmed a coarctation of the aorta with atrial septal defect for which surgical repair was conducted on day 8 of life. Significantly, F1:II-1's post-operative cardiac recovery was complicated by sinus node dysfunction that manifested with a chaotic atrial rhythm that has self-resolved. He was re-admitted to hospital at 4 months of age with increased respiratory effort, worsening feeds and poor weight gain due to persistent pulmonary arterial hypertension secondary to elevated biventricular end diastolic pressures.

Hyperlactatemia, hyperglycemia and hypocalcemia were noted at birth, with the latter two issues resolving quickly with routine management. The hyperlactatemia persisted for the first six months of life. Alongside the antenatal diagnoses of coarctation of the aorta and intrauterine growth restriction, F1:II-1 had persisting postnatal growth restriction with head circumference preservation, hypospadias for which surgery is planned, a sacral dimple with no associated hair tufting and normal spinal ultrasound, dysmorphism that included a large anterior fontanelle with metopic ridging, and congenital generalized hirsutism with merging of the temporal hairline and lateral margins of the eyebrows. The hirsutism resolved across the first months of life. F1:II-1 also has a striking hematological phenotype, with pancytopenia (anemia, thrombocytopenia, leukopenia) detected on the initial full blood count taken at birth. He had a congenital red cell macrocytosis that required multiple red cell transfusions, the first of which occurred on day 12 of life. He continues to experience a persistent but fluctuating neutropenia and lymphopenia. Owing to the clinical suspicion for an underlying bone marrow failure condition, testing of a bone marrow sample was conducted via G-banded karyotype, FISH and flow cytometry, all of which returned uninformative results. The examination of the bone marrow aspirate sample identified the presence of ring sideroblasts and

vacuolated erythroid precursors, which can be seen in mitochondrial disorders. Whilst early developmental concerns were raised in the context of recovery from a complicated neonatal course, F1:II-1 has demonstrated gradual catch-up to largely age-appropriate milestones by age 2 years.

Owing to the complex constellation of medical issues for F1:II-1 at birth, he was investigated for the possibility of an underlying metabolic condition, returning normal results for urine metabolic screening and urinary organic acids, plasma amino acids, free and total carnitine, and chromosome breakage studies. Brain MRI at three weeks of age revealed mildly prominent ventricles only. Clinical trio genome sequencing was requested for F1:II-1 at 15 days of age which was uninformative.

Individual F1:II-2 is the second child born to the same Korean parents. Similar to F1:II-1, she was diagnosed antenatally with congenital heart disease involving a tortuous aortic arch, mildly small left sided structures, and mild biventricular hypertrophy with preserved systolic function, with postnatal echocardiogram also confirming a bicuspid aortic valve, small fenestrated atrial septal defect, and mild left pulmonary artery stenosis; all of which have been conservatively managed. She was also born at 36+6 weeks' gestation via planned Cesarean section in the same context of intrauterine growth restriction that persisted to postnatal growth restriction. Once again, much like her brother, she had neonatal lactatemia and congenital hirsutism that spontaneously resolved, as well as a sacral dimple with normal spinal ultrasound. F1:II-2 has had normal development to age 6 months at the time of last assessment. The hematological phenotype for F1:II-2 mimics a very similar course to that of her brother, with a congenital macrocytic anemia requiring red cell transfusion at 1 month of life, together with persistent neutropenia and lymphopenia.

Given the striking phenotypic overlap between siblings, clinical singleton genome sequencing was also arranged for F1:II-2 shortly after birth and unsurprisingly returned an uninformative result when analyzed together with her parents' existing genome sequencing data. The family was subsequently enrolled into a rare disease research program where quad WGS analysis identified compound heterozygous variants of interest in *NDUFA5* that were shared by these siblings. The maternally inherited missense variant, p.(Pro39Ala), affects a highly conserved nucleotide with moderate to deleterious *in silico* predictions (see **Table 1**). The variant is ultra-rare in gnomAD v4.1.0 with no homozygotes or alternative missense substitutions for Pro39. The paternally inherited frameshift variant, p.(Leu14Ilefs*20), is predicted to introduce a premature termination codon at nucleotide positions 100-102 after the transcription start site. Once again, the variant is ultra-rare in gnomAD v4.1.0 with no reported homozygotes.

Family 2

F2:II-1 is the first child born to non-consanguineous parents of Swiss ancestry. There is a family history of cystic fibrosis in F2:II-1's younger sister. F2:II-1 was born at 42-weeks' gestation via Cesarean section with Apgar scores of 6, 7 and 9 at 1, 5 and 10 minutes respectively. After an initial period of normal development, he presented at 10 months with new-onset horizontal nystagmus, mild grasp asymmetry and instability whilst sitting. This initial presentation was attributed to parainfectious cerebellitis in the context of an intercurrent enterovirus infection. There was normal to mildly increased lactate (max. 3.6 mmol/L). Brain MRI (see **Figure 1C**) and MRS were performed one month later which demonstrated symmetric focal T2 signal alterations corresponding to diffusion restriction on DWI in the subthalamic region of the transition from the capsula interna to the pedunculus cerebri, as well as in the dorso-medial pons on both sides, together with increased patchy lactate peaks in the frontoparietal white matter, all findings suggestive of an underlying mitochondrial disorder. As such, respiratory chain enzyme studies were arranged on skeletal muscle biopsy and skin fibroblasts, which both showed an isolated Complex I deficiency (see **Table 2**). Reduced Complex I activity was more pronounced in skeletal muscle compared to F2:II-1 fibroblasts. In skeletal muscle the residual Complex I activity was 10.5% compared to controls (expressed as activity ratios CI/CS: F2:II-1 0.02 (range 0.014- 0.028) normal range 0.19 +/- 0.04). The residual Complex I activity in F2:II-1 fibroblasts was 34% (expressed as activity ratios CI/CS: F2:II-1 0.10 (range 0.19-0.46) normal range 0.29 +/- 0.06). These findings were supportive of a mitochondrial disorder with a profound Complex I deficiency, however genetic testing at this time did not identify a known underlying molecular cause. A mitochondrial cocktail of carnitine, riboflavin, tocopherol and coenzyme Q10 was instituted but with no notable effect, other than the correction of a low plasma coenzyme Q10 level.

By one year of age, F2:II-1 displayed generalized muscular hypotonia with mild psychomotor delay and progressive mild microcephaly. At 14 months, walking whilst holding onto walls was possible. At age 2 years he was speaking in 2-word phrases but was not ambulating due to ataxia, and whilst the nystagmus had ceased, there was now progressive bilateral external ophthalmoplegia noted, with ocular nystagmus in the context of bilateral optic neuropathy. F2:II-1 went on to experience intermittent periods of motor developmental regression in the context of viral-induced respiratory deterioration with abundant secretion production. He also developed recurrent supraventricular tachycardia in light of a pre-excitation syndrome, together with predominantly left-sided hypertrophic cardiomyopathy which remained stable on serial follow-up. Gastrotomy feeding was commenced at 2.5 years of age due to increased feeding difficulties and failure to thrive.

A ketogenic diet was introduced at 3.5 years of age and well tolerated, with perceived slowing of disease progression, however he then developed a severe sleeping disorder. Cognitive development was normal, if not above average, with non-verbal IQ assessed between 114-137. There was continued slow deterioration of motor function over the following years, with increasing myopathic stiffness to the point of a loss of the majority of his motor skills. F2:II-1 encountered severe anxiety in his later years, making the sleepless nights intolerable at which point a rocking bed was arranged at 11 years of age to simulate the movement of a night train. This intervention considerably improved sleep, motor and cognitive functions.[1] After use of the bed was withdrawn for several months, F2:II-1 experienced an acute deterioration and passed away shortly after his 13th birthday. The family was then included in a trio WGS project for unsolved cases, which identified compound heterozygous candidate variants in *NDUFA5*.

The maternally inherited missense variant, c.1A>C;p.0? affects a highly conserved nucleotide in the start codon and therefore predicts a start-loss variant. *In silico* prediction tools consider this variant as deleterious or of uncertain significance (see **Table 1**). The variant is ultra-rare in gnomAD v.4.1.0 with no reported homozygotes. The paternally inherited variant c.183G>A is synonymous p.(Ala61=) but concerns the last nucleotide of exon 3 and therefore the adjacent splice donor site. Despite this, *in-silico* prediction did not predict a high probability of a mis-splicing event (SpliceAI acceptor loss = 0.12, donor loss = 0.06) (see **Table 1**). The variant is ultra-rare in gnomAD v.4.1.0 with no reported homozygotes.

Family 3

F3:II-2 was born to second-cousin consanguineous parents of Turkish ancestry. The 17-year-old sister, both parents, and the 7-year-old brother are clinically unaffected. On the maternal side, there is a history of two miscarriages in the third-to-fifth months of pregnancy, as well as two neonatal deaths. The maternal grandfather has a history of migraine headaches.

Prenatal and perinatal development were unremarkable. At 10 months of age, F3:II-2 experienced a febrile seizure with a normal EEG recorded at this time. At 2.5 years, early neurological symptoms emerged including nystagmus, bilateral optic atrophy, and gait instability. A markedly elevated lactate level (6.1 mmol/l) raised suspicion for mitochondrial dysfunction. By the age of three years, a cranial MRI was performed that revealed two oval-shaped hyperintensities in the substantia nigra, along with a subtle band-like signal increase in the periaqueductal region (see **Figure 1D**). The differential diagnosis at this stage included Leigh syndrome. Biochemical examination of individual mitochondrial energy metabolism enzymes from muscle showed a reduction of Complex I activity

relative to the marker enzyme citrate synthase (see **Table 2**). Other enzyme complexes (Complex II–IV) were within normal limits. These findings confirmed a mitochondriopathy with Complex I deficiency.

Due to bilateral cavus foot deformity with hallux valgus, Achilles tendon lengthening was performed at the ages of 10, 12, and 14 years. Initial singleton exome sequencing at age 16 years was inconclusive. F3:II-2 showed continued neurological decline. By age 18 years, she was no longer able to walk independently and required both a wheelchair and a rollator. Bilateral optic atrophy and gaze-evoked nystagmus persisted, while pupillary responses and fundoscopy remained normal. Ophthalmological examination revealed significantly reduced visual acuity and atrophy of the ganglion cell layer on optical coherence tomography. Visual acuity was stable when compared with previous examinations, and the ganglion cell layer atrophy had also been documented during previous pediatric inpatient evaluation. Cognitive function and mood were preserved. Notably, no neutropenia or cardiac phenotype were observed for F3:II-2.

Reanalysis of the original singleton exome sequencing data ultimately identified a homozygous synonymous variant in the *NDUFA5* gene. This is the same c.183G>A variant identified in F2:II-1 which impacts the last nucleotide of exon 3 and the splice donor site. Following the diagnosis of Leigh syndrome, based on clinical phenotype, MRI findings, muscle biopsy, and genetic testing, a therapeutic trial with 900 mg/day idebenone was initiated at 19 years of age. During regular follow-up visits every three months, visual acuity and possible side effects were monitored. The therapy was well tolerated but so far has not led to measurable clinical improvement in visual acuity.

Supplemental Materials and Methods

All *NDUFA5* variants were identified via genomic sequencing. Phenotype data were collected via direct assessment by study co-authors and medical record review. All procedures were followed in accordance with the ethical standards of the responsible committee on human experimentation. Informed consent was obtained for each study participant.

DNA Sequencing

For Family 1 (F1), whole genome sequencing (WGS) was performed using massively parallel sequencing (Nextera^{TM} DNA Flex Library Prep kit, Illumina) with a mean target coverage of 30x and a minimum of 90% of bases sequenced to at least 10x for nDNA and a minimum of 800x mean coverage for mtDNA. Data were processed, including read alignment to the reference genome (GRCh38) and the revised Cambridge Reference Sequence (rCRS) mitochondrial genome

(NC_012920.1). Variant calling for nDNA was performed using Cpipe,[2] or the functionally equivalent analysis with the Illumina Dragen System. For mtDNA, variants were called using Mutect2. Automated sex determination, relatedness and contamination checks were performed. For nDNA, variant analysis and interpretation within the selected target region (RefSeq genes +/- 1kb) was performed using Agilent Alissa Interpret. Variants were annotated against all RefSeq gene transcripts and reported in accordance with HGVS nomenclature. Genomic coordinates were generated by Cpipe and do not necessarily comply with HGVS guidelines. Copy number variants (CNVs) were screened for using an internal CNV detection tool, CXGo.[3] For mtDNA, a custom in-house analysis pipeline was used to detect large deletions and annotate the VCF file with variant information. Genes with proven disease association are considered during routine analysis. Curation of nDNA variants was phenotype-driven with pre-curated or custom gene lists used for variant prioritization. Since no likely cause of disease was identified in the Mendeliome, other candidate variants were considered.

For Family 2 (F2), DNA from venous peripheral blood was extracted using a Maxwell RSC 48 instrument (Promega). DNA concentration was measured using QuantiFluor Dyes on a Quantus Fluorometer (Promega) as described by the manufacturer. DNA was fragmented on a Covaris with targeted size of 350 bp. Subsequently, the TruSeq DNA PCR-Free Kit (Illumina) was used to prepare DNA libraries for whole genome sequencing according to the manufacturer's protocol with 1 µg DNA input. DNA libraries were subsequently sequenced on a NovaSeq 6000 (Illumina) as 150 bp paired ends with an average coverage of approximately 40x. Alignment of sequencing reads was to the human reference genome GRCh37/hg19. Variant calling and annotation were performed using VarSome Clinical (Saphetor SA, Lausanne, Switzerland). Copy number variants (CNVs) were analyzed using ExomeDepth integrated in the VarSome Clinical suite.

For Family 3 (F3), exome sequencing was performed as part of the GENOMIT project (https://www.genomit.eu), as previously described.[4] Briefly, genomic DNA was extracted from whole blood and fragmented following standard protocols. Target enrichment was performed using the Twist Exome 2.0 kit and sequencing was conducted on an Illumina NovaSeq 6000 platform in 100 bp paired-end mode, achieving an average coverage greater than 75× with 98.1% of the target region covered >20×. Sequence reads were aligned to the human reference genome GRCh37/hg19 using the Burrows-Wheeler Aligner (BWA). Variant calling for single nucleotide variants (SNVs) was performed with the Genome Analysis Toolkit (GATK v4.2.3.0), while insertions and deletions (indels) up to 20 bp were identified using Pindel. Copy number variants (CNVs) and structural variants (SVs) were detected through an integrative approach combining ExomeDepth, Manta, LUMPY, BreakDancer, and WHAMG. Data analysis and interpretation were conducted using the

open-source next-generation sequencing (NGS) analysis and clinical decision-support platform EVAdb (LIMS Production version 2022.05; https://github.com/mri-ihg/EVAdb). Variants in *NDUFA5* were confirmed by RNA sequencing (RNAseq) data.

RNA studies

RNA sequencing data for F1:II-1 and controls were mapped to the hg38 reference genome with the gap aware aligner program, STAR V2.7.3a. The 2-pass method within STAR[5] was utilized, with gencode.v41 gene annotation to enhancer mapping and the detection of unique splicing events. Quality of data was assessed with FastQC. Specific splicing pattern of the *NDUFA5* gene was observed using IGV.[6]

For F2:II-1, total RNA from control and F2:II-1 fibroblasts was isolated using the QIAGEN RNeasy Kit according to the manufacturer's instructions. Random oligohexamer primed RNA was reverse transcribed using the SuperScript II First-Strand cDNA Synthesis System (Invitrogen) according to the manufacturer's recommendations. One-twenty-fifth of single stranded cDNA were used as a template to amplify *NDUFA5* cDNA using gene specific oligonucleotides located in the exon 1-2 junction (forward primer: NDUFA5_Ex1-2_F 5'-GGGTGTGCTGAAGAAGACCACTG-3' and in exon 4 (reverse primer NDUFA5_Ex_4_R 5'- TTCTAATTGACCGCCTTGAAG-3'), respectively. Products were resolved on 1.2% (w/v) Agarose gel electrophoresis. Additionally, all RT-PCR fragments were sequenced using BigDye chemistry on an ABI3730XL genetic analyzer.

Strand-specific RNAseq of F3:II-2 and control fibroblast samples was performed using the TruSeq Stranded mRNA Sample Prep LS protocol (Illumina). [7,8] Data processing and analysis for F3:II-2 were carried out using the DROP pipeline, as described by Yepez et al.[9] For outlier detection, the sample was analyzed alongside a cohort of similarly processed in-house samples (total n = 758), following the approach detailed by Kopajtich et al.[10] and Yepez et al.[11]

Proteomics

Quad analysis of proband F1:II-1, affected sibling F1:II-2 and parental (F1:I-1, F1:I-2) PBMCs was performed as previously described.[12] Briefly, PBMCs were isolated from approximately 3 mL EDTA blood from adults and six unrelated age matched adult controls and 1-2 mL from proband, F1:II-2 and five age matched controls using SepMate columns (StemCell Technologies) according to manufacturer's instructions. PBMC pellets were resuspended in a lysis buffer (5 % SDS [w/v], 50 mM triethylammonium bicarbonate) and protein concentration estimated using the Pierce BCA Protein Assay Kit (Thermo Scientific). A total of 20 μg of protein was digested (in triplicate for

proband, sibling and parents, and single replicate for controls) using S-Trap micro columns (ProtiFi) according to the manufacturer's instructions at a 1:20 trypsin to protein ratio as previously described. Approximately 300 ng of peptides were analyzed using an Orbitrap Astral Mass Spectrometer (MS; Thermo Scientific) equipped with a Vanquish Neo UHPLC (Thermo Scientific) using a heated trap and elute setup. The columns were Acclaim PepMap nano-trap column (Dionex C18, 100 Å, 75 µm× 2 cm) and 5.5 cm high throughput µPAC Neo analytical column (Thermo Scientific). The eluents were water with 0.1 % v/v formic acid (solvent A) and 80 % acetonitrile (ACN) with 0.1 % v/v formic acid (solvent B). The peptides were separated on the analytical column using the following gradient at a flow rate of 750 nl/min: (i) 0-0.3 min 3-6% B, ii) 0.3-23 min 6-23.5% B, (iii) 23-26.7 min 23.5-40% B, (iv) 26.7-28.7 min 40-50% B, (v) 28.7-28.8 min 50-99% B and (vi) 28.8-30 min 99 %. The column was then equilibrated for 7 min before the next sample injection. The instrument was configured for data independent acquisition (DIA) where full MS resolutions were set to 120,000 and scanning from 380-980 m/z in profile mode. Full MS automatic gain control (AGC) target was 500 % with a maximum IT of 5 ms. DIA was carried out in the Astral analyzer with an isolation window of 2 m/z, normalized HCD collision energy of 27, normalized AGC target of 500 % and maximum injection time of 3 ms. The cycle time was kept to 0.6 s. Raw data were searched with Spectronaut® software (v.19.0.240606.62635, Huggins, Biognosys) using default settings with the following modifications for increased stringency and peptide quantification: (1) identification setting: exclude single hit proteins was selected, (2) identification setting: protein q-value cut-off was set to 0.01 and precursor and protein posterior error probability (PEP) to 0.01, (3) and quantification setting: Major and Minor Top N groups were unselected to allow for all precursors and peptides that meet the cut-off settings to be identified and quantified.

For F3:II-2, fibroblasts were analyzed using tandem mass tag (TMT)-multiplexing (Thermo Scientific) with mass spectrometry performed at the BayBioMS core facility of the Technical University of Munich, as described by Kopajtich et al.[10] with two minor modifications. Peptide fractionation was conducted using high-pH reverse-phase chromatography (Xbridge BEH 130 C18 3.5µm, 2.1x150m) instead of trimodal mixed-mode chromatography, and TMT labelling was performed using TMT 11-plex reagents (Thermo Scientific) rather than the TMT 10-plex. Data normalization and analysis were carried out using PROTRIDER[13] across a dataset comprising 249 fibroblast samples.

Relative Complex Abundance (RCA) plots were generated using the RCA tool available on the http://www.rdms.app website.[12] Topographical heatmaps were generated using the log2 fold-change between affected individuals, carrier parents, and controls to define the color of the relevant chain in

the cryo electron-microscopy derived structure of Human Complex I[14] (PBD ID: 5LDW) as previously described.[15] Range plots were generated using an R script described in Hock *et al.*[12] to calculate the protein abundance of NDUFA5 in carrier parents (F1:I-1, F1:I-2) relative to the median control abundance alongside the standard deviation (SD).

Respiratory chain enzymology

F2:II-1 skeletal muscle tissue was obtained from the quadriceps by surgical biopsy, and a fibroblast culture was established from skin obtained at the muscle biopsy site. Measurements of skeletal muscle homogenates and fibroblasts were performed as described previously.[16] Individual respiratory chain complex activities and the mitochondrial matrix enzyme citrate synthase (CS) were measured spectrophotometrically in a UV-1601 (Shimadzu) in 1 mL sample cuvettes maintained at 30 °C.[17] Enzyme activities in skeletal muscle from individual F3:II-2 were performed as previously described.[18,19] All values are expressed relative to the mitochondrial marker enzyme CS (mU/mU CS).

Respiratory chain complex assembly

Analysis of mitochondrial proteins was performed on skeletal muscle homogenates and isolated mitochondria from F2:II-1 and control fibroblasts separated by 4.5-13% Blue Native (BN)-polyacrylamide gel electrophoresis (PAGE) as described.[16]. Solubilization of mitochondrial complexes was performed using 5 mg digitonin per mg protein. Subsequent immunoblotting analysis was performed with antibodies against Complex I (NDUFA9; Abcam ab14713), Complex II (SDHA; Abcam ab14715), Complex III (UQCRC2, Abcam ab14745), Complex IV (COX4; Abcam ab33985) and Complex V (ATP5A, Abcam ab14730). For 2D BN-PAGE analysis, the BN-PAGE gel strip was separated on 10 % Tricine/sodium dodecyl sulphate (SDS)-PAGE followed by Coomassie staining. SDS-PAGE was performed as previously described.[20] BN-PAGE analysis of mitochondrial proteins from lymphoblastoid cell lines (LCL) was performed as previously described.[12] Subsequent immunoblotting utilized antibodies against Complex I (NDUFA9, in-house;[21] NDUFB6, in-house[22]), MCIA complex (NDUFAF1, in-house[22]), Complex III (UQCRC1, ThermoFisher 16D10AD9AH5), Complex IV (COX4, ab110261) and the above mentioned Complex II antibody. Protein concentrations were determined by bicinchoninic acid (BCA) assay and equal amounts loaded.

Molecular modeling

To predict the structures of WT and Arg23_Ala61del NDUFA5 the relevant sequences (Uniprot Q16718-1) were uploaded to ColabFold[23] for AlphaFold2 modeling[24] with AMBER relaxation enabled (num_relax=1). All other settings were default. The top-ranked model (pLDDT confidence

score) for each construct was aligned to NDUFA5 extracted from the experimentally determined Cryo-EM structure of human Complex I (PDB:5XTD)[25] using the *super* algorithm within the PyMOL Molecular Graphics System, Version 3.1.6.1, Schrödinger, LLC. Modelling of the quaternary structure of WT and Arg23_Ala61del NDUFA5 in complex with NDUFS2 (Uniprot O75306-1 lacking residues 1-44 containing the mitochondrial targeting sequence [MTS] and region interacting with the membrane arm of Complex I), NDUFS3 (Uniprot O75489-1 lacking MTS residues 1-36 and all residues C-terminal of Glu204 that interact with subunits in the N-module) and NDUFA7 was performed using Alphafold-multimer[26] via Colabfold. To align the modelled quaternary structures with the experimentally determined structure the core complex of NDUFS2, NDUFS3 and NDUFA7 were temporarily merged into a single object in each structure and the *align* algorithm used to determined pairwise RMSDs for each model against the experimental structure.

Zebrafish modeling

Adult zebrafish were housed in a recirculating water system at 28 °C under a 14 h light / 10 h dark photoperiod and fed twice daily. Embryos were obtained via standard breeding protocols.[27] Larvae were raised in embryo medium consisting of 0.03 % Instant Ocean and 0.0002 % methylene blue in reverse osmosis-distilled water. All experimental procedures complied with the Guide for the Care and Use of Animals[28] and adhered to the guidelines of Cipher Gene, Ltd, under the institutional laboratory animal use license (SYXK (Zhe) 2024-0021) granted by Department of Science and Technology of Zhejiang Province, China.

The zebrafish genome contains an ortholog of the human *NDUFA5* gene, designated *ndufa5* (ENSDARG00000039346). Orthology was assessed using the DIOPT Ortholog Finder,[29] which indicated 60 % protein sequence identity between human *NDUFA5* and zebrafish *ndufa5*. Single guide RNA (sgRNA) target sites were predicted using the CHOPCHOP online tool[30] and synthesized by GenScript. Two sgRNAs were designed (PAM sequence in lowercase): GAATCCTCTGCAGACATGACagg, TTTCTGTAAGCGGCGTCCTGggg. For targeted mutagenesis, fertilized embryos at the 1-2 cell stage were injected with ~1 nL of CRISPR complexes containing two sgRNAs (~90 ng/µL each) and Cas9 protein (250 ng/µL). At 24 hours post injection, a subset of injected embryos was pooled for Sanger sequencing to verify the mutagenesis efficacy using the TIDE (Tracking of Indels by DEcomposition) online tool.[31] Post hoc genotyping was performed after phenotypic assessment. Individual larvae were collected for sequencing and TIDE analysis to confirm the mutagenesis, and those with a TIDE efficiency below 5% were excluded from phenotypic data analysis.

For morphological inspection, at 5 days post fertilization (dpf) zebrafish larvae were positioned individually, dorsal side up, in a custom mini-well plate for bright-field imaging. Images were captured using a Touptek CCD camera mounted on a Nikon SMZ800N stereomicroscope at 2× magnification. Morphometric parameters, including eye distance and body length, were quantified using Fiji (ImageJ). Locomotion was studied at 5 dpf. Individual zebrafish larvae were transferred into separate wells of a flat-bottom 96-well microplate containing 200 μL of embryo medium. Locomotor activity was recorded using the DanioVision system with EthoVision XT software (Noldus). Prior to recording, larvae were acclimated for 30 min inside the device at 28 °C under dark conditions. Spontaneous swimming activity was measured for 15 min, followed by alternating dark/light periods (5 min each) to assess stimulus-evoked responses. For each larva, total distance moved and maximum velocity were quantified. As the response had not fully stabilized during the initial cycle, data from the first dark/light cycle were excluded from the analysis. To assess survival, zebrafish larvae were maintained in 100 mm Petri dishes containing 50 mL of embryo medium, with no more than 80 larvae per dish. The dishes were kept in a 28 °C incubator, and 50 % of the medium was replaced daily with fresh medium. Larvae were fed with paramecia every day and monitored twice daily, and dead individuals were removed at each inspection. Mortalities occurring at 0–1 dpf were excluded from the analysis to minimize the influence of injection-related damage.

For electrophysiology studies zebrafish larvae were paralyzed in 100 μM pancuronium (Sigma) and immobilized in 2 % low-melting point agarose within a recording chamber. The chamber was filled with embryo medium and positioned on the electrophysiology platform. Local field potential (LFP) recordings were obtained from optic tectum using a glass microelectrode filled with 2 M NaCl. Signals were amplified using an extracellular amplifier with high impedance head stage (1700, A-M Systems), low-pass filtered at 5 kHz, high-pass filtered at 1 Hz, and digitized at 10 kHz via a digital acquisition board (Measurement Computing). Data acquisition and analysis were performed using the open-source software DClamp. Ictal-like events were defined, as previously described,[32] as multi-spike discharges exceeding 5x baseline noise and lasting more than 500 ms. Interictal-like events were defined as smaller discharges with amplitudes exceeding 3x baseline noise and durations greater than 100 ms. The occurrence of electrographic epileptiform activity, including both ictal- and interictal-like events, was quantified for analysis.

Statistical analyses were performed using Prism 8 (GraphPad Software). Unpaired t tests were used for comparisons between two groups, and log-rank tests were applied for survival analysis. Statistical significance was defined as $^{*}p < 0.05$; $^{**}p < 0.01$; $^{***}p < 0.001$.

Supplemental References

1. Breuss, A., Strasser, M., Nuoffer, J.M., Klein, A., Perret-Hoigne, E., Felder, C., Stauffer, R., Wolf, P., Riener, R., and Gautschi, M. (2024). Nocturnal vestibular stimulation using a rocking bed improves a severe sleep disorder in a patient with mitochondrial disease. J Sleep Res *33*, e14153. 10.1111/jsr.14153.
2. Sadedin, S.P., Dashnow, H., James, P.A., Bahlo, M., Bauer, D.C., Lonie, A., Lunke, S., Macciocca, I., Ross, J.P., Siemering, K.R., et al. (2015). Cpipe: a shared variant detection pipeline designed for diagnostic settings. Genome Med *7*, 68. 10.1186/s13073-015-0191-x.
3. Sadedin, S.P., Ellis, J.A., Masters, S.L., and Oshlack, A. (2018). Ximmer: a system for improving accuracy and consistency of CNV calling from exome data. Gigascience *7*. 10.1093/gigascience/giy112.
4. Zech, M., Kopajtich, R., Steinbrucker, K., Bris, C., Gueguen, N., Feichtinger, R.G., Achleitner, M.T., Duzkale, N., Perivier, M., Koch, J., et al. (2022). Variants in Mitochondrial ATP Synthase Cause Variable Neurologic Phenotypes. Ann Neurol *91*, 225–237. 10.1002/ana.26293.
5. Dobin, A., Davis, C.A., Schlesinger, F., Drenkow, J., Zaleski, C., Jha, S., Batut, P., Chaisson, M., and Gingeras, T.R. (2013). STAR: ultrafast universal RNA-seq aligner. Bioinformatics *29*, 15–21. 10.1093/bioinformatics/bts635.
6. Robinson, J.T., Thorvaldsdottir, H., Winckler, W., Guttman, M., Lander, E.S., Getz, G., and Mesirov, J.P. (2011). Integrative genomics viewer. Nat Biotechnol *29*, 24–26. 10.1038/nbt.1754.
7. Brechtmann, F., Mertes, C., Matuseviciute, A., Yepez, V.A., Avsec, Z., Herzog, M., Bader, D.M., Prokisch, H., and Gagneur, J. (2018). OUTRIDER: A Statistical Method for Detecting Aberrantly Expressed Genes in RNA Sequencing Data. Am J Hum Genet *103*, 907–917. 10.1016/j.ajhg.2018.10.025.
8. Mertes, C., Scheller, I.F., Yepez, V.A., Celik, M.H., Liang, Y., Kremer, L.S., Gusic, M., Prokisch, H., and Gagneur, J. (2021). Detection of aberrant splicing events in RNA-seq data using FRASER. Nat Commun *12*, 529. 10.1038/s41467-020-20573-7.
9. Yepez, V.A., Mertes, C., Muller, M.F., Klaproth-Andrade, D., Wachutka, L., Fresard, L., Gusic, M., Scheller, I.F., Goldberg, P.F., Prokisch, H., and Gagneur, J. (2021). Detection of aberrant gene expression events in RNA sequencing data. Nat Protoc *16*, 1276–1296. 10.1038/s41596-020-00462-5.
10. Kopajtich, R., Smirnov, D., Stenton, S., Loipfinger, S., Meng, C., Scheller, I., Freisinger, P., Baski, R., Berutti, R., Behr, J., et al. Integration of proteomics with genomics and transcriptomics increases the diagnostic rate of Mendelian disorders.

11. Yepez, V.A., Gusic, M., Kopajtich, R., Mertes, C., Smith, N.H., Alston, C.L., Ban, R., Beblo, S., Berutti, R., Blessing, H., et al. (2022). Clinical implementation of RNA sequencing for Mendelian disease diagnostics. Genome Med *14*, 38. 10.1186/s13073-022-01019-9.

12. Hock, D.H., Caruana, N.J., Semcesen, L.N., Lake, N.J., Formosa, L.E., Amarasekera, S.S.C., Stait, T., Tregoning, S., Frajman, L.E., Bournazos, A.M., et al. (2025). Untargeted proteomics enables ultra-rapid variant prioritisation in mitochondrial and other rare diseases. Genome Med *17*, 58. 10.1186/s13073-025-01467-z.

13. Klaproth-Andrade, D., Scheller, I., Tsitsiridis, G., Loipfinger, S., Mertes, C., Smirnov, D., Prokisch, H., Yépez, V., and Gagneur, J. PROTRIDER: Protein abundance outlier detection from mass spectrometry-based proteomics data with a conditional autoencoder.

14. Zhu, J., Vinothkumar, K.R., and Hirst, J. (2016). Structure of mammalian respiratory complex I. Nature *536*, 354–358. 10.1038/nature19095.

15. Stroud, D.A., Surgenor, E.E., Formosa, L.E., Reljic, B., Frazier, A.E., Dibley, M.G., Osellame, L.D., Stait, T., Beilharz, T.H., Thorburn, D.R., et al. (2016). Accessory subunits are integral for assembly and function of human mitochondrial complex I. Nature *538*, 123–126. 10.1038/nature19754.

16. Jackson, C.B., Nuoffer, J.M., Hahn, D., Prokisch, H., Haberberger, B., Gautschi, M., Haberli, A., Gallati, S., and Schaller, A. (2014). Mutations in SDHD lead to autosomal recessive encephalomyopathy and isolated mitochondrial complex II deficiency. J Med Genet *51*, 170–175. 10.1136/jmedgenet-2013-101932.

17. Shepherd, D., and Garland, P.B. (1969). The kinetic properties of citrate synthase from rat liver mitochondria. Biochem J *114*, 597–610. 10.1042/bj1140597.

18. Feichtinger, R.G., Weis, S., Mayr, J.A., Zimmermann, F., Geilberger, R., Sperl, W., and Kofler, B. (2014). Alterations of oxidative phosphorylation complexes in astrocytomas. Glia *62*, 514–525. 10.1002/glia.22621.

19. Feichtinger, R.G., Zimmermann, F., Mayr, J.A., Neureiter, D., Hauser-Kronberger, C., Schilling, F.H., Jones, N., Sperl, W., and Kofler, B. (2010). Low aerobic mitochondrial energy metabolism in poorly- or undifferentiated neuroblastoma. BMC Cancer *10*, 149. 10.1186/1471-2407-10-149.

20. Laemmli, U.K. (1970). Cleavage of structural proteins during the assembly of the head of bacteriophage T4. Nature *227*, 680–685. 10.1038/227680a0.

21. Lazarou, M., McKenzie, M., Ohtake, A., Thorburn, D.R., and Ryan, M.T. (2007). Analysis of the assembly profiles for mitochondrial- and nuclear-DNA-encoded subunits into complex I. Mol Cell Biol *27*, 4228–4237. 10.1128/MCB.00074-07.

22. Dunning, C.J., McKenzie, M., Sugiana, C., Lazarou, M., Silke, J., Connelly, A., Fletcher, J.M., Kirby, D.M., Thorburn, D.R., and Ryan, M.T. (2007). Human CIA30 is involved in the early assembly of mitochondrial complex I and mutations in its gene cause disease. EMBO J *26*, 3227–3237. 10.1038/sj.emboj.7601748.
23. Mirdita, M., Schutze, K., Moriwaki, Y., Heo, L., Ovchinnikov, S., and Steinegger, M. (2022). ColabFold: making protein folding accessible to all. Nat Methods *19*, 679–682. 10.1038/s41592-022-01488-1.
24. Jumper, J., Evans, R., Pritzel, A., Green, T., Figurnov, M., Ronneberger, O., Tunyasuvunakool, K., Bates, R., Zidek, A., Potapenko, A., et al. (2021). Highly accurate protein structure prediction with AlphaFold. Nature *596*, 583–589. 10.1038/s41586-021-03819-2.
25. Guo, R., Zong, S., Wu, M., Gu, J., and Yang, M. (2017). Architecture of Human Mitochondrial Respiratory Megacomplex I(2)III(2)IV(2). Cell *170*, 1247–1257 e1212. 10.1016/j.cell.2017.07.050.
26. Evans, R., O'Neill, M., Pritzel, A., Antropova, N., Senior, A., Green, T., Žídek, A., Bates, R., Blackwell, S., Yim, J., et al. (2021). Protein complex prediction with AlphaFold-Multimer. bioRxiv. 10.1101/2021.10.04.463034.
27. Westerfield, M. (2000). The zebrafish book. A guide for the laboratory use of zebrafish (Danio rerio). 4th ed. (Univ. of Oregon Press, Eugene).
28. Animals, N.R.C.U.C.f.t.U.o.t.G.f.t.C.a.U.o.L. (2011). Guide for the Care and Use of Laboratory Animals, 8th edition (National Academy of Sciences.). 10.17226/12910.
29. Hu, Y., Flockhart, I., Vinayagam, A., Bergwitz, C., Berger, B., Perrimon, N., and Mohr, S.E. (2011). An integrative approach to ortholog prediction for disease-focused and other functional studies. BMC Bioinformatics *12*, 357. 10.1186/1471-2105-12-357.
30. Labun, K., Montague, T.G., Krause, M., Torres Cleuren, Y.N., Tjeldnes, H., and Valen, E. (2019). CHOPCHOP v3: expanding the CRISPR web toolbox beyond genome editing. Nucleic Acids Res *47*, W171–W174. 10.1093/nar/gkz365.
31. Brinkman, E.K., Chen, T., Amendola, M., and van Steensel, B. (2014). Easy quantitative assessment of genome editing by sequence trace decomposition. Nucleic Acids Res *42*, e168. 10.1093/nar/gku936.
32. Griffin, A., Carpenter, C., Liu, J., Paterno, R., Grone, B., Hamling, K., Moog, M., Dinday, M.T., Figueroa, F., Anvar, M., et al. (2021). Phenotypic analysis of catastrophic childhood epilepsy genes. Commun Biol *4*, 680. 10.1038/s42003-021-02221-y.
